# Whole-genome sequence analysis of a Pan African set of samples reveals archaic gene flow from an extinct basal population of modern humans into sub-Saharan populations

**DOI:** 10.1186/s13059-019-1684-5

**Published:** 2019-04-26

**Authors:** Belen Lorente-Galdos, Oscar Lao, Gerard Serra-Vidal, Gabriel Santpere, Lukas F. K. Kuderna, Lara R. Arauna, Karima Fadhlaoui-Zid, Ville N. Pimenoff, Himla Soodyall, Pierre Zalloua, Tomas Marques-Bonet, David Comas

**Affiliations:** 1 Departament de Ciències Experimentals i de la Salut, Institut de Biologia Evolutiva (UPF/CSIC), Universitat Pompeu Fabra, 08003 Barcelona, Spain; 2 Department of Neuroscience, Yale School of Medicine, New Haven, CT USA; 3 CNAG-CRG, Centre for Genomic Regulation (CRG), The Barcelona Institute of Science and Technology, Baldiri Reixac 4, 08028 Barcelona, Spain; 4 Universitat Pompeu Fabra (UPF), Barcelona, Spain; 5 College of Science, Department of Biology, Taibah University, Al Madinah, Al Monawarah Saudi Arabia; 6 Higher Institute of Biotechnology of Beja, University of Jendouba, Avenue Habib Bourguiba, BP, 382, 9000 Beja, Tunisia; 7 Oncology Data Analytics Program, Bellvitge Biomedical Research Institute (ICO-IDIBELL), Consortium for Biomedical Research in Epidemiology and Public Health, Hospitalet de Llobregat, Barcelona, Spain; 8 Department of Archaeology, University of Helsinki, Helsinki, Finland; 9 Division of Human Genetics, School of Pathology, Faculty of Health Sciences, University of the Witwatersrand and National Health Laboratory Service, Johannesburg, South Africa; 10 School of Medicine, The Lebanese American University, Beirut, 1102-2801 Lebanon; 11 Institució Catalana de Recerca i Estudis Avançats, ICREA, 08003 Barcelona, Spain

**Keywords:** Human population genetics, Genome diversity, Whole-genome sequences, Africa, Archaic introgression

## Abstract

**Background:**

Population demography and gene flow among African groups, as well as the putative archaic introgression of ancient hominins, have been poorly explored at the genome level.

**Results:**

Here, we examine 15 African populations covering all major continental linguistic groups, ecosystems, and lifestyles within Africa through analysis of whole-genome sequence data of 21 individuals sequenced at deep coverage. We observe a remarkable correlation among genetic diversity and geographic distance, with the hunter-gatherer groups being more genetically differentiated and having larger effective population sizes throughout most modern-human history. Admixture signals are found between neighbor populations from both hunter-gatherer and agriculturalists groups, whereas North African individuals are closely related to Eurasian populations. Regarding archaic gene flow, we test six complex demographic models that consider recent admixture as well as archaic introgression. We identify the fingerprint of an archaic introgression event in the sub-Saharan populations included in the models (~ 4.0% in Khoisan, ~ 4.3% in Mbuti Pygmies, and ~ 5.8% in Mandenka) from an early divergent and currently extinct ghost modern human lineage.

**Conclusion:**

The present study represents an in-depth genomic analysis of a Pan African set of individuals, which emphasizes their complex relationships and demographic history at population level.

**Electronic supplementary material:**

The online version of this article (10.1186/s13059-019-1684-5) contains supplementary material, which is available to authorized users.

## Background

Paleontological and genetic evidence points towards a recent African origin of anatomically modern humans (AMHs) around 150–300 thousand years ago (kya) and a posterior Out-of-Africa expansion 50–100 kya [1–4]. The specific regions where first modern humans inhabit are still under debate, with northern, eastern, and southern Africa having been proposed as possible locations [4–9]. There is no disagreement, however, about hunting-gathering being the subsistence strategy of all human societies prior to ~ 10 kya [10]. Currently, only a few populations retaining hunter-gatherer lifestyles remain isolated in Africa, including, for example, click-speaking indigenous groups or rainforest hunter-gatherers in Central Africa (aka African Pygmies). These AMH lineages are the most genetically diverse contemporary human populations. They present the most basal lineages of uniparental markers (Y chromosome and mitochondrial DNA) and the deepest branches of our species when considering autosomes [6, 11–15].

Khoisan languages, defined by their use of click consonants as phonemes and by exclusion of the Niger-Kordofanian linguistic family, are spoken by several Khoisan populations who currently reside in the Kalahari regions of Namibia and Botswana in southern Africa, as well as by two other populations in Tanzania, the Hadza and the Sandawe ethnic groups. The basal split of Khoisan people from any other extant human populations has been consistently inferred using uniparental markers [11, 16], microsatellites [17], autosomal neutral regions [14, 15], and whole genomes [18]. Moreover, the study of ancient human demographic history reveals a larger effective population size for the ancestors of Khoisan people compared to the significant decline suffered by non-Khoisan populations after their separation, possibly as a consequence of a drier climate in Western and Central but not in Southern Africa [18].

On the other hand, African Pygmies, broadly characterized by their short statures, include a group of more than 20 culturally heterogeneous populations [19, 20]. As a consequence of their close interactions with neighboring farmers, most Pygmies speak Niger-Kordofanian or Nilo-Saharan languages and had some practice of fishing and agriculture [21]. They are broadly classified in two main groups [22]: Western Pygmies (e.g., Biaka, Baka, Bakola), who inhabit the rainforest west of the Congo Basin, and Eastern Pygmies (e.g., Mbuti, Twa), who live close to the Ituri rainforest and Lake Victoria. Genetic evidence supports an independent origin for all African Pygmies with a basal split from present day agriculturalist populations that is posterior to the Khoisan separation [15, 22–25].

The “Bantu expansion”, which is the migration of Bantu-speaking people from present day Cameroonian Grassfields region close to Nigeria, began around 5–3 kya ago and has been associated with the spread of Late Iron Age culture over most of sub-equatorial Africa [10, 21, 26]. As the migrant Bantu-speakers encountered resident groups in the regions they spread into, varying degrees of admixture ensued with concomitant gene flow between them. In fact, different magnitudes of gene flow with neighboring populations have been reported in several extant Khoisan and Pygmy populations [8, 20, 25, 27, 28]. In addition to the impact these migrations had in eastern and southern Africa, backflow into Africa from Eurasians also influenced the diversity of the African gene pool. For example, low levels of west Eurasian ancestry have been detected in several Khoisan populations, particularly in the Nama but also even in the most isolated groups such as the Ju|‘hoansi [28, 29]. The admixture was dated ~ 1500 kya, prior to the arrival of European colonialist expansion into southern Africa during the eighteenth century, and has been likely introduced from an already admixed population from eastern Africa [29].

Archaic hominins could have also left a footprint in the gene pool of extant populations, which would represent another confounding parameter when analyzing the genetic diversity within the African continent. Initial studies carried out on archaic genomes reported that Neanderthal or Denisovan signatures were found in non-African groups but not in the genomes of sub-Saharan populations [30, 31]. Recent analyses, though, revealed a more complex panorama. Traces of Neanderthal introgression have been observed not only in North African populations [32], who are in fact historically and genetically different from sub-Saharan peoples [33, 34], but also in other African populations, for instance in Yoruba genomes, although they were most likely introduced through recent Eurasian admixture [28, 35, 36]. Furthermore, some evidence of introgression from unknown now-extinct hominins in African groups is accumulating [37–42]. More precisely, archaic introgression has been estimated to be around 5 to 7.9% in Yoruba [37, 42], 2% in Khoisan and Biaka Pygmy [38], and 2% in Hadza, Sandawe, and Western Pygmy populations [39]. Specific candidate introgressed regions have also been identified, for instance, a 20 kbp block found exclusively in sub-Saharan populations that covers the entire *MUC7* gene, a protein abundantly expressed in saliva and associated with the composition of oral microbiome [40], or 265 loci spanning ~ 20 Mbp spread across the genome that were detected in two Western African Pygmy populations [41]. Moreover, the first study with whole-genome sequences from prehistoric Africans suggests the existence of a basal modern human lineage that separated before Khoisan ancestors did and have left asymmetrical signatures on different present day western African populations [43]. An alternative model that also fits their data would involve lasting and long-range gene flow that resulted in eastern and southern Africans being unequally connected to different western African groups. With either model, this study has unraveled that basal diversifications of modern humans were complex. In fact, this complexity is in line with the scenario described in previous studies of several events of gene flow that occurred further back in time among archaic hominins, such as between a population that diverged early from AMHs in Africa and ancestors of the Neanderthals [44, 45] or between unknown archaic hominins and ancestors of Denisovans [36].

A feasible approach to model the complex demographic process that has produced the genetic variation present in current human African populations (including the role of putative archaic introgression from archaic ghost populations) and estimating each of the demographic parameters would be to analyze the data within an Approximate Bayesian computation (ABC) framework. ABC is a statistical framework for inferring the posterior distribution of parameters when the likelihood of the data given the parameters is unknown but there is a way to generate simulated data [46, 47]. The simulator generates new simulated datasets using parameter values from prior distributions. From each simulated dataset, a set of informative summary statistics (SS) for the parameter/model that we are studying is usually computed and compared with the SS computed in the observed data. Finally, the values of the parameters that were used to generate the simulation are accepted or rejected as sampled values from the posterior distribution given an error threshold ε. One of the basic issues of ABC is the definition of “informative SS”; ultimately, SS are dependent on the problem that is being considered and the criteria of the investigator [48]. Following Jiang et al. [49], Mondal et al. [50] recently implemented an ABC with Deep Learning (ABC-DL) framework that allows to estimate the most informative SS for a given problem. A DL can be trained with simulated data using a broad mathematical representation of the genome (such as the multidimensional unfolded join site frequency spectrum (jSFS)) [51] to predict the value of the parameter/model that generated the simulation. Then, the prediction of the DL can be used as the most informative summary statistic (SS-DL) for the parameter/model that is being studied. By applying this new method, Mondal et al. [50] developed a complex demographic model for Eurasian populations and identified the signal of archaic introgression from a ghost population within Asian populations.

Finally, it is worth mentioning that in the scenario described above of admixed societies with complex relationships between themselves throughout their history, pioneering whole-genome sequence studies in African individuals have highlighted the need for a broader geographic sampling coverage across the continent to elucidate the evolutionary history of African populations [18, 28, 39, 43, 52, 53]. The present study adds to the knowledge base of early evolution in Africa through an in-depth analysis of the genomic variation of a collection of whole-genome samples from 15 different African populations, in the process deciphering their elaborated relationships and demographic history, and focusing on the putative introgression from unknown archaic African hominins via the implementation of an ABC-DL approach as in Mondal et al. [50].

## Results

### Dataset and genetic diversity

**Fig. 1:**
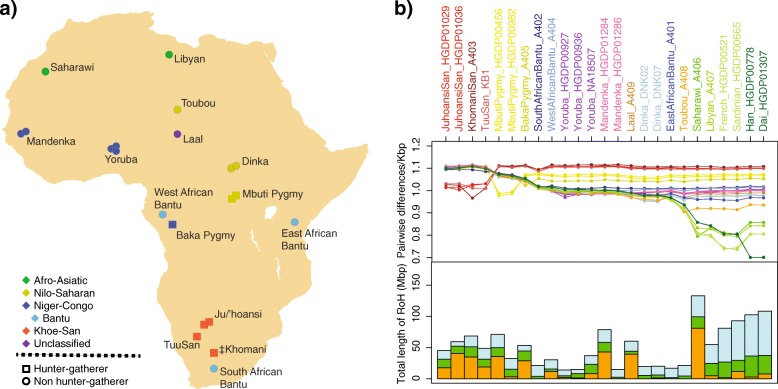
Samples, genetic diversity, and runs of homozygosity. **a** Geographical, linguistic and life-style distribution of African individuals analyzed. **b** On the *top*, pairwise differences per kbp between individuals. Each line corresponds to the genetic differences of a specific individual to the rest of the samples. The line color corresponds to the label color of the individual in the *x* axis. The value given for the same individual is counted considering differences between its two chromosomes. On the bottom, total length of runs of homozygosity per individual. In blue, smaller lengths (from 0.5 to 1 Mbp); in green, intermedium lengths (from 1 to 1.5 Mbp) and in orange, the largest windows (bigger than 1.5 Mbp), the latter are a sign of inbreeding at population or individual level

We collected 21 samples from the four major continental African linguistic groups that belong to 15 different African populations which are either agriculturalists or hunter-gatherers (Fig. 1a). In addition, we included four Eurasian samples for this study. Whole-genome sequencing of the 25 male individuals was conducted on Illumina sequencing platforms. Nine samples were newly sequenced for this project while the whole-genome shotgun read data was already published for the remaining 16 individuals. All samples were paired-end sequence at deep coverage (21–47x) (Table 1, Additional file 1: Table S1.2).

**Table 1:** Samples and sequencing statistics

Individual identifier^1^	Mitochondrial haplogroup	chrY haplogroup	Coverage	#SNPs	#Heterozygous
JuhoansiSan_HGDP01029	L0d1b1	A1b1a1a1	46.63	3,169,565	1,968,088
JuhoansiSan_HGDP01036	L0d1c1a	A1b1b2a	41.34	3,164,150	1,947,901
KhomaniSan_A403 *	L0d2a1	A1b1b2a	23.77	3,142,132	1,877,045
TuuSan_KB1	L0d1b2	B2b1b	25.87	3,157,740	1,961,736
MbutiPygmy_HGDP00456	L0a2b	E1b1a1a1c1a1c	31.25	3,081,528	1,897,510
MbutiPygmy_HGDP00982	L0a2b	E2b1a1	40.13	3,089,676	1,930,933
BakaPygmy_A405 *	L1c1a2b	E1b1a1a1c1a1c	32.38	3,083,814	1,986,951
SouthAfricanBantu_A402 *	L2a1f	E1b1a1a1d1c	22.72	3,001,336	1,972,901
WestAfricanBantu_A404 *	L3d3a1	E1b1a1a1c1a1c	32.53	2,982,337	1,957,325
Yoruba_HGDP00927	L1b1a	E1b1a1a1c1a1	41.93	2,915,392	1,883,193
Yoruba_HGDP00936	L2a12b	E1b1a1a1c1a1	42.78	2,941,205	1,920,680
Yoruba_NA18507	L1b1a3	E1b1a1a1c1a1	43.62	2,934,201	1,912,252
Mandenka_HGDP01284	L2c3a	E1a1	33.39	2,934,343	1,914,085
Mandenka_HGDP01286	L1b1a	E1b1b1a1a1	40.07	2,927,830	1,911,253
Laal_A409 *	L3e1c	B1	25.11	2,916,350	1,899,437
Dinka_DNK02	L2c1	E2a	36.81	2,880,056	1,856,506
Dinka_DNK07	L0a1a	A1b1b2b	46.81	2,880,930	1,844,312
EastAfricanBantu_A401 *	L2a1h	E1b1a1a1c1a1c	21.01	2,893,697	1,917,226
Toubou_A408 *	M1	T1a1	24.79	2,755,888	1,756,695
Saharawi_A406 *	L3b1b1	E1b1b1b1a	24.27	2,525,396	1,545,877
Libyan_A407 *	L2a1c	E1b1b1b1a	25.02	2,540,250	1,609,582
French_HGDP00521	T1a	I1a	35.14	2,398,449	1,434,940
Sardinian_HGDP00665	H3u	I2a1a1	32.55	2,396,919	1,429,346
Han_HGDP00778	A5b1b	O3a2c1a	35.65	2,418,780	1,361,654
Dai_HGDP01307	B4a1c4	O2	35.42	2,406,526	1,362,632

^1^Samples newly sequenced in this study are marked with an *

We detected a total of 12.72 million SNPs in 2 Gbp of callable genome (Additional file 1: Table S2.1). We validated the SNP calling of 21 samples by comparing their genotypes with the ones determined from SNP arrays of these individuals. Twelve HGDP samples were evaluated considering the genotypes generated on an Illumina 650Y array, while the nine genuinely sequenced for this project were genotyped in an Affymetrix’s Genome-Wide Human SNP array 6.0. On average, we achieved a genotype sensitivity of 99.67% for the autosomes, 99.56% for the X chromosome, and a heterozygous sensitivity of 99.37% for the HGDP samples. For the other nine individuals, we achieved an overall genotype sensitivity of 98.70% for the autosomes and 99.22% for the X chromosome. The heterozygous sensitivity for these samples is on average 97.25%.

Hunter-gatherers present the highest genetic diversity of all populations, with Khoisan having greater amount of genetic differences than Pygmies (Fig. 1b top, Additional file 1: Figure S4.1). The four Khoisan samples show similar measures of genetic differences to non-Khoisan samples even belonging to three different groups. Pygmies do not form a single cluster; instead, the Baka Pygmy, in comparison with Mbuti Pygmies, displays less genetic differences to other sub-Saharan and North African populations. Sub-Saharan agriculturalist individuals share highly similar values of genetic diversity relative to all other samples, with lower levels than the ones observed in hunter-gatherers but not as reduced as the non-African samples. The only exception is the Toubou individual, who also maintains similar genetic distance to other sub-Saharan samples but is genetically closer to North African and non-African samples. As expected, North African samples are genetically closer to non-African samples than to sub-Saharan individuals, showing a considerable reduction of genetic diversity.

We determined long homozygous regions, or runs of homozygosity (ROH), of at least 0.5, 1, and 1.5 Mbp of callable genome in each sample (Fig. 1b bottom, Additional file 1: Figure S4.2). Overall, the total length of ROH within a genome depends largely on the geographical origin of the individual; this is, relatively similar values are observed within continents while the amount increase as the distance to Africa gets bigger [54]. However, long ROH over 1.5 Mbp do not follow this geographical tendency. Instead, those segments are more frequent in populations in which isolation and consanguineous unions are more common. We observed that sub-Saharan agriculturalists present the lowest amounts of ROH, whereas both Khoisan and Pygmies show higher levels of ROH that are closer to the ones found in North African or Eurasian populations (Fig. 1b bottom). Moreover, there are three samples (Saharawi, Toubou, and Yoruba_HGDP00927) as well as almost all hunter-gatherers with long ROH, which might indicate in-breeding at the population or individual level.

### Genetic ancestries and gene flow in African individuals

We explored the correspondence between genetic and geographic diversity in our African samples (Additional file 1: Figure S5.1). We obtained a significant correlation between the first two dimensions of a multidimensional scaling analysis from a genetic distance matrix and the coordinates of the sampled individuals in an African map (R = 0.58; *p* value based on 1000 replications = 0.003. Removing Bantu individuals, *R* = 0.655; *p* value based on 1000 replications = 0.001). This correlation suggests that genetics tends to fit the geographic location of the sampled individuals. In fact, we observed that genetic differentiation tends to increase monotonically with geographic distance between individuals (Additional file 1: Figure S5.2), a pattern that is consistent with a main genetic gradient among African populations. Finally, by means of a Bearing procedure [55], we found that the genetic differentiation in the African continent is in the north-west to the south-east axis (Additional file 1: Figure S5.3). This direction is similar to the north to south angle described by [56] using F_st_-based distances and SNP microarray data and is consistent with the Sahara desert acting as a genetic barrier between populations at both sides [56]. The fact that our pattern is somehow rotated could be explained by the particular geographical sampling scheme of our study, which tends to be on the north-west/south-east spatial axis (correlation between latitude and longitude of our sampled locations = − 0.536, *p* value = 0.012).

To define the genetic variation and structure in our dataset, we applied a principal component analysis (PCA) and ran ADMIXTURE [57]. For ADMIXTURE, in order to have more representative samples per population, we downloaded the “Bushman” data library from Galaxy [18, 58]. A total of 374,195 SNPs in 745 samples (the 25 of this study and an additional set of 720 samples from the array that belong to African, European, and Asian populations) were analyzed. We found that seven is the best-supported number of ancestral populations for our data (Additional file 1: Figure S6.2). We named each ancestry after the population/region with the highest proportion of each specific ancestry.

**Fig. 2:**
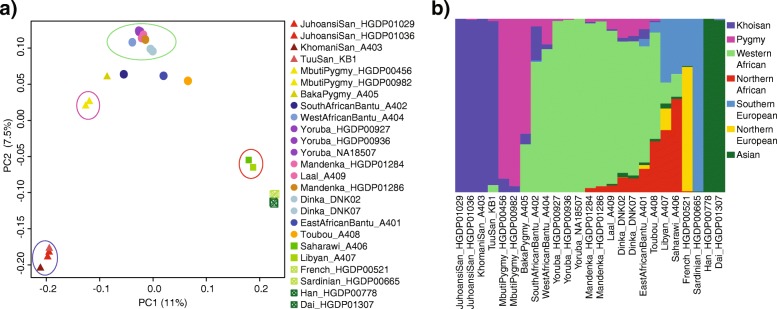
Principal component analysis (PCA) and ADMIXTURE. **a** First two components of a PCA, percentage of explained variance shown in axis; African samples are grouped in four major genetic ancestries, representative samples of each ancestry are shown with a circle colored with its correspondent main genetic ancestry estimated in **b**, North Africans and African samples not circled might be heavily admixed according to **b**; **b** ADMIXTURE plot for the 25 samples in our dataset; the seven ancestries are named according to individuals that have almost exclusively a given ancestry. The plot for the remaining 705 samples is shown in Additional file 1: Figure S6.3

Overall, results from both analyses suggest that African populations can be clustered in four major genetic groups: Khoisan, Pygmy, sub-Saharan agriculturalist, and North Africa (Fig. 2). Consistent with the highest amount of differences observed (Fig. 1b), we found the maximum genetic variance was found between Khoisan and Eurasian populations. With the exception of the Baka individual, the other hunter-gatherer samples in our dataset are mostly represented by a single ancestry; however, it should be noted that the general picture for hunter-gatherers is more complex, with mixed ancestries for most populations (Additional file 1: Figure S6.3). On the other hand, most sub-Saharan agriculturalist individuals present some hunter-gatherer ancestry. The proportion is mainly related to the geographic distance between mixed populations. Dinkas, South African, and West African Bantus present the highest proportions of hunter-gatherer ancestries, and they are geographically the closest populations to Mbuti, Khoisan, and Baka, respectively. The East African Bantu, Laal, and Mandenka individuals show lower proportions of hunter-gatherer ancestries, with values following a dwindling gradient that is concordant with the ascending distance to the Mbuti Pygmy location. Finally, North African samples are closer to Eurasian populations than to any sub-Saharan populations, implying that the Sahara Desert might have represented a major barrier within African populations.

To formally test admixture, we applied the D-statistics test [59] addressing two scenarios: the admixture between hunter-gatherer populations and their respective geographically surrounding agriculturalist populations (South African Bantu for Khoisans; Laal, Toubou, Dinka, and Eastern Bantu for Mbuti Pygmies; Yoruba and Western Bantu for Baka Pygmies), and the putative gene flow from west Eurasian to African populations. Additionally, we evaluated the latter scenario by calculating F_4_-ratio estimates [59], which provide accurate proportions of European ancestry into African populations. The ratios we constructed were f4(Han, Yoruba; X, Chimp)/f4(Han, Yoruba; French, Chimp), being X a hunter-gatherer population, and f4(Sardinian, Han; X, Yoruba)/f4(Sardinian, Han; French, Yoruba) when X refers to other African groups.

We found clear evidence of admixture between Khoisan populations and the South African Bantu individual, as well as between Dinka and Mbuti Pygmies, as this was consistently observed in several comparisons made using different African populations (Additional file 1: Tables S6.1–2). We also detected signatures of gene flow between Mbuti Pygmies and both Chadian individuals (Laal and Toubou), although with lower significance (Additional file 1: Table S6.2). By contrast, East African Bantu, West African Bantu, or Yoruba populations show no evidence of gene flow with their neighbors, Mbuti and Baka Pygmies (Additional file 1: Tables S6.2–3).

As expected, evidence for admixture between west Eurasians (represented by the French sample) and North African populations was formally identified with the D-statistics test (Additional file 1: Table S6.4). We then estimated an F_4_-ratio [29, 59] and obtained a significant proportion of the Eurasian component present in North African populations, with values as high as 84.9% for the Saharawi individual and 76.0% for the Libyan sample (Additional file 1: Table S6.5). Two other northeastern sub-Saharan populations (Toubou and East African Bantu) also stood out with highly significant D-statistics values, although of lower magnitude. This is concordant with an estimated west Eurasian ancestry proportion found of 31.4% and 14.9%, respectively (Additional file 1: Tables S6.4–5). Finally, the three Khoisan groups present significant small proportions (3.83–4.11%) of Eurasian ancestry. This signature, which was estimated with the F_4_-ratio, was not detectable by the D-statistics test (Additional file 1: Tables S6.4–5).

### Effective population size over time

**Fig. 3:**
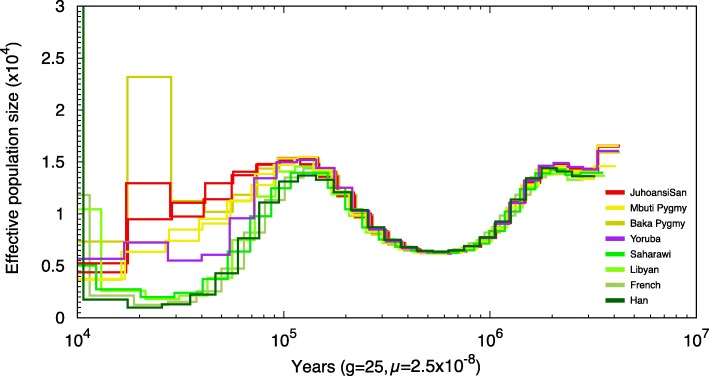
PSMC analyses on eight populations. *N*_e_ and time have been scaled with a mutation rate of 2.5 × 10^−8^ and a generation time of 25 years

To unravel the ancient demographic history of the African populations that are present in our data set, we used the Pairwise Sequentially Markovian Coalescent (PSMC) model that analyzes the dynamics of the effective population size over time [60]. We included at least one representative of each of the 15 African populations and two Eurasian samples in the analysis (Additional file 1: Figure S7.1) and considered both the classical mutation rate of 2.5 × 10^−8^ [61] and the 1.2 × 10^−8^ mutations per bp per generation reported in other analyses [62, 63]. The demographic trajectories of the sub-Saharan agriculturalist populations are very similar to each other; and only South African Bantu and Toubou individuals differ partly from the rest of sub-Saharan farmer samples; however, their considerable levels of admixture with other North African or hunter-gatherer populations (Fig. 2b) might explain this trend. Therefore, in order to ease visualization, we plotted a Yoruba individual (Yoruba_HGDP00936) and two Ju|‘hoansi individuals as representatives of the sub-Saharan agriculturalist and Khoisan populations, respectively (Fig. 3 and Additional file 1: Figure S7.2 considering a mutation rate of 1.2 × 10^−8^).

Our PSMC analysis recapitulated major demographic events that have previously been reported, including a pan-population bottleneck starting around 100 kya [60]. Out-of-Africa populations started to diverge from African populations around 100 to 110 kya and suffered the highest-in-magnitude population reduction, until their recent expansion. Khoisan individuals displayed larger N_e_, maintained through all time periods, as recently reported [18]. We observed that ancestors of Mbuti and Baka Pygmies, like Khoisan, maintained a larger effective population size after the split with non-Khoisan/Pygmy populations. Both Khoisan and Pygmy individuals displayed a moderate population decline compared to Eurasian or North African individuals and also compared to Yoruba, which showed intermediate gradual N_e_ reduction. Interestingly, the Baka Pygmy sample showed a sharp increase in N_e_ around 30 kya. In order to discard a possible spurious increase occurring in one specific time period, we changed time parameters of PSMC to obtain a finer scale. The new estimates revealed a bit more gradual increase spanning three different time intervals (Additional file 1: Figure S7.3). Finally, we also tested to which degree a putative contribution of European ancestry into sub-Saharan African genomes could affect any of the above observations. To that effect, we masked, from the genome of each sub-Saharan individual, all genomic regions of European origin, which we previously inferred with RFMix [64] by considering as reference 922 individuals from African or European populations from the 1000 Genomes Project Phase III panel. We repeated the PSMC on the masked genomes obtaining nearly identical trajectories (Additional file 1: Figure S7.4).

### Archaic introgression from known hominins

Archaic introgression from either known or unknown extinct hominins has been suggested in different African populations [26, 30, 33–39]. In our data, we confirmed previous findings [28–30], as the results of the D-statistics of the form D(X = African population 1, Y = African population 2; Neanderthal/Denisova; Chimpanzee) showed that Eurasian samples as well as North African individuals exhibit a significant enrichment of Neanderthal DNA (higher in East Asia than in West Eurasia or North Africa) when compared to sub-Saharan African samples (Additional file 1: Figure S8.1). Z-score values are generally lower for signatures of Denisovan introgression than for Neanderthal, meaning that a lower proportion of gene flow is observed when admixture has taken place. Asian samples were enriched in archaic DNA from Denisovans, and the European and North African samples too, but at lower levels. This is probably due to the fact that Neanderthal and Denisova are sister groups and consequently share derived alleles that might confound their admixture signals. We found no signals of Neanderthal or Denisovan introgression in the sub-Saharan individuals, which was additionally confirmed with an F_4_-ratio test for the Neanderthal introgression (Additional file 1: Table S8.1).

### Demographic model

We aimed to explore the impact of recent population admixture on the genetic landscape of sub-Saharan populations in an integrative manner, as well as the presence and nature of archaic introgression from hominin populations. To this end, we conducted an Approximate Bayesian Computation (ABC) analysis coupled to a Deep Learning (DL) framework [50] (Additional file 1: Figure S9.1).

**Fig. 4:**
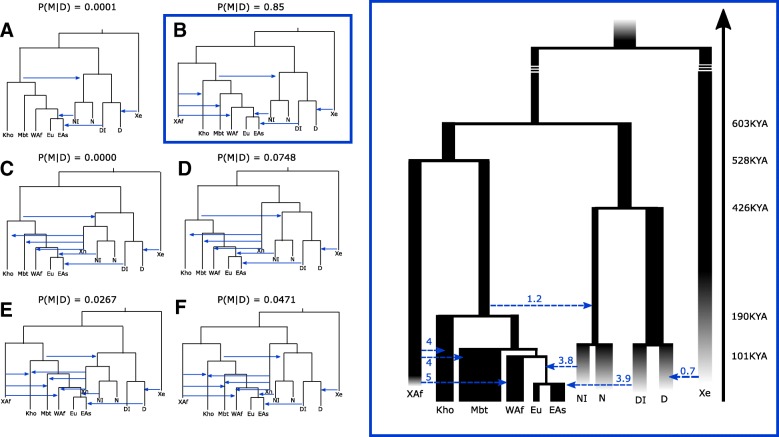
Tested demographic models. Left figures: topology of the demographic models for ABC-DL analyses considering East Asian (EAs), European (Eu), western sub-Saharan (WAf), Mbuti Pygmy (Mbt), and Khoisan (Kho) anatomically modern humans, Altai Neanderthal (N), Neanderthal-like population (NI) with introgressed DNA present in Eurasian populations, Denisova (D), Denisovan-like population (NI) with introgressed DNA present in East Asian populations, an archaic ghost population (Xe) that has left their footprint into Denisovan genome, a putative African extinct basal branch population (XAf), and a second putative archaic ghost population Neanderthal-like (Xn). In all models, recent migrations described in the text are allowed, but not shown in the figure to ease visualization. The posterior probability obtained with our ABC-DL approach is shown for each model; right figure: fitted B model

We implemented six demographic models (Fig. 4; Additional file 1: Table S9.1) of increasing complexity from a basic one (model A). Model A summarizes accepted features of human demography [65]: (*i*) presence of archaic populations out of the African continent, represented by the Neanderthal and Denisovans lineages, (*ii*) introgression from early anatomically modern humans into Neanderthal [44, 45], (*iii*) introgression from an extremely archaic population into Denisovans [36], (*iv*) Khoisans at the root of mankind [11, 14–18], (*v*) Out-of-Africa event of AMHs [3], (*vi*) archaic introgression of a Neanderthal-like population after the Out-of-Africa event in Eurasian populations [30], and (*vii*) archaic introgression from a Denisovan-like population in East Asians [31]. Furthermore, we included recent migrations between Europeans to West Africans, Europeans to Mbutis, Europeans to Khoisans, West Africans to Mbutis, West Africans to Khoisans, Mbutis to West Africans, Mbuti to Khoisans, and Khoisans to Mbutis. These last parameters, as well as the introgression of the archaic population in Denisovans, can be considered as nuisance parameters. Model B extends model A by adding a “ghost” archaic population, XAf, directly related to the lineage leading to AMHs. In this model, XAf independently inbreeds with each of the AMH African populations. Model C extends A by considering that the ghost archaic population is directly related to the Neanderthal lineage, Xn. Model D considers that Xn appears in the archaic lineage out of Africa before the Neanderthal and Denisovan split. Model E is a mixture of model B and C. It considers two ghost archaic populations, one that directly split from the lineage that will produce the AMHs and another related to the Neanderthal lineage, both admixing with AMH populations within Africa. Finally, model F mixes the ghost features of models B and D.

First, we estimated the power of the ABC-DL framework to distinguish among the six considered models by using simulated datasets from known models as observed data and running the ABC-DL framework to estimate the posterior probability of each model. Additional file 1: Table S9.2 shows the confusion matrix for the six models using 100 simulations for each model as observed data. Our analysis suggests that the ABC-DL framework cannot identify all the models with the same accuracy; model F shows the lowest P (real model = X | predicted model by ABC-DL = X) = 0.41, whereas models A, B, C, and D show posterior probabilities of correct assignment > 0.5. This is not surprising given that models E and F are the most general ones. Given these results, we applied the ABC-DL to our observed data. Out of the six considered models, the one showing the largest posterior probability is model B (P (model = B|Data) = 0.85), namely the presence of a ghost archaic population directly related with the lineage that produced the anatomically modern humans. Notably, this posterior probability of model B is 11 times greater than the one from the second most supported model (model D) (P (model = D|Data) = 0.078)), a substantial Bayes factor difference [66] that suggests that the best model out of all the compared ones is model B. Remarkably, basic model A, which does not include any kind of archaic introgression in Africa, has a posterior probability close to 0.

Next, we aimed to estimate the posterior probability of each of the 52 parameters of model B by applying the ABC-DL approach. As a preliminary step, we quantified the performance of the ABC-DL framework in simulated data. For each parameter, we ascertained 1000 simulations at random and estimated the posterior distribution using the ABC-DL. Next, we computed the factor 2 statistic (Additional file 1: Table S9.3), which is the number of times that the estimated mean is within the range 50% and 200% of the true value of the parameter (see Excoffier et al. [67] for details). In 96% of the times, the mean of the posterior distribution of the time of split of XAf with the AMH lineage is within the factor 2, suggesting high confidence in using the mean of this parameter as proxy of the real value. The factor 2 of the amount of introgression of XAf to the different African populations ranges between 77% (XAf to West African) and 72% (XAf to Khoisan) and the times that XAf introgression to the African populations is within the factor 2 range are also ~ 80%, much higher than the expected under randomness. According to the factor 2 analysis, the worse performance of using the mean as a proxy is for migration parameters, which show percentages of factor 2 of ~ 50%, similar to the ones that are observed if the mean of the posterior is sampled at random from the prior distribution. Overall, these analyses support that the mean of the posterior distribution obtained by the ABC-DL framework is a good proxy of the real value used in the simulations for most of the parameters.

Finally, we estimated the posterior distributions of the parameters that describe the most supported demographic model (Fig. 4, Table 2, and Additional file 1: Table S9.4). The ABC-DL produced posterior distributions that strongly deviated from the prior distributions that we considered (see Additional file 1: Figure S9.3) for most of the parameters, suggesting that the ABC-DL approach could properly extract the information present in the observed data to update the prior distributions of each parameter. Not surprisingly, most of the parameters showing posterior distributions similar to the prior distributions are the same that showed low factor 2 values in our former analysis. According to our ABC-DL analyses (Table 2), the AMH lineage and the one from the archaic Eurasian populations diverged 603 kya (95% credible interval (CI) ranging from 495.85 to 796.86 kya). The ghost XAf archaic population and the AMH lineage split 528 kya (95% CI of 230.16 to 700.06 kya), whereas the Denisovan and Neanderthal lineages split 426 kya (95% CI from 332.77 to 538.37 kya). Archaic introgression estimates from XAf to African populations range from 3.8% (95% CI 1.7 to 4.8%) in Khoisan and 3.9% (95% CI 1.3 to 4.9%) in Mbuti to 5.8% (95% CI 0.7 to 0.97%) in West Africa. Our analyses also identified the archaic introgression from early AMHs into Neanderthals (mean of the posterior distribution = 1.2%), yet the 95% CI included 0% (95% CI ranging from 0 to 4%).

**Table 2:** Mean and 95% CI of main parameters of model B

Parameter	Mean	2.50%	97.50%
tAMH-Archaics*	603.25	495.85	796.86
tAMH-XAf*	528.53	230.16	700.06
tN_D*	426.33	332.77	538.37
tAMH*	190.75	160.78	245.12
IntrogressionDI_Han	0.039	0.013	0.049
IntrogressionEarlyHumans_Neanderthal	0.012	0	0.04
IntrogressionNI_Eurasia	0.038	0.017	0.048
IntrogressionXf_Kho	0.041	0.002	0.095
IntrogressionXf_Mbuti	0.043	0.003	0.095
IntrogressionXf_WestAfrica	0.058	0.007	0.097

*kya assuming a generation time of 29 years

The obtained estimates of Neanderthal introgression in Eurasian populations in model B are larger (3.9%, 95% CI from 0.017 to 0.048%) than usually reported. Since sub-Saharan populations are traditionally used as outgroup for detecting archaic introgression out of Africa, we wondered whether these estimated values of archaic introgression in Eurasia could be higher than previously by the fact that we were considering in model B archaic introgression within Africa. We conducted the ABC-DL analysis using the model A, the basic model that does not consider XAf (Additional file 1: Table S9.4). The mean of the posterior distribution of the introgression of Neanderthal ancestry in Eurasian populations was 1.1% (95% CI 0.35 to 3.6%), 3.3 times smaller than that obtained in model B and closer to the range of previously reported values.

## Discussion

The African continent is a melting pot of human cultures and genotypic diversity and, according to current data, the cradle of anatomically modern humans [1–4]. However, despite its crucial importance for understanding recent human evolution, Africans remain underrepresented and understudied in current human datasets [68]. In the present study, we have analyzed the genetic diversity present in genomes sequenced at high coverage in a Pan African set of samples, including a wide geographical, linguistic, and ethnic coverage of human groups in Africa (Fig. 1a).

In agreement with the origin of humans in the African continent and further founder bottlenecks events out of Africa, our PSMC estimates a larger effective population size (*N*_e_) of African samples compared to non-African samples. All hunter-gatherers, not only Khoisan, present higher *N*_e_ along modern-human history than any other population. It is noteworthy that we observed by PSMC a sudden *N*_e_ increase in Baka Pygmy around 30 kya. A similar increase was observed in another study that analyzed several Baka and Biaka samples [25]. In addition, this individual presents the highest average genome-wide heterozygosity compared to the rest of samples (Fig. 1b). Nevertheless, such abrupt *N*_e_ increase can be attributed to either a population expansion or episodes of separation and admixture [60]. Further analyses at population level are needed to distinguish between these two scenarios.

The African genetic landscape derived from our analyses (genetic diversity, ROH, PCA, and ADMIXTURE) reveals four major genetic human groups in Africa, associated to geographic and cultural/linguistic groups and comprising Khoisan, Pygmies, sub-Saharan non-hunter-gatherers, and North African populations. While different hunter-gatherer groups show more differentiation compared to the rest of samples, agriculturalist sub-Saharan individuals are genetically more homogeneous, most likely due to the Bantu expansion. Northern African individuals are closely related to non-African populations, in agreement with a recent split of both groups and continuous gene flow, as clearly determined with D and F_4_-ratio statistics. Therefore, the Mediterranean Sea is pinpointed as an incomplete genetic barrier between Africa and Eurasia, whereas the Sahara Desert represents a major barrier within Africa. Nevertheless, we observed that genetic diversity among samples decays mainly with geographical distance, underlying the role of isolation by distance as a major force in shaping genetic differentiation in Africa [56]. These four major groups, along with African populations in general, are not isolated. Indeed, we discerned migration permeability between specific African populations, mostly associated to geographic proximity. Moreover, we found three samples (Saharawi, Toubou, and Yoruba_HGDP00927) with signs of inbreeding. Further analyses with more samples are needed in order to estimate the extent of inbreeding in these populations.

Compelling evidence accumulates in favor of interbreeding between early hominin species being common instead of exceptional. Neanderthal and Denisovan introgression in Asia, Europe, and North Africa has been well established in previous studies [30–32] and confirmed in our data with a D-statistics analysis. Although the poor DNA preservation in ancient samples hinders direct analyses [69], indirect evidence increasingly supports the contribution of unknown now-extinct hominins to the African genetic pool in sub-Saharan Africa [28, 35–42], where the ancestors of modern humans coexisted during the Pleistocene with different archaic humans [41]. Our ABC-DL analysis is a new incorporation to this bulk of indicia. Indeed, it corroborates that a model in which there is no archaic introgression is extremely unlikely, as was previously observed in [38]. Applying this novel strategy that includes a trained machine learning algorithm as first step, the output of which we used in the ABC analysis, we have been able to inquire complex models circumventing the demanding computational requirements for modeling such complex scenarios.

Our results suggest interbreeding of AMHs with an archaic ghost population that diverged from the AMH lineage at a temporal scale similar to the one between the Neanderthals and Denisovans. This observation would indicate the presence of a deep archaic population substructure also in the African continent and contrasts with previous studies that suggested that a basal lineage had a major impact only on particular western African populations [43]. Furthermore, our analyses showed that the estimated proportion of Neanderthal ancestry in Eurasian populations is highly sensitive to the presence of XAf population, increasing by a threefold the amount of archaic introgression. This result suggests that the amount of Neanderthal ancestry out of Africa that so far has been estimated could be an underestimation by not having considered events of archaic introgression in Africa in the tested models.

## Conclusions

We have comprehensively analyzed the genetic relationships among a Pan African set of human genomes sequenced at high coverage. By implementing novel methodologies when necessary, we have assessed demographic population changes and recent admixture between their populations, as well as, archaic interbreeding with other hominins. Our data point to a complex demographic scenario within Africa related to the complex history of AMHs.

## Materials and methods

### Samples and genotyping

We sequenced nine blood samples from African origin (Table 1) on an Illumina HiSeq2000 sequencing platform. All subjects gave written informed consent and all experimental methods performed comply with the Helsinki Declaration. We downloaded whole-genome sequence data of another 16 individuals from the Sequence Read Archive (SRA, http://www.ncbi.nlm.nih.gov/sra) (accession numbers are SRX015734, SRX016231, and SRX103808) and from cdna.eva.mpg.de/neandertal/altai/ModernHumans/bam. All sequences were sequenced at deep coverage (21–47x) (see Additional file 1: section S1 for extended information).

Single-nucleotide polymorphism (SNP) genotyping calling of each sequenced sample in autosomal and sexual chromosomes was performed by means of a stringent procedure. Briefly, we mapped the paired-end reads of each sample against the human assembly GRCh37 using the BWA aligner [70]; removed PCR duplicates using MarkDuplicates from Picard tools (http://broadinstitute.github.io/picard); realigned regions around indels, recalibrated base qualities, called genotypes, and filtered variants by quality using GATK [71] and VQSR [71]. Furthermore, we determined the callable genome, portion of the genome with confident genotypes, as follows: each callable locus should have at least five reads high-quality mapped in all samples; and repetitive, duplicated, and indel regions were discarded. We detected 12.72 million SNPs in 2 Gbp of callable genome (see Additional file 1: section S2 for extended information).

We reconstructed the complete mitochondrial sequences of all individuals using a procedure that was previously published [72]. Remarkably, a comparison of the sequences obtained via both the traditional Sanger sequencing and this method resulted in a 100% of identity. In short, for each sample, we retrieved the mitochondrial reads from the whole set of shotgun paired-end reads by mapping with BWA [70] against the human mitochondrial reference genome [73], retaining only high-quality paired-end reads. We used Hapsembler [74] to reconstruct the complete sequence after reducing the number of reads per sample to around 350X of mitochondrial coverage (except for the TuuSan KB1 sample for which the resampling was done at 300X). We repeated the reconstruction 20 times to compensate the previous randomization and, thus, to avoid possible assemblage of *numts*. On the other hand, to improve the sequence reconstruction at the extremes of the reference assembly, we repeated the same procedure but mapping against a reference genome with a modified origin (8 kbp from the reference origin). Consensus mitochondrial sequence for each individual was constructed from the de novo assembled 40 mitochondrial assemblies. Mitochondrial haplogroups were then determined by locating sample variants in the updated mitochondrial phylogenetic tree available in www.phylotree.org (see Additional file 1: section S3 for extended information).

In the Y chromosome, we analyzed nine high-quality regions described by Wei et al. [75], which span 8.97 Mbp and are the result of excluding the pseudoautosomal, heterochromatic, X-transposed, and ampliconic segments from the male specific region of the Y chromosome [75, 76]. By intersecting with our callable genome, we got a final set of 3259 SNPs in 3.44 Mbp of genomic sequence, which we used to identify the Y chromosome haplogroup for each sample with the AMY-tree software v2.0 [77] (see Additional file 1: section S3 for extended information).

### Quality assessment

We analyzed the level of concordance between the callable inferred genotypes and microarray-based genotypes called on same samples. Genotypes generated on Illumina 650Y arrays of the 12 HGDP samples were downloaded from http://hagsc.org/hgdp/files.html. After stringent SNP matching and cleaning procedures, 558,832 SNPs out of the 644,258 autosomal SNPs and 8948 SNPs in the X chromosome (54.32% of the initial SNPs) were considered for comparison. Genotypes generated on Affymetrix’s Genome-Wide Human SNP array 6.0 were compared with our calls for additional nine samples. After stringent data management, we retained a shared set of 734,734 SNPs for validation, of which 19,472 SNPs belong to the X chromosome (53.71% of the initial set) and 110 SNPs to the Y chromosome (39.86% of the initial set). Genotype sensitivity was assessed as the proportion of alleles having the same genotype in both sets over the total set of alleles under evaluation (see Additional file 1: section S2 for extended information).

### Statistical data analyses

Genetic diversity was estimated by computing the proportion of different genotypes per kbp between every two individuals. To do that, one of the two alleles was randomly chosen in each locus. If two individuals belong to the same population, this is a measure of heterozygosity within the population. Similarly, heterozygosity for each individual was computed by comparing both alleles in each locus. Runs of homozygosity (ROH) were computed by counting the number of heterozygous genotypes present in 1 kbp of callable genome and identifying continuous windows with less than 10% of the expected heterozygosity and spanning more than 0.5, 1, and 1.5 Mbp. We assumed an average heterozygosity of 1 per kbp to calculate the expected heterozygosity in a region and imposed that at least 67% of the total length of the ROH had to belong to the callable genome (see Additional file 1: section S4 for extended information).

Spatial dependence of the genetic ancestry of the sampled populations was estimated by means of a Procrustes analysis [78] between the geographic coordinates and the first two coordinates from a classical multidimensional scaling (MDS) computed with an identical by state (IBS) distance matrix between pair of individuals. We assessed the genetic differentiation relative to geographic distances via a Mantel correlogram implemented in PASSAGE 2.0 [79]. Finally, the maximum angle of genetic differentiation between populations was computed by means of a Bearing procedure [55], also implemented in PASSAGE 2.0 [79] (see Additional file 1: section S5 for extended information).

We performed a principal component analysis (PCA) using prcomp function in R and considering all autosomal SNPs that were not fixed for the alternative allele. To run ADMIXTURE [57], we increased our dataset by including the “Bushman” dataset available in Galaxy [18, 58]. A total of 376,195 SNPs included in the callable genome in 745 individuals from targeted populations was analyzed. Gene flow between hunter-gatherers and their surrounding populations, as well as between west Eurasians and African populations, was formally tested using the D-statistics implemented in ADMIXTOOLS 4.1 software [59]. The proportion of admixture from Eurasian to African populations was furthermore estimated applying a modified F_4_-ratio test, also using ADMIXTOOLS 4.1 software [29, 59]. Statistical significance was estimated by means of a weighted block jackknife [80] (see Additional file 1: section S6 for extended information).

We estimated the effective population size through time of each population by applying the Pairwise Sequentially Markovian Coalescent (PSMC; [49]) model to our genomes (one representative sample per population), considering only callable positions with not extreme read depth. Mutation rates used are 2.5 × 10^−8^ and 1.2 × 10^−8^ per generation, scaling time using 25 years as generation time (see Additional file 1: section S7 for extended information).

We tested for Neanderthal and Denisovan introgression into our whole set of African populations by means of D-statistics, using the ADMIXTOOLS 4.1 software [48]. Additionally, an F_4_-ratio statistics was calculated as f_4_(Denisova, Chimp; X = African population, Yoruba)/f_4_(Denisova, Chimp; Neanderthal, Yoruba) to estimate the proportion of Neanderthal ancestry present in the X sample. The computation was also performed through the ADMIXTOOLS 4.1 software [48] (see Additional file 1: section S8 for extended information).

In order to compare complex demographic models involving the presence of introgression in the AMH lineage of archaic ghost populations and to estimate the posterior distributions of the parameters of a given model, we used Approximate Bayesian Computation with a Deep Learning step for identifying the most informative summary statistics (SS-DL; see Additional file 1: Figure S9.1). The method is explained in detail in Mondal et al. [50]. Briefly, in the current implementation of the ABC-DL for demographic inference, we consider the genomic joint multidimensional site frequency spectrum among populations (jSFS). This statistic contains the information required to run most of the commonly frequency-based statistics used in population which are informative for detecting most of the demographic parameters considered in the models (see [50]). Next, we train a DL to predict from the jSFS for each parameter or set of models, and we define this prediction as the most informative summary statistic (SS-DL) of the considered parameter or set of models. A potential caveat of this approach is the fact that the DL is trained with data generated from simple models compared to the real model that generated the observed data. To avoid biases in the DL prediction of the parameters/models phase, we assume that the model that generated the data is a generalization of one of the considered demographic models. This assumption is included in the DL by means of injecting jSFS noise in each simulation from the real data (see [50]). Finally, we perform the classical ABC approach using the SS-DL in a new set of simulated datasets.

We tested six different demographic models, inquiring introgression from archaic ghost populations and recent admixture from Eurasian populations into African populations as well as migration within African populations. Data was generated with fastsimcoal2 [81] on 11,642 fragments comprising 393.5 Mbp of callable genomic regions after excluding genes and CpG islands. For model comparison, we developed 10 DL networks with four hidden layers each one. Each network was trained with 15,000 simulations per model (comprising a total of 90,000 simulations), setting as output for each simulation the assignation of one of the six models. Each simulation was injected with noise from the observed jSFS from Altai Neanderthal, Denisovan, HGDP00778, HGDP00521, HGDP01284, HGDP00456, and HGDP01029. Next, we generated an additional set of 150,000 simulations per model, injected noise from the same individuals, and predicted for each simulation in each of the 10 DL the probability of assignation to each model. A combined model prediction was obtained by averaging over the 10 predictions. This combined prediction was used as the SS-DL for the ABC analysis. As observed data for the ABC analysis, we considered Altai, Denisovan, HGDP00778, HGDP00521, HGDP01286, HGDP00982, and HGDP01036. For each independent parameter, we trained 10 independent DL network using 20,000 simulations, and we ran ABC on an additional set of 150,000 simulations. Next, we computed a Spearman correlation between the parameter prediction of each of the 10 DL and the parameter used in the additional simulations, and ascertained the DL for each parameter showing the highest correlation. This DL was used for generating the SS-DL for parameter estimation (see Additional file 1: section S9 for extended information).

## Additional files

Additional file 1: Supplemental material and methods. (PDF 2490 KB)
