# Supplementary material for: Whole-genome sequence analysis of a Pan African set of samples reveals archaic gene flow from an extinct basal population of modern humans into sub-Saharan populations
Source: Genome Biol. 2019 Apr 26;20:77. doi: 10.1186/s13059-019-1684-5 (PMC6485163; doi:10.1186/s13059-019-1684-5)
Supplement: Supplementary file 1 — Supplemental material and methods. (PDF 2490 KB) [file 13059_2019_1684_MOESM1_ESM.pdf]

# Supplemental Material and Methods


**S1) Sample collection and sequencing** **2**

**S2) SNP calling** **4**

*Validation* *6*

**S3) Mitochondrial and Y-Chromosome analysis** **9**

*Mitochodrial reconstruction* *9*

*Y Chromosome reconstruction* *10*

*Haplogroups and phylogenetic analysis* *11*

**S4) Genetic diversity and runs of homozygosity** **16**

*Pairwise differences* *16*

*Runs of homozygosity (ROH)* *18*

**S5) Spatial analyses** **19**

**S6) Genetic structure and admixture tests** **22**

*Principal Component Analysis (PCA)* *22*

*ADMIXTURE* *23*

*D-statistics and $F_4$-ratio estimation* *24*

**S7) PSMC** **30**

**S8) Neanderthal and Denisova introgression** **34**

**S9) Demographic model** **37**

**References** **63**

## S1) Sample collection and sequencing

Twenty-five human DNA samples were collected: 21 from African volunteers and 4 of Eurasian origin. Nine of the samples are newly sequenced in this project with their DNA obtained from blood. We supplemented our dataset with sequencing data of 16 individuals whose whole-genome shotgun read data was already published. We downloaded this data from the Sequence Read Archive (SRA, http://www.ncbi.nlm.nih.gov/sra; accession numbers are SRX015734, SRX016231, and SRX103808) and from cdna.eva.mpg.de/neandertal/altai/ModernHumans/bam. The DNA extraction of the published genomes was carried out from lymphoblastoid cell cultures except for three samples, Dinka DNK02 and DNK07 (from mouthwash sample), and TuuSan KB1 (from blood) (**S1.1 Table**).

### S1.1 Table. Sample collection

| Individual identifier | Language Family | Geographic location | DNA source | Accession number[a] |
|---|---|---|---|---|
| JuhoansiSan_HGDP01029 | Khoe-San | Namibia | Lymphoblastoid | SRX103808 |
| JuhoansiSan_HGDP01036 | Khoe-San | Namibia | Lymphoblastoid | BAM |
| KhomaniSan_A403 | Khoe-San | South Africa | Blood | |
| TuuSan_KB1 | Khoe-San | Namibia | Blood | SRX015734 |
| MbutiPygmy_HGDP00456 | Nilo-Saharan | DR Congo | Lymphoblastoid | SRX103808 |
| MbutiPygmy_HGDP00982 | Nilo-Saharan | DR Congo | Lymphoblastoid | BAM |
| BakaPygmy_A405 | Niger-Congo | Gabon | Blood | |
| SouthAfricanBantu_A402 | Niger-Congo | South Africa | Blood | |
| WestAfricanBantu_A404 | Niger-Congo | Gabon | Blood | |
| Yoruba_HGDP00927 | Niger-Congo | Nigeria | Lymphoblastoid | SRX103808 |
| Yoruba_HGDP00936 | Niger-Congo | Nigeria | Lymphoblastoid | BAM |
| Yoruba_NA18507 | Niger-Congo | Nigeria | Lymphoblastoid | SRX016231 |
| Mandenka_HGDP01284 | Niger-Congo | Senegal | Lymphoblastoid | SRX103808 |
| Mandenka_HGDP01286 | Niger-Congo | Senegal | Lymphoblastoid | BAM |
| Laal_A409 | Unclassified | Chad | Blood | |
| Dinka_DNK02 | Nilo-Saharan | Southern Sudan | Mouthwash | SRX103808 |
| Dinka_DNK07 | Nilo-Saharan | Southern Sudan | Mouthwash | BAM |
| EastAfricanBantu_A401 | Niger-Congo | Kenya | Blood | |
| Toubou_A408 | Nilo-Saharan | Chad | Blood | |
| Saharawi_A406 | Afro-Asiatic | Western Sahara | Blood | |
| Libyan_A407 | Afro-Asiatic | Libya | Blood | |
| French_HGDP00521 | Indo-European | France | Lymphoblastoid | SRX103808 |
| Sardinian_HGDP00665 | Indo-European | Sardinia | Lymphoblastoid | SRX103808 |
| Han_HGDP00778 | Sino-Tibetan | China | Lymphoblastoid | SRX103808 |
| Dai_HGDP01307 | Tai | China | Lymphoblastoid | SRX103808 |

[a] For the previously published genomes, the accession number for retrieval from SRA is also shown; BAM indicates that a bam file is available at cdna.eva.mpg.de/neandertal/altai/ModernHumans/bam

Both published and unpublished samples were paired-end sequenced on an Illumina HiSeq2000 sequencing platform, with the exception of individuals Yoruba NA18507 and TuuSan KB1, that were sequenced using Illumina Genome Analyzer II. Pooled libraries were prepared for fourteen individuals [1, 2]. We identified the pair reads from each sample by using the program fastx_barcode_splitter.pl from the fastx_toolkit package (http://hannonlab.cshl.edu/fastx_toolkit/index.html), that recognizes the individual barcodes, which in this case were incorporated in the first read. We allowed up to one mismatch. Subsequently, we removed the barcodes from the first read, and as a result, we got paired reads of 94/100 or 95/101 bps (read1/read2). The remaining samples, for which a self-library was prepared, have pairs of 100 or 101 bps each, except for the TuuSan KB1, which has shorter reads of 76 bps, and the Yoruba individual NA18507, which has paired reads of 100/102 bps. All samples were sequenced at deep coverage (21-47X) (**S1.2 Table**).

**S1.2 Table. Sequencing details per sample.**

| Individual identifier | Illumina platform | Read length[a] | Number of reads | Coverage[b] |
|---|---|---|---|---|
| JuhoansiSan_HGDP01029 | HiSeq2000 | 94/100, 95/101 | 1,486,634,084 | 46.63 |
| JuhoansiSan_HGDP01036 | HiSeq2000 | 100/100 | 1,282,201,292 | 41.34 |
| KhomaniSan_A403 | HiSeq2000 | 100/100 | 737,196,680 | 23.77 |
| TuuSan_KB1 | Genome Analyzer II | 76/76 | 1,055,649,286 | 25.87 |
| MbutiPygmy_HGDP00456 | HiSeq2000 | 94/100, 95/101 | 995,091,064 | 31.25 |
| MbutiPygmy_HGDP00982 | HiSeq2000 | 100/100 | 1,244,819,468 | 40.13 |
| BakaPygmy_A405 | HiSeq2000 | 100/100 | 1,004,324,132 | 32.38 |
| SouthAfricanBantu_A402 | HiSeq2000 | 100/100 | 704,718,266 | 22.72 |
| WestAfricanBantu_A404 | HiSeq2000 | 100/100 | 1,008,889,104 | 32.53 |
| Yoruba_HGDP00927 | HiSeq2000 | 94/100, 95/101 | 1,335,488,090 | 41.93 |
| Yoruba_HGDP00936 | HiSeq2000 | 100/100 | 1,326,976,006 | 42.78 |
| Yoruba_NA18507 | Genome Analyzer II | 100/102 | 1,339,740,542 | 43.62 |
| Mandenka_HGDP01284 | HiSeq2000 | 94/100, 95/101 | 1,063,283,892 | 33.39 |
| Mandenka_HGDP01286 | HiSeq2000 | 100/100 | 1,242,847,362 | 40.07 |
| Laal_A409 | HiSeq2000 | 101/101 | 771,225,174 | 25.11 |
| Dinka_DNK02 | HiSeq2000 | 94/100, 95/101 | 1,172,305,034 | 36.81 |
| Dinka_DNK07 | HiSeq2000 | 100/100 | 1,451,872,516 | 46.81 |
| EastAfricanBantu_A401 | HiSeq2000 | 100/100 | 651,833,278 | 21.01 |
| Toubou_A408 | HiSeq2000 | 101/101 | 761,383,562 | 24.79 |
| Saharawi_A406 | HiSeq2000 | 100/100 | 752,814,818 | 24.27 |
| Libyan_A407 | HiSeq2000 | 100/100 | 775,988,282 | 25.02 |
| French_HGDP00521 | HiSeq2000 | 94/100, 95/101 | 1,118,760,228 | 35.14 |
| Sardinian_HGDP00665 | HiSeq2000 | 94/100, 95/101 | 1,036,669,814 | 32.55 |
| Han_HGDP00778 | HiSeq2000 | 94/100, 95/101 | 1,135,180,586 | 35.65 |
| Dai_HGDP01307 | HiSeq2000 | 94/100, 95/101 | 1,128,053,978 | 35.42 |

[a] Read length is shown as pair1/pair2 length
[b] Coverage relative to the length of the human assembly GRCh37 without chrM (*3,101,788,170 bps)*

## S2) SNP calling

For each sample we mapped the paired-end reads against the human assembly GRCh37 using the BWA aligner (version 0.6.1, parameters `aln –n 6 –q 15 and sampe`) [3]. As some samples were extracted from lymphoblastoid cell cultures (**S1.1 Table**), we included the Epstein Barr virus into the reference genome. We removed the PCR duplicates using the program MarkDuplicates.jar from Picard tools (version 1.70) (http://picard.sourceforge.net/). Afterwards, we applied a local improvement of the alignments around indels and a base quality recalibration, called genotypes and filtered variants by quality using GATK version 2.5-2 [4]. Commands used in GATK are shown below; required files were downloaded from GATK server.

```
-T RealignerTargetCreator --known 1000G_phase1.indels.b37.vcf --known
Mills_and_1000G_gold_standard.indels.b37.vcf
-T IndelRealigner
-T BaseRecalibrator -knownSites dbsnp_137.b37.vcf -knownSites
1000G_phase1.indels.b37.vcf –knownSites
Mills_and_1000G_gold_standard.indels.b37.vcf
-T UnifiedGenotyper --dbsnp dbsnp_137.b37.vcf -stand_call_conf 20 -
stand_emit_conf 20 -glm SNP
-T VariantRecalibrator -resource:hapmap,VCF,known=false,
training=true,truth=true,prior=15.0 hapmap_3.3.b37.vcf -resource:
omni,VCF,known=false,training=true,truth=true,prior=12.0
1000G_omni2.5.b37.vcf -resource:1000G,VCF,known=false,
training=true,truth=false,prior=10.0
1000G_phase1.snps.high_confidence.b37.vcf -resource:dbsnp,VCF,
known=true,training=false,truth=false,prior=2.0 dbsnp_137.b37.vcf  -an
QD -an HaplotypeScore -an MQRankSum -an ReadPosRankSum -an FS -an MQ -
mode SNP --TStranche 100.0  --TStranche 99.9  --TStranche 99.5  --
TStranche 99.0  --TStranche 98.0
-T ApplyRecalibration --ts_filter_level 99
```

We kept SNPs that passed the VQSR filters and are not multiallelic, focusing from now on chromosomes 1 to 22, X and Y.

To identify not only confident variants but also non-variable sites, we determined the portion of the genome that is callable applying two criteria to each genomic position: i) at least 5 reads should map with no poor mapping quality and ii) the minimum calling confidence threshold used for previously identified variants was used for the rest of the genome. Finally, we imposed a conservative criterion: a position is callable if it is callable in all samples. We used GATK (version 2.5-2) to get the callable regions for each sample with the following commands.

```
-T CallableLoci -minDepth 5
```

```
-T UnifiedGenotyper --dbsnp dbsnp_137.b37.vcf -out_mode EMIT_ALL_SITES
-stand_call_conf 20 -stand_emit_conf 20 -glm SNP
```

Furthermore, we excluded repetitive, duplicated and indel sequences as they are prone to errors when calling SNPs. We joined and applied a 6 bps padding to: i) repeats longer than 80 bps reported from TandemRepeatMarker (downloaded from UCSC); ii) repeats detected with RepeatMasker (downloaded from UCSC) with at least 80 bps of length and being as much as 10% different from the reference; iii) segmental duplications described in hg19 (downloaded from UCSC); and iv) copy number variants reported in the 1000G (downloaded from http://www.ncbi.nlm.nih.gov/dbvar/studies/, study variants_for_estd199.csv). Finally, we called indels with GATK (version 2.5-2, `-T UnifiedGenotyper --dbsnp dbsnp_137.b37.vcf -stand_call_conf 20 -stand_emit_conf 20 -glm INDEL`) [4] for exclusion within 6 bps of flanking sequence.

In total, we detected 12.72 million SNPs in 2 Gbp of callable genome (**S2.1 Table**). As expected, the transition/transversion ratio observed is 2.14 at the genome level. Focusing on the 70,405 SNPs within coding sequences (as described in RefSeq database on July 18th 2013), we got a transition/transversion ratio of 3.32, which is also a signal of good quality SNPs.

**S2.1 Table. SNP calling statistics by chromosome, indicating the number of callable base-pairs, the number of recovered SNPs in the callable base-pairs and the transition/transversion (Ti/Tv) ratio.**

| Chr | # Callable bps | # SNPs | $T_i/T_v$ |
|---|---|---|---|
| 1 | 162,942,189 | 939,618 | 2.2269 |
| 2 | 178,449,618 | 1,063,892 | 2.1164 |
| 3 | 147,411,672 | 885,516 | 2.1035 |
| 4 | 137,943,057 | 872,834 | 2.0896 |
| 5 | 132,839,398 | 804,973 | 2.1115 |
| 6 | 126,431,654 | 773,414 | 2.1905 |
| 7 | 104,643,362 | 657,003 | 2.1196 |
| 8 | 107,632,222 | 707,030 | 1.9797 |
| 9 | 80,524,364 | 521,590 | 2.0310 |
| 10 | 95,064,180 | 601,609 | 2.2235 |
| 11 | 94,408,831 | 577,039 | 2.1495 |
| 12 | 97,484,884 | 570,332 | 2.2051 |
| 13 | 71,302,966 | 443,325 | 2.1591 |
| 14 | 64,870,798 | 393,926 | 2.1953 |
| 15 | 56,845,487 | 353,119 | 2.1361 |
| 16 | 53,632,247 | 381,377 | 1.8896 |
| 17 | 51,297,562 | 299,284 | 2.3920 |
| 18 | 56,808,822 | 358,951 | 2.2002 |
| 19 | 30,458,245 | 193,153 | 2.4045 |
| 20 | 45,871,737 | 283,469 | 2.3431 |

| | | | |
|---|---|---|---|
| 21 | 23,967,970 | 161,644 | 2.1966 |
| 22 | 21,461,321 | 139,113 | 2.4191 |
| **Autosomal** | **1,942,292,586** | **11,982,211** | **2.1444** |
| X | 60,382,834 | 185,719 | 2.0499 |
| Y | 4,249,149 | 4,088 | 1.7923 |
| **All** | **2,006,924,569** | **12,172,018** | **2.1428** |

## Validation

For the twelve HGDP samples (**S2.2 Table**), we downloaded the genotypes generated on Illumina 650Y array from http://hagsc.org/hgdp/files.html for comparison. Out of the 644,258 autosomal SNPs, we first discarded those that are not univocally identified in dbSNP v126 either because the same position is included with different identifiers in the database or because they are included within an indel (1,821 SNPs rejected). We then applied a liftOver to hg19 (tool and chain files downloaded from http://hgdownload.cse.ucsc.edu/downloads.html), keeping only SNPs in hg19 that when going back to hg18 correspond to the original coordinate and have the same allele in both references (530 SNPs excluded in this step). We finally imposed that the 12 samples have no missing genotype (additional 8,055 SNPs removed). A total of 633,852 autosomal SNPs passed all filters (98.38% of the array), 558,832 SNPs of those are included in our callable genome which is the final SNP dataset for genotype comparison. In the sexual chromosomes, we applied similar filtering criteria, with 8,948 SNPs remaining in the X chromosome (54.32% of the initial SNPs) and only 5 SNPs in the Y chromosome (out of the starting 10 SNPs).

For additional nine samples (**S2.2 Table**), we compared our genotype calls with the ones generated on Affymetrix's Genome-Wide Human SNP array 6.0. The SNP array datasets were first converted from their original CEL format to Birdseed using Affymetrix Power Tools. We kept variants with confidence values smaller than 0.1, with 862,017 SNPs remaining out of the initial 934,969 SNPs. We got a VCF file with the filtered variants using BirdseedToVCF.py (downloaded from http://archive.broadinstitute.org/cancer/cga/contest_prepare2). We removed masked, multiallelic, and duplicated SNPs (both duplicates if there is any genotype inconsistency, and keeping one otherwise). Moreover, we removed the SNPs falling out of our callable regions, obtaining a total set of 734,734 SNPs for validation, of which 19,472 SNPs belong to the X chromosome (53.71% of the initial set) and 110 SNPs to the Y chromosome (39.86% of the initial set).

For each sample, we computed the percentage of genotype concordance as the proportion of loci having the same genotype in both sets over the total set of loci under evaluation for the autosomes and the X chromosome. Moreover, we calculated how many heterozygous autosomal alleles in the array are heterozygous in our calling (**S2.2 Table**).

**S2.2 Table. SNP calling validation in a subset of samples based on SNP array comparison.**

| SNP Microarray | Sample | Autosomal genotype sensitivity[a] | ChrX genotype sensitivity[b] | # Heterozygous in array | Heterozygous sensitivity |
|---|---|---|---|---|---|
| **Illumina 650Y array** | JuhoansiSan_HGDP01029 | 99.66 | 99.69 | 135,837 | 99.32 |
| | JuhoansiSan_HGDP01036 | 99.67 | 99.85 | 135,125 | 99.35 |
| | MbutiPygmy_HGDP00456 | 99.63 | 99.2 | 141,864 | 99.32 |
| | MbutiPygmy_HGDP00982 | 99.67 | 99.81 | 144,804 | 99.39 |
| | Yoruba_HGDP00927 | 99.67 | 99.66 | 163,899 | 99.39 |
| | Yoruba_HGDP00936 | 99.67 | 99.87 | 167,046 | 99.39 |
| | Mandenka_HGDP01284 | 99.61 | 98.93 | 166,239 | 99.3 |
| | Mandenka_HGDP01286 | 99.67 | 99.75 | 166,785 | 99.37 |
| | French_HGDP00521 | 99.69 | 99.36 | 168,379 | 99.4 |
| | Sardinian_HGDP00665 | 99.68 | 99.34 | 165,965 | 99.4 |
| | Han_HGDP00778 | 99.69 | 99.58 | 153,120 | 99.43 |
| | Dai_HGDP01307 | 99.69 | 99.66 | 153,057 | 99.4 |
| | **All** | **99.67** | **99.56** | | **99.37** |
| **Affymetrix's Genome-Wide Human SNP array 6.0** | KhomaniSan_A403 | 98.87 | 99.44 | 162,261 | 96.63 |
| | BakaPygmy_A405 | 98.92 | 99.27 | 194,664 | 97.6 |
| | SouthAfricanBantu_A402 | 99 | 98.89 | 205,885 | 97.9 |
| | WestAfricanBantu_A404 | 98.01 | 98.78 | 212,147 | 95.3 |
| | Laal_A409 | 99.07 | 99.53 | 208,892 | 97.98 |
| | EastAfricanBantu_A401 | 98.65 | 98.57 | 211,907 | 97.77 |
| | Toubou_A408 | 98.9 | 99.5 | 204,954 | 97.63 |
| | Saharawi_A406 | 98.4 | 99.4 | 190,208 | 97.14 |
| | Libyan_A407 | 98.47 | 99.63 | 201,825 | 97.35 |
| | **All** | **98.70** | **99.22** | | **97.25** |

[a] Sensitivity relative to 558,832 SNPs in the array for the samples in the HGDP panel, and 715,152 SNPs for the other samples.
[b] in the X chromosome there are 8,948 SNPs and 19,472 SNPs under evaluation in the HGDP samples and the rest respectively.

Given the lower sensitivity observed in the comparison between the genotypes of whole-genome sequencing and the ones obtained from Affymetrix's Genome-Wide Human SNP array 6.0 for 9 samples, we sought to characterize the set of discordant loci. To do that we first downloaded the ancestral alleles that were calculated in the 1,000 Genome Project for GRCh37 (http://ftp.1000genomes.ebi.ac.uk/vol1/ftp/phase1/analysis_results/supporting/ancestral_alignments/). Then we calculated the GC content and transition/transversion ratios for the loci that had

a defined ancestral allele that is one of the two possible genotypes in the array for i) the whole array, ii) the set of discordant loci for each sample, and iii) the set of discordant alleles that are discordant in at least two samples (**S3.2 Table**). Overall, the transition/transversion ratio is much lower in the sets of discordant loci (<=2). This observation indicates that the genotyping is of low quality for these sets of SNPs in the case of the Affymetrix arrays.

**S2.3 Table. Characterization of discordant loci in Affymetrix's Genome-Wide Human SNP array 6.0**

| | AUTOSOMES | | | | | | | |
|---|---|---|---|---|---|---|---|---|
| | **Number of SNPs** | **Number w Anc Allele as REF or ALT** | **% with Anc Allele** | **GC content in ANC** | **%GC content in ancestral allele** | **Ti** | **Tv** | **Ti/Tv** |
| **All Positions** | 715,152 | 702,937 | 98.29 | 380,456 | 54.12 | 485,861 | 217,076 | 2.24 |
| **Discordants** | | | | | | | | |
| **BakaPygmy_A405** | 7,709 | 7,486 | 97.11 | 4,153 | 55.48 | 4,859 | 2,627 | 1.85 |
| **EastAfricanBantu_A401** | 9,667 | 9,407 | 97.31 | 5,050 | 53.68 | 6,247 | 3,160 | 1.98 |
| **KhomaniSan_A403** | 8,109 | 7,877 | 97.14 | 4,312 | 54.74 | 5,186 | 2,691 | 1.93 |
| **Laal_A409** | 6,656 | 6,473 | 97.25 | 3,588 | 55.43 | 4,167 | 2,306 | 1.81 |
| **Libyan_A407** | 10,971 | 10,685 | 97.39 | 5,915 | 55.36 | 7,123 | 3,562 | 2.00 |
| **Saharawi_A406** | 11,473 | 11,178 | 97.43 | 6,020 | 53.86 | 7,383 | 3,795 | 1.95 |
| **SouthAfricanBantu_A402** | 7,122 | 6,892 | 96.77 | 3,813 | 55.33 | 4,530 | 2,362 | 1.92 |
| **Toubou_A408** | 7,858 | 7,626 | 97.05 | 4,220 | 55.34 | 5,007 | 2,619 | 1.91 |
| **WestAfricanBantu_A404** | 14,264 | 13,873 | 97.26 | 7,709 | 55.57 | 9,219 | 4,654 | 1.98 |
| | | | | | | | | |
| **Discordants In More than One sample** | 13,766 | 13,356 | 97.02 | 7,413 | 55.50 | 8,744 | 4,612 | 1.90 |

## S3) Mitochondrial and Y-Chromosome analysis

### Mitochodrial reconstruction

The complete mitochondrial sequences of the 25 samples of this project were reconstructed from the whole-genome shotgun paired-end sequencing. For each sample, we recovered a subset of reads by mapping the whole set of paired-end reads against a high-quality human mitochondrial reference genome [5]. To maximize the number of reads captured at the extremes of the reference assembly, we applied a second round of mapping in which we took advantage of the mitochondrial circularity. We aligned the reads to the reference sequence but, this time, the origin was modified to be at the middle of the assembly (8 kbp from the start). Alignments were performed using the BWA mapper with parameters `-n 6 -q 15` [3]. We retained only high-quality paired-end reads by imposing that both pairs should be mapped and properly paired (with `samtools -f 2` [6]) and by requiring that both pairs have a median Phred quality score greater than 32. In **S3.1 Table** it is shown the final mitochondrial coverage obtained per sample in both read capturing rounds.

**S3.1 Table. Number of captured high-quality reads and mitochondrial coverage per sample.**

| Sample | Standard Assembly | | Modified assembly[a] | |
|---|---|---|---|---|
| | Total Reads | Coverage[b] | Total Reads | Coverage[b] |
| JuhoansiSan_HGDP01029 | 6,947,302 | 39,413.70 | 6,939,132 | 39,367.40 |
| JuhoansiSan_HGDP01036 | 2,908,870 | 16,502.70 | 2,904,310 | 16,476.90 |
| KhomaniSan_A403 | 141,726 | 855.37 | 141,316 | 852.89 |
| TuuSan_KB1 | 74,494 | 341.70 | 74,886 | 343.49 |
| MbutiPygmy_HGDP00456 | 1,976,322 | 11,212.20 | 1,977,312 | 11,217.80 |
| MbutiPygmy_HGDP00982 | 2,281,060 | 12,941.00 | 2,279,086 | 12,929.80 |
| BakaPygmy_A405 | 258,484 | 1,560.05 | 257,906 | 1,556.56 |
| SouthAfricanBantu_A402 | 177,790 | 1,073.03 | 177,464 | 1,071.06 |
| WestAfricanBantu_A404 | 401,810 | 2,425.07 | 401,258 | 2,421.74 |
| Yoruba_HGDP00927 | 2,664,058 | 15,113.90 | 2,664,048 | 15,113.80 |
| Yoruba_HGDP00936 | 2,927,260 | 16,607.10 | 2,926,698 | 16,603.90 |
| Yoruba_NA18507 | 2,306,846 | 13,087.30 | 2,305,206 | 13,078.00 |
| Mandenka_HGDP01284 | 3,172,204 | 17,996.70 | 3,172,448 | 17,998.10 |
| Mandenka_HGDP01286 | 3,659,028 | 20,758.60 | 3,661,024 | 20,769.90 |
| Laal_A409 | 267,090 | 1,628.11 | 267,412 | 1,630.07 |
| Dinka_DNK02 | 211,204 | 1,198.21 | 210,784 | 1,195.83 |
| Dinka_DNK07 | 600,104 | 3,404.54 | 599,456 | 3,400.86 |
| EastAfricanBantu_A401 | 561,584 | 3,389.37 | 560,988 | 3,385.77 |
| Toubou_A408 | 242,436 | 1,477.82 | 241,416 | 1,471.60 |
| Saharawi_A406 | 306,842 | 1,851.90 | 305,132 | 1,841.58 |
| Libyan_A407 | 342,124 | 2,064.84 | 340,296 | 2,053.81 |
| French_HGDP00521 | 3,155,804 | 17,903.60 | 3,153,304 | 17,889.50 |

| | | | | |
|---|---|---|---|---|
| Sardinian_HGDP00665 | 2,511,800 | 14,250.10 | 2,511,762 | 14,249.80 |
| Han_HGDP00778 | 3,055,850 | 17336.6 | 3,056,378 | 17339.6 |
| Dai_HGDP01307 | 2,171,210 | 12317.8 | 2,169,196 | 12306.4 |

Number of captured high-quality reads and mitochondrial coverage per sample
[a] Assembly with the start position at the middle of the reference mitochondrial assembly
[b] Coverage relative to the length of the mitochondrial reference (16,571 bps)

We constructed contigs from the retrieved reads with Hapsembler v.1.1, a haplotype-specific genome assembly toolkit (parameters `-p Illumina -t 4 -d no --PHRED_OFFSET 33 --MIN_CONTIG_SIZE 1000 –EPSILON 0.05`) [7]. Because the efficiency of this assembler decreases with lofty coverage, such as the one obtained for the mitochondrial reconstruction (**S3.1 Table**), we randomly reduced the number of reads per sample to around 350X of mitochondrial coverage (except for the TuuSan sample KB1 for which the resampling was done at 300X). This reduction with its subsequent construction of contigs was applied 20 times per reference assembly (the standard and the one with the modified origin). The rationale behind this iterative process is to compensate the random representation of reads that entails potential problems such as the assembling of existing *numts* into the mitochondrial sequence.

For each of the 40 times we applied the read reduction and posterior de novo assembling, we oriented the resulting contigs via local alignments to the human mitochondrial reference [5] with BLAST [8]. We then joined the oriented contigs using mafft [9] and incorporating Ns in both existing gaps and sites that remain unresolved due to differences in overlapping contigs. In such a way, we have reconstructed 40 mitochondrial assemblies per sample. Finally, we got the consensus sequence, which is the reported mitochondrial sequence per individual.

## Y Chromosome reconstruction

SNP calling was performed as described in **S2 section**. A total of 14,488 SNPs were identified in the whole Y chromosome once multiallelic positions were excluded, with 4,088 SNPs remaining after considering callable positions only (i.e., 4.24 Mbp of the Y chromosome, see also **S2 section**). As the Y chromosome is especially rich in repeats, duplications, and low-quality regions, we restricted our haplogroup reconstruction to nine high-quality regions determined by Wei et al. [10]. These nine segments, that span 8.97 Mbp, are the result of excluding the pseudoautosomal, heterochromatic, X-transposed, and ampliconic segments from the male specific region of the Y chromosome (MSY, 95% of the chromosome's length) [10, 11]. When considering the callable loci within these high-quality regions, we got a final set of 3,259 SNPs in 3.44 Mbp of genomic sequence.

## Haplogroups and phylogenetic analysis

We identified the mitochondrial haplogroup of each sample by locating the individual variants relative to the Reconstructed Sapiens Reference Sequence (RSRS) [5] into the mitochondrial phylogenetic tree from [12] and from subsequent research publications updated in www.phylotree.org (Build 16). We annotated known and novel variants for each individual haplogroup in **S3.2 Table**. Similarly, we assessed Y-chromosome haplogroups with the AMY-tree software [13] (files used: haplogroup tree v2.1, mutation conversion file v2.1, quality control file v2.0 and Y chromosome reference in hg19) (**S3.3 Table**).

### S3.2 Table. Mitochondrial haplogroups.

| Sample | Length | Haplogroup | Variants |
|---|---|---|---|
| Han_HGDP00778 | 16,568 | A5b1b | 152T,195T,235G,247G,663G,769G,825t,961C,965.XC,1018G,1709A,1736G,2758G,2885T,3594C,4104A,4248C,4312C,4316G,4824G,7146A,7256C,7521G,8468C,8563G,8655C,8701A,8794T,9540T,10398A,10664C,10688G,10810T,10873T,10915T,11536T,11914G,13105A,13276A,13506C,13650C,13999T,16126C,16129G,16187C,16189T,16230A,16234T,16278C,16289G,16290T,16311T,16319A,16519T |
| Dai_HGDP01307 | 16,560 | B4a1c4 | 152T,195T,247G,709A,769G,825t,1018G,2758G,2885T,3594C,4104A,4312C,5465C,7146A,7256C,7521G,8281-8289d,8468C,8655C,8701A,9123A,9540T,10238C,10398A,10664C,10688G,10810T,10873T,10915T,11914G,12705C,12904G,13105A,13276A,13506C,13650C,16093C,16129G,16181.1c,16182c,16183c,16214T,16216G,16217C,16223C,16230A,16261T,16278C,16311T |
| French_HGDP00521 | 16,569 | T1a | 146T,152T,195T,247G,709A,769G,825t,1018G,1888A,2758G,2885T,3394C,3594C,4104A,4216C,4312C,4639C,4917G,7146A,7256C,7521G,8468C,8655C,8697A,8701A,9540T,10398A,10463C,10664C,10688G,10810T,10873T,10915T,11251G,11914G,12633a,12705C,13105A,13276A,13368A,13506C,13650C,14905A,15452a,15607G,15928A,16126C,16129G,16162G,16163G,16186T,16187C,16223C,16230A,16278C,16294T,16311T |
| Sardinian_ HGDP00665 | 16,571 | H3u | 73A,93G,146T,152T,195T,247G,769G,825t,1018G,2706A,2758G,2885T,3594C,4104A,4312C,6776C,7028C,7146A,7256C,7521G,8468C,8655C,8701A,9540T,9966A,10398A,10664C,10688G,10810T,10873T,10915T,11719G,11914G,12705C,13105A,13276A,13506C,13650C,14766C,15315T,16129G,16183.1c,16187C,16223C,16230A,16278C,16311T |
| Saharawi_A406 | 16,566 | L3b1b1 | 146T,195T,217C,247G,769G,825t,1018G,2758G,2885T,3450T,3594C,4104A,4312C,5773A,6221C,7146A,7256C,7521G,8468C,8655C,9079G,9449T,10086G,10373A,10664C,10688G,10810T,10915T,11116C,11914G,12757C,13276A,13506C,13650C,13914a,15301A,15311G,15664T,15824G,T15944d,16124C,16129G,16187C,16189T,16230A,16294T,16311T,16362C |
| Libyan_A407 | 16,567 | L2a1c | 247G,385G,825t,960T,2416C,2758G,2789T,2885T,3010A,4312C,5442C,6663G,7146A,7175C,7274T,7771G,8206A,8468C,8655C,9053A,9221G,9377G,10115C,10192T,10664C,10688G,10810T,10915T,11887A,11944C,12693G,13105A,13276A,13506C,13590A,13803G,15301A,15784C,16187C,16189T,16223C,16230 |

| | | | |
|---|---|---|---|
| | | | A,16294T,16309G,16311T,16390A |
| Mandenka_ HGDP01284 | 16,568 | L2c3a | 93G,150T,182T,198T,247G,325T,513A,680C,709A,825t,1442A,2332T,2416C,2589G,2758G,2885T,3200a,4312C,5255T,6521T,7146A,7624a,8206A,8468C,8655C,8733C,9221G,10115C,10664C,10688G,10810T,10915T,11914G,11944C,12236A,13105A,13276A,13506C,13590A,13928c,13958c,15077A,15110A,15217A,15301A,15849T,16093C,16129G,16187C,16189T,16230A,16234T,16311T,16390A,16519T |
| Mandenka_ HGDP01286 | 16,567 | L1b1a | 146T,182T,185t,357G,709A,710C,1438A,1738C,2352C,2768G,3308C,3666A,3693A,4312C,5036G,5046A,5393C,5655C,6548T,6827C,6989G,7055G,7389C,7867T,8248G,10045C,10664C,10915T,11914G,12480T,12519C,13276A,13789C,13879C,13880a,14178C,14203G,14560A,14769G,15115C,16126C,16129G,16145A,16230A,16264T,16270T,16293G |
| Yoruba_ HGDP00927 | 16,567 | L1b1a | 146T,182T,185t,357G,709A,710C,1438A,1738C,2352C,2768G,3308C,3666A,3693A,4312C,5036G,5046A,5393C,5655C,6548T,6827C,6989G,7055G,7389C,7867T,8248G,9374G,9851T,10664C,10915T,11914G,12519C,13276A,13708A,13789C,13880a,14178C,14203G,14560A,14769G,15115C,15511C,16126C,16129G,16230A,16264T,16270T |
| Yoruba_ HGDP00936 | 16,571 | L2a12b | 247G,825t,2416C,2758G,2789T,2885T,3336C,3613T,3918A,4312C,5285G,7146A,7175C,7274T,7771G,8206A,8468C,8655C,9221G,10115C,10664C,10688G,10810T,10915T,11944C,12693G,13105A,13276A,13392C,13506C,13590A,13803G,14566G,15244G,15301A,15629C,15784C,16129G,16187C,16189T,16230A,16286T,16294T,16309G,16311T,16390A |
| Yoruba_ NA18507 | 16,567 | L1b1a3 | 146T,182T,185t,189G,357G,709A,710C,1438A,1738C,2352C,2768G,3308C,3666A,3693A,4312C,5036G,5046A,5393C,5655C,6548T,6827C,6989G,7055G,7389C,7867T,8248G,10664C,10915T,11488G,11914G,12519C,13276A,13789C,13880a,13980A,14178C,14203G,14560A,14769G,15115C,16126C,16129G,16230A,16264T,16270T,16293G |
| Toubou_A408 | 16,570 | M1 | 146T,152T,247G,489C,769G,825t,1018G,2758G,2885T,3594C,4104A,4312C,5250C,5351G,6446A,6680C,7146A,7256C,7521G,8468C,8655C,9530C,10400T,10664C,10688G,10810T,10895G,10915T,11914G,12403T,12950c,13105A,13111C,13276A,13506C,13650C,13711A,14110C,14783C,15043A,15301A,16183c,16187C,16230A,16249C,16399G,16527T |
| Laal_A409 | 16,588 | L3e1c | 146T,150T,152T,189G,195T,200G,247G,769G,825t,1018G,2352C,2758G,2885T,3594C,3675G,4104A,4312C,5460A,6221C,6587T,7146A,7256C,7521G,8289.1cccccctctaccccctcta,8468C,8655C,8701A,10664C,10688G,10810T,10819G,10915T,11914G,13105A,13276A,13506C,13650C,14152G,14212C,14323A,15301A,15670C,15942C,16129G,16187C,16189T,16223C,16230A,16278C,16311T,16327T,16519T |
| EastAfricanBantu_ A401 | 16,569 | L2a1h | 143A,247G,825t,2416C,2758G,2789T,2885T,3505G,4312C,4772C,7146A,7175C,7274T,7771G,8206A,8468C,8655C,9221G,10115C,10664C,10688G,10810T,10915T,11944C,12693G,12976T,13105A,13276A,13506C,13590A,13764T,13803G,13824G,14566G,15301A,15784C,16129G,16187C,16189T,16230A,16234T,16249C,16294T,16295T,16311T,16390A,16519T |
| WestAfricanBantu_ A404 | 16,567 | L3d3a1 | 146T,195T,247G,769G,825t,921C,1018G,1719A,2758G,2885T,3498g,3594C,4104A,4312C,4688C,5147A,7146A,7256C,7424G,7521G,8251A,8468C,8618C,8655C,10664C,10688G,10810T,10899G,10915T,11404G,11914G,13104G,13276A,13506C,13650 |

| | | | |
|---|---|---|---|
| | | | C,13886C,14284T,15061G,15208G,15301A,15700T,16124C,16129G,16183C,16187C,16230A,16304C,16519T |
| SouthAfricanBantu_A402 | 16,570 | L2a1f | 247G,825t,2416C,2758G,2789T,2885T,4312C,5581G,7146A,7175C,7274T,7771G,8206A,8468C,8655C,9221G,10115C,10664C,10688G,10810T,10915T,11944C,12693G,13105A,13276A,13506C,13590A,13803G,14566G,15301A,15784C,16129G,16187C,16191.1T,16192T,16230A,16294T,16309G,16311T,16390A |
| Dinka_DNK02 | 16,567 | L2c1 | 93G,95c,150T,182T,198T,247G,325T,680C,709A,825t,1442A,2332T,2416C,2758G,2885T,3200a,3745A,4312C,7146A,7159C,7624a,8206A,8468C,8655C,9221G,10115C,10664C,10688G,10810T,10915T,11908G,11914G,11944C,12236A,13105A,13276A,13506C,13590A,13928c,13958c,15110A,15217A,15301A,15849T,16129G,16187C,16189T,16230A,16311T,16318G,16390A,16519T |
| Dinka_DNK07 | 16,567 | L0a1a | 64T,73A,93G,146T,185A,189G,195T,200G,236C,1048T,2245G,3516a,3866C,4586C,5096C,5231A,5442C,5460A,5603T,6185C,8302G,8428T,8566G,9042T,9347G,9755A,9818T,10589A,11176A,11641G,12007A,12720G,14308C,15136T,15431A,16104T,16148T,16168T,16172C,16188g,16278C,16320T,16519T |
| BakaPygmy_A405 | 16,566 | L1c1a2b | 93G,95c,146T,152T,182T,186a,189c,236C,297G,316A,A2395d,2755G,2863C,3513T,3666A,3796t,3843G,3927G,4312C,4454a,4506G,5951G,6071C,6752G,7055G,7202G,7389C,7660C,7693T,8027A,8087C,9072G,9272T,9647C,10321C,10586A,10664C,10915T,11899C,11914G,12768G,12810G,13276A,13485G,13789C,14000a,14088C,14148G,14178C,14560A,14911T,16230A,16274A,16293G,16294T,16360T |
| MbutiPygmy_HGDP00456 | 16,561 | L0a2b | 64T,73A,93G,146T,152T,195T,236C,1048T,2245G,3372C,3516a,4586C,5147A,5231A,5237A,5442C,5460A,5603T,5711G,6185C,6257A,8281-8289d,8428T,8460G,8566G,9042T,9347G,9755A,9818T,10589A,11172G,11176A,11269T,11641G,12007A,12172G,12720G,13281C,14308C,15136T,15431A,16129G,16148T,16172C,16188a,16242T,16278C,16320T,16390A |
| MbutiPygmy_HGDP00982 | 16,563 | L0a2b | 64T,73A,93G,146T,152T,195T,236C,567.1C,1048T,2245G,3372C,3516a,4586C,5147A,5231A,5237A,5442C,5460A,5603T,5711G,6185C,6257A,8281-8289d,8428T,8460G,8566G,9042T,9347G,9755A,9818T,10589A,11172G,11176A,11269T,11641G,12007A,12172G,12720G,13281C,14308C,15136T,15431A,16129G,16148T,16172C,16188a,16242T,16278C,16320T,16390A |
| JuhoansiSan_HGDP01029 | 16,570 | L0d1b1 | 263A,C498d,567.1c,567.2c,567.3c,719A,1048T,1438A,2706A,3438A,3516a,3618C,3756G,4232C,5029C,5442C,6185C,6266G,6815C,7283C,8113a,8152A,8251A,8383C,8937C,9042T,9347G,9755A,10589A,12007A,12121C,12720G,13759A,14315T,14659T,15466A,15692G,15930A,15941C,16230A,16239T,16243C,16278C,16294T |
| JuhoansiSan_HGDP01036 | 16,566 | L0d1c1a | 198T,263A,456T,C498d,593C,719A,1048T,1438A,2706A,3438A,3516a,3666A,3756G,4197T,4232C,5442C,6185C,6266G,6815C,7828G,8113a,8152A,8251A,9042T,9150G,9347G,9438A,9755A,10589A,11437C,12007A,12121C,12235C,12720G,13129T,13759A,15466A,15550T,15930A,15941C,15951G,16129G,16167T,16234T,16242T,16243C,16278C |
| KhomaniSan_A403 | 16,567 | L0d2a1 | 152T,198T,263A,C498d,597T,1048T,1438A,3516a,3756G,3981G,4025T,4044G,4225G,4232C,5153G,5441G,5442C,6185C,6815C,7154G,8113a,8152A,8251A,8392A,8545A,9042T,9347G,97 |

| | | | |
|---|---|---|---|
| | | | 55A,10589A,11854C,12007A,12121C,12172G,12234G,12720G,12810G,12894G,14221C,15466A,15766G,15930A,15941C,16212G,16243C,16278C,16320T,16390A |
| TuuSan_KB1 | 16,568 | L0d1b2 | 263A,C498d,499A,593C,719A,1048T,1438A,2706A,3438A,3516a,3618C,3756G,4232C,5442C,6185C,6266G,6692G,6815C,7283C,8113a,8152A,8251A,8790A,9042T,9347G,9755A,9887C,10589A,12007A,12121C,12720G,13759A,14280G,14315T,14560A,14659T,15449C,15466A,15930A,15941C,16153A,16243C,16278C,16294T,16474t |

For each sample, the mitochondrial sequence length and haplogroup are shown. Coordinates of variants are also given, relative to RSRS [5]. In lower case, transversions; in upper case, transitions. Deletions are indicated as the deleted nucleotide and the position followed by a "d"; insertions are labeled with a dot after the position number and type of inserted nucleotide(s). Polynucleotide stretches of unknown length are indicated as the position, a dot, an "X" and the nucleotide. Variants in the D-loop are shown in green. Novel variants are underlined. Insertions of 1 to 3 Cs at position 309, dinucleotide insertion AC at position 523-524 and position 3,107 have been excluded.

### S3.3 Table. Y-chromosome haplogroups.

| Samples | # variants | # singletons | Haplogroup (in phylotree database) | Haplogroup (in ISOGG database) |
|---|---|---|---|---|
| JuhoansiSan_HGDP01029 | 764 | 352 | A2a1a | A1b1a1a1 |
| JuhoansiSan_HGDP01036 | 772 | 50 | A3b1c | A1b1b2a |
| KhomaniSan_A403 | 778 | 51 | A3b1 | A1b1b2a |
| TuuSan_KB1 | 503 | 217 | B2b1b1 | B2b1b |
| MbutiPygmy_HGDP00456 | 374 | 17 | E1b1a1a1f1a1d | E1b1a1a1c1a1c |
| MbutiPygmy_HGDP00982 | 356 | 102 | E2b1a | E2b1a1 |
| BakaPygmy_A405 | 366 | 11 | E1b1a1a1f1a1d | E1b1a1a1c1a1c |
| SouthAfricanBantu_A402 | 382 | 29 | E1b1a1a1g1c | E1b1a1a1d1c |
| WestAfricanBantu_A404 | 373 | 13 | E1b1a1a1f1a1d | E1b1a1a1c1a1c |
| Yoruba_HGDP00927 | 364 | 12 | E1b1a1a1f1a1 | E1b1a1a1c1a1 |
| Yoruba_HGDP00936 | 367 | 16 | E1b1a1a1f1a1 | E1b1a1a1c1a1 |
| Yoruba_NA18507 | 362 | 11 | E1b1a1a1f1a1 | E1b1a1a1c1a1 |
| Mandenka_HGDP01284 | 358 | 130 | E1a1 | E1a1 |
| Mandenka_HGDP01286 | 381 | 71 | E1b1b1a1a | E1b1b1a1a1 |
| Laal_A409 | 501 | 222 | B1c | B1 |
| Dinka_DNK02 | 377 | 120 | E2a | E2a |
| Dinka_DNK07 | 777 | 164 | A3b2f | A1b1b2b |
| EastAfricanBantu_A401 | 382 | 18 | E1b1a1a1f1a1d | E1b1a1a1c1a1c |
| Toubou_A408 | 249 | 122 | T1a1 | T1a1 |
| Saharawi_A406 | 377 | 3 | E1b1b1b1 | E1b1b1b1a |
| Libyan_A407 | 381 | 8 | E1b1b1b1 | E1b1b1b1a |
| French_HGDP00521 | 279 | 86 | I1a | I1a |
| Sardinian_HGDP00665 | 266 | 66 | I2a1a1 | I2a1a1 |
| Han_HGDP00778 | 256 | 87 | O2a2c2c | O3a2c1a |
| Dai_HGDP01307 | 262 | 91 | O1b1 | O2 |

For each sample, number of variants and singletons evaluated to determine the Y-chromosome haplogroup are shown, as well as the identified haplogroups (given in both phylotree and ISOGG v.11.01 nomenclature).

We built a neighbor-joining phylogenetic tree (MEGA5.2.2, Model/Method: p-distance, Gaps/Missing data treatment: pairwise deletion) from both the mitochondrial and the Y-chromosome sequences (**S3.1 Fig.**).

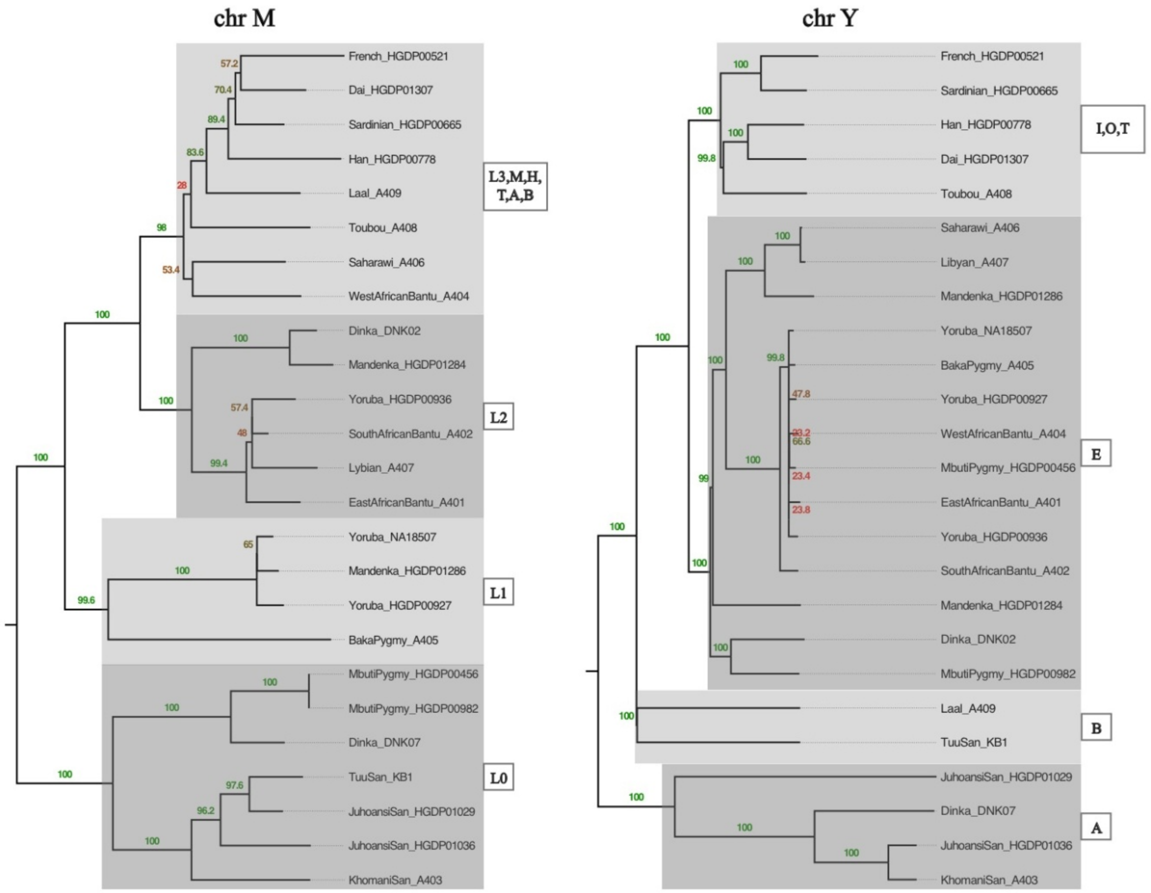


**S3.1 Fig. Mitochondrial and Y-chromosome trees.** Haplogroups and bootstrap confidence values with 1,000 repetitions are shown.

## S4) Genetic diversity and runs of homozygosity

### Pairwise differences

For each sample, we created haploid autosomal chromosomes by randomly choosing one of the two possible genotypes at any given locus. Then, we compared any two distinct individuals by counting the number of different alleles between their sampled genomes (**Figure 1b**). This is a measure of genetic diversity within and between populations, taken as an estimate of the heterozygosity within a population if both samples belong to same population, or between populations otherwise. Similarly, we calculated the heterozygosity on each sample (**Figure 1b**). Moreover, we calculated jackknife estimates and standard errors by dividing the genome into 100 blocks of equal number of SNPs (**S4.1 Fig.**).

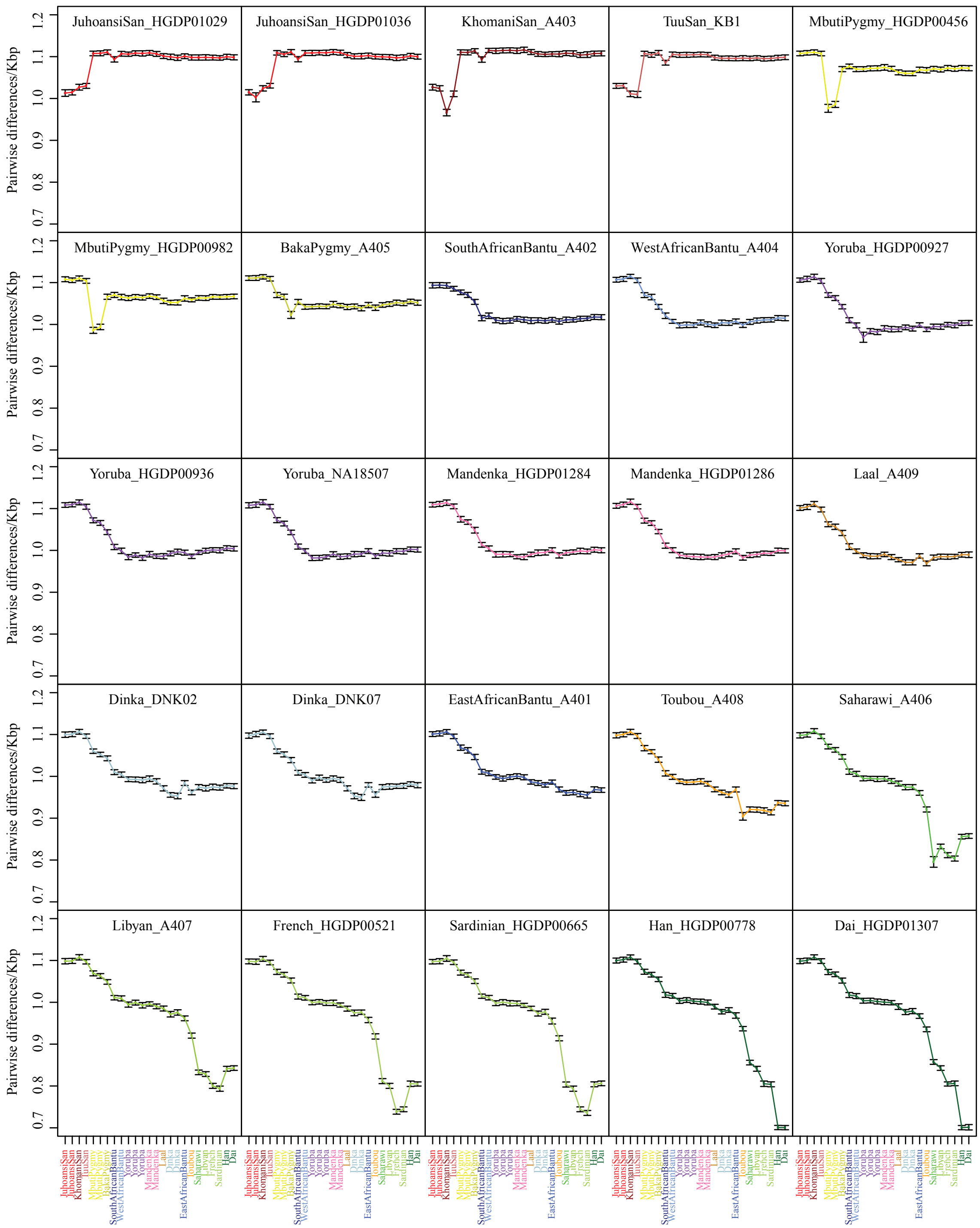


**S4.1 Fig. Pairwise differences per kbp.** For each sample Jackknife estimates and standard errors of the pairwise differences with any other sample is shown.

## Runs of homozygosity (ROH)

For each sample, we considered windows of 1 kbp of callable genome and counted the number of heterozygous alleles included in each window. To determine long homozygous regions, or runs of homozygosity, we identified consecutive windows having less than a given number of heterozygous sites by sliding over 100 windows. We imposed three different minimum lengths for calling ROH: 0.5, 1 and 1.5 Mbp of callable genome, which corresponds to imposing 500, 1,000 and 1,500 consecutive windows. We allowed a maximum of 50, 100, and 150 heterozygous loci, respectively. Assuming an average heterozygosity of 1 per kbp, we are calling a ROH when there is less than approximately 10% of the expected heterozygosity. To ensure that a long run of homozygosity is not due to regions of lower quality when calling SNPs, we imposed that at least 67% of a ROH should be callable. This threshold is equivalent to the proportion of the callable genome in the whole genome (**Figure 1b** and **S4.2 Fig.**).

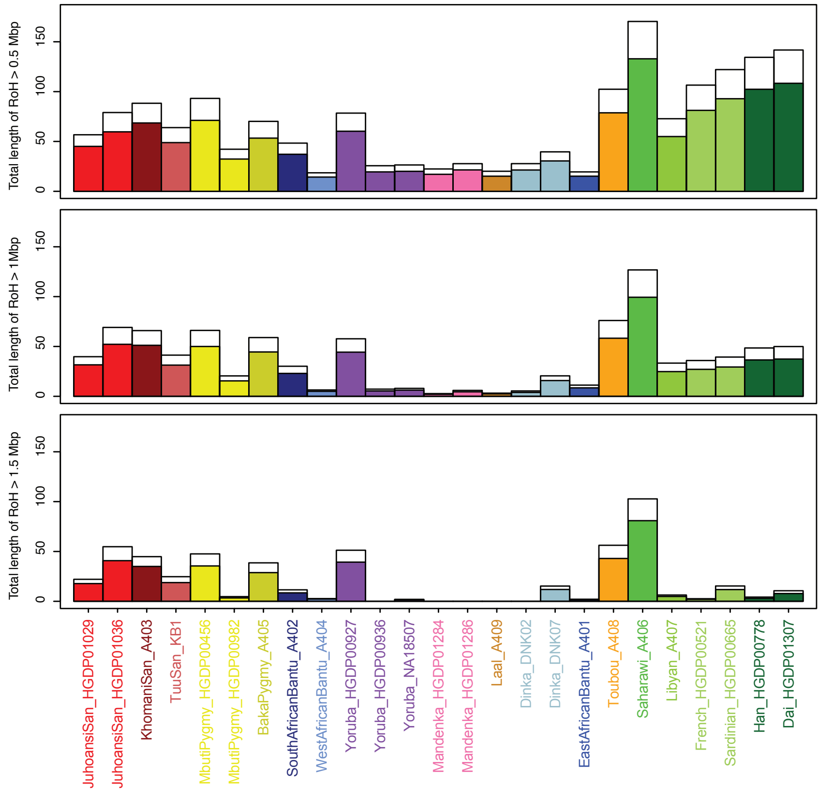


**S4.2 Fig. Total length of runs of homozygosity per individual.** Three panels are shown, from top to bottom: considering 500 windows of 1 kbp of callable genome; considering 1,000 windows; and considering 1,500 windows. The upper rectangles in white represent the proportion of non-callable genome within the ROH.

## S5) Spatial analyses

To investigate the relationship between genetic variation and geographic sampling locations in the African samples, we assessed three different estimates: i) the correlation between geographic and genetic based maps, ii) the genetic differentiation relative to geographic distances, and, iii) the direction in space of this genetic differentiation.

We first conducted a multidimensional scaling (MDS) analysis with an Identical By State (IBS) distance matrix between all the African individuals using SNPs with a MAF > 0.05 and in linkage equilibrium. LD pruning was performed with Plink [14] using the option *--indep* with default parameters (*50 5 2)*. We then quantified the correlation between the geographic map and the genetic relationship between individuals using the first two MDS dimensions by means of a Procrustes analysis [15]. Secondly, we computed a Mantel correlogram [16] in PASSAGE 2.0 [17] using the IBS distance matrix between each pair of individuals and considering five equidistant categories. Lastly, we estimated the patterns of anisotropy - whether the spatial pattern of the genetic diversity differs along cardinal axes – by means of the Bearing procedure algorithm [18] as implemented in PASSAGE 2.0 [17]. In this analysis, a Mantel correlogram is estimated between the matrix of IBS genetic distances and a weight matrix W($\theta$) which is estimated for each pair of points *i,j* as:

$$w(\theta)_{ij} = \Delta_{ij}(\cos(\alpha_{ij} - \theta))^2$$

where $\Delta_{ij}$ and $\alpha_{ij}$ are the geodesic distance and angle between *i,j*, respectively, and $\theta$ is the angle of anisotropy. $\theta$ is estimated by the Bearing procedure by iteratively increasing $\theta$ one degree, computing a new W, then estimating the correlation between the IBS genetic distance matrix and W($\theta$).

Procrustes analysis using the individual coordinates from the first two MDS dimensions and the geographic sampling locations of the individuals reported a statistically significant correlation of R=0.577 (p-value based on 1000 replications = 0.003). This result suggests that genetics tend to fit the geographic sampling location of the sampled individuals (**S5.1 Fig.**).

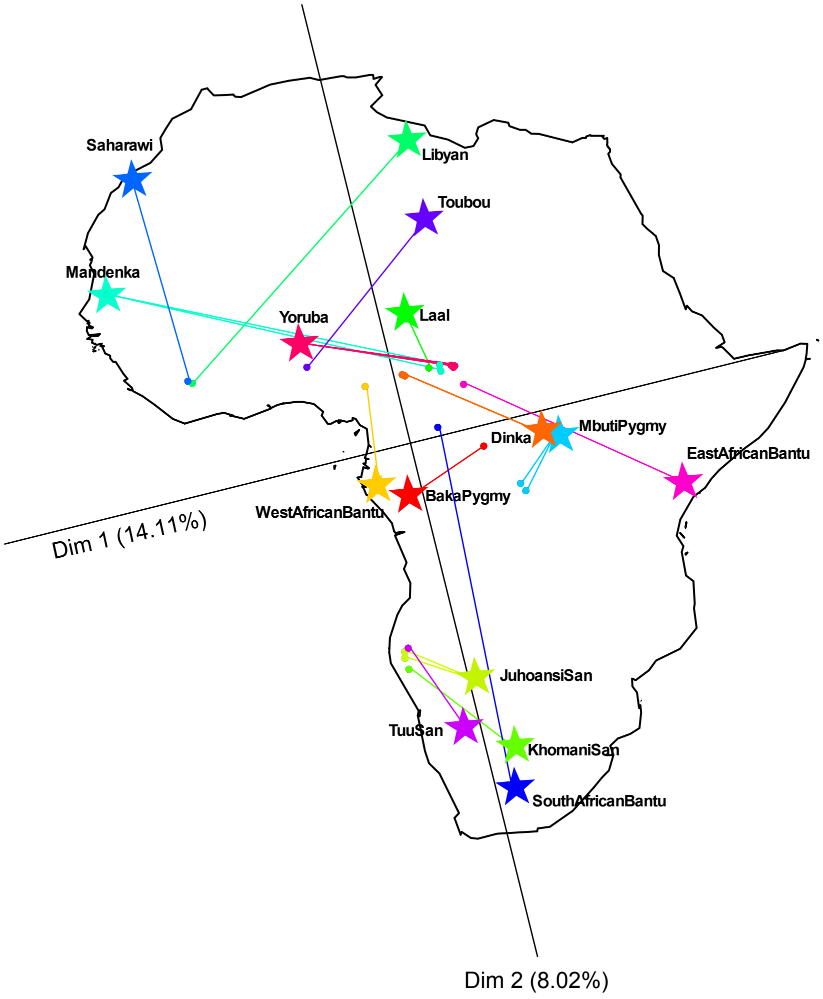


**S5.1 Fig. Geographic and genetic maps of African samples.** Map of Africa with the geographic sampled locations indicated as stars. Dots represent the position of the samples in the first two dimensions of a MDS analysis (based on IBS matrix) that have been imposed after rotation and translation to the geographic locations by means of a Procrustes analysis. Lines indicate the difference between the position in the MDS and the geographic sampling location after Procrustes rotation.

Given the observed dependence between geography and genetics, we aimed to identify whether the genetic variability observed among African individuals followed a particular spatial pattern. Mantel correlogram analysis (**S5.2 Fig.**) suggests that genetic differentiation tends to increase monotonically with geographic distance, a pattern consistent with a main genetic gradient among African populations.

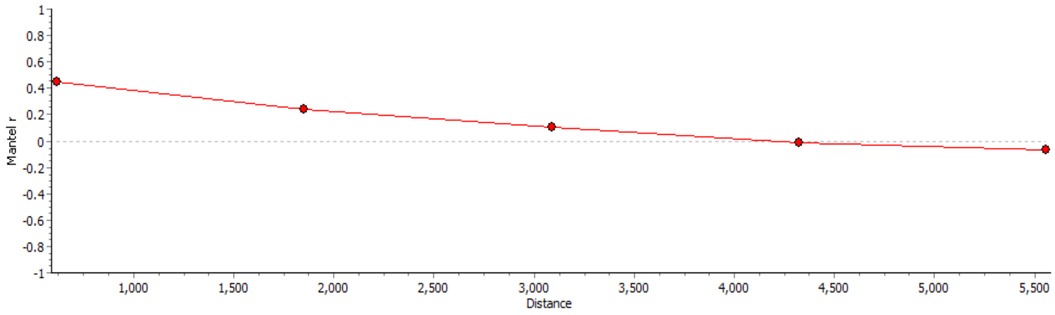


**S5.2 Fig. Mantel correlogram between the genetic distances between pairs of individuals estimated by means of IBS and the geographic distance estimated by Harversine great circle geographic distance.** Lower correlations indicate higher genetic differentiation.

Finally, given the observed genetic pattern, we investigated which was the main angle of anisotropy present in the data. The Bearing procedure indicates in general terms the spatial direction of the greatest correlation between large geographic distances and large distances in the data. The direction of maximal correlation indicates the most likely direction of a gradient. Therefore, the Bearing procedure on our data (**S5.3 Fig.)** supports that the direction in space of maximum genetic differentiation in the African continent is in the North-West to South-East axis (~160 degrees).

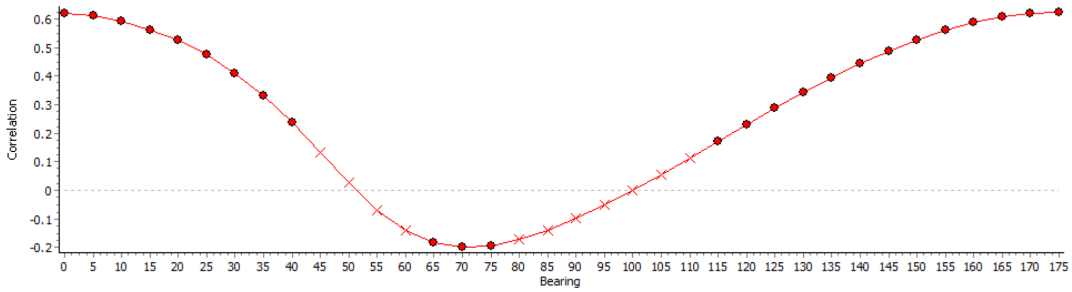


**S5.3 Fig. Bearing plot using the IBS genetic distance between pair of individuals.** Red dots indicate statistically significant correlation values after Bonferroni correction.

## S6) Genetic structure and admixture tests

### Principal Component Analysis (PCA)

We took into consideration the genotypes of the autosomal SNPs not fixed for the alternative allele (11,912,368 SNPs) and performed a principal component analysis (PCA) with the function `prcomp` from R. Variables were centered but not scaled in order to avoid an up-weighted contribution of rare variants, and divided by the square-root of the number of SNPs in order to correct projections on the principal components (PCs) for the influence of an elevated number of variables [19] (**S6.1 Fig.**).

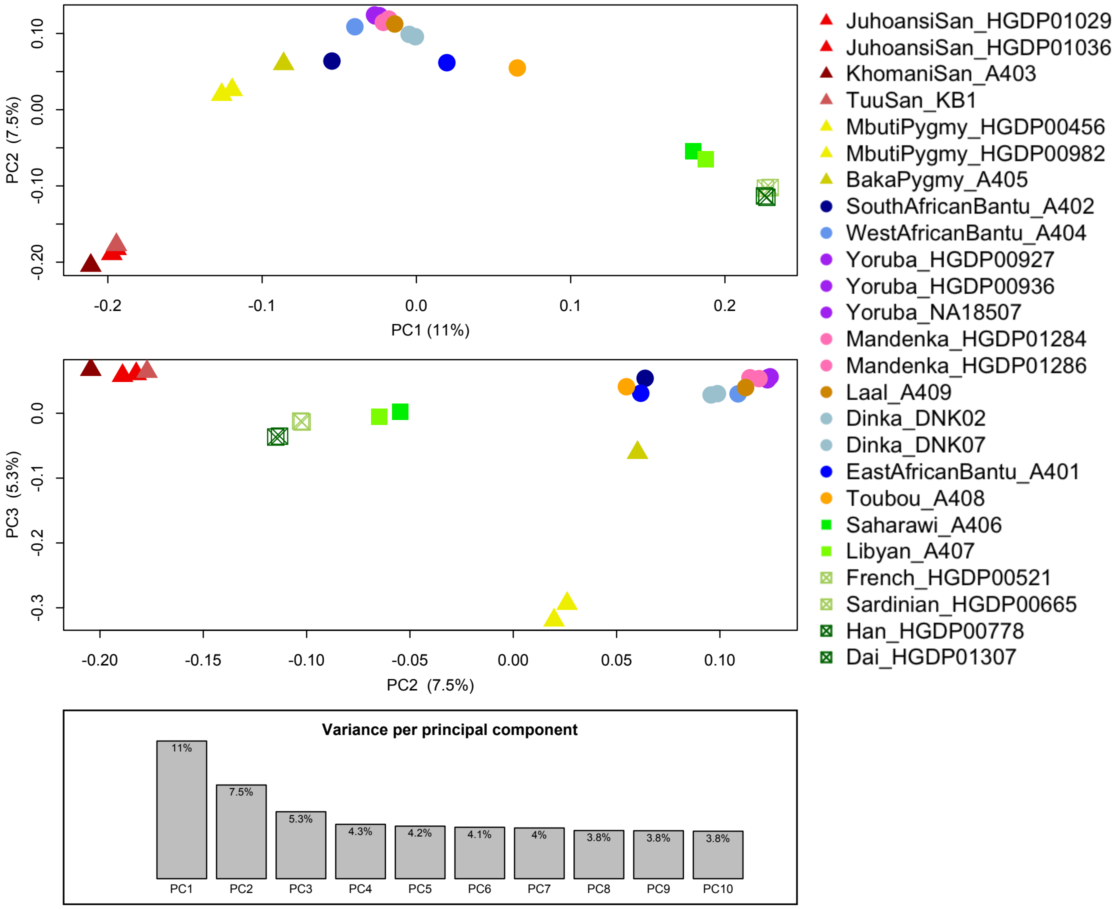


**S6.1 Fig. Principal Component Analysis of 25 samples using 11,912,368 SNPs.** Three panels are shown, from top to bottom: first and second PCs; second and third PCs; and eigenvalues of the first 10 PCs.

## ADMIXTURE

We inferred population structure present in our data with ADMIXTURE [20]. In order to have more representative samples per population, we downloaded the 'Bushman' data library from Galaxy [21], consisting of 419,969 SNPs from 1,462 samples [22]. We took into consideration for our analysis 376,258 SNPs that are part of the callable genome (see **Section S2**) and that have consistent alleles in the two datasets (only 12 SNPs were excluded because of unmatching alleles). For fourteen samples that were present in both studies, we included only our genotypes, resulting in 1,473 individuals. We excluded SNPs having a minor-allele frequency lower than 0.01 when considering the 1,473 samples, resulting in a final set of 374,195 SNPs. Finally, we selected specific populations (all African and Europeans, and only a subset of Asian populations. Then we run ADMIXTURE for K=3 to 10 considering the 745 individuals that belong to the selected populations, and identified K=7 as the best value for parameter K on the basis of the cross-validation error (**S6.2 Fig.**). We then plotted our 25 samples (**Fig 2b**) separately from the 720 remaining individuals (**S6.3 Fig.**).

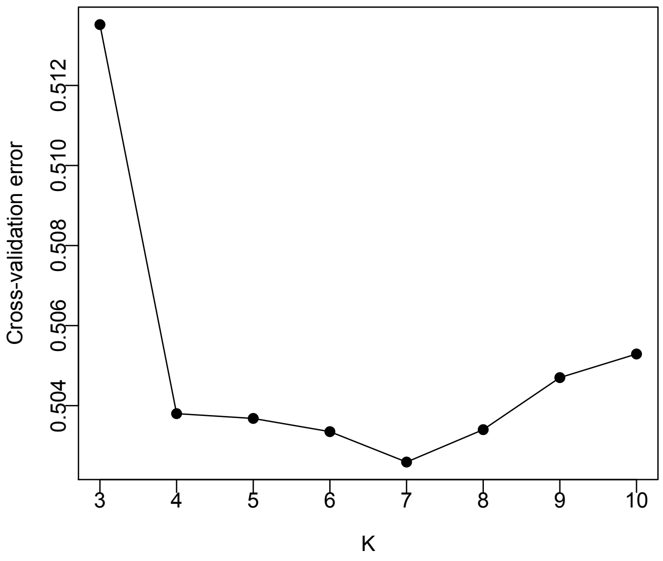


**S6.2 Fig. Cross validation plot to identify the best value for K when using ADMIXTURE.**

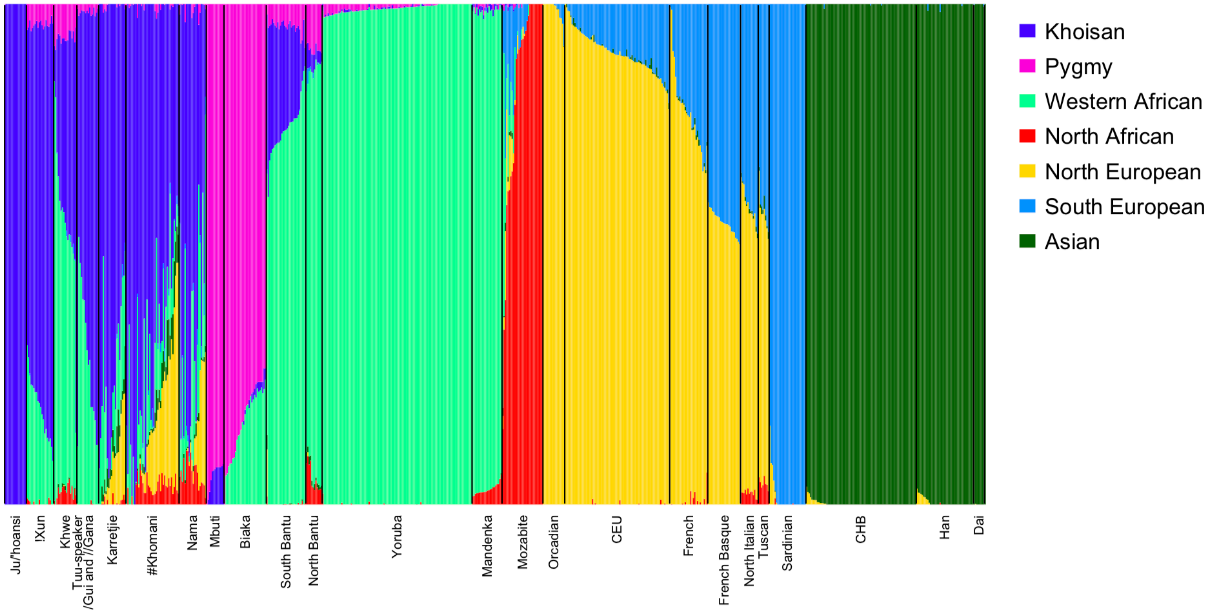


**S6.3 Fig. Population structure of 720 individuals from the Bushman dataset** [22], inferred by ADMIXTURE from 374,195 SNPs.

## D-statistics and $F_4$-ratio estimation

We formally evaluated whether admixture occurred between hunter-gatherer groups and their geographical close populations, as well as from Eurasian to African populations, with the D-statistics test (also called ABBA-BABA test) [23]. We performed the analyses using the ADMIXTOOLS 4.1 software package applying default parameters [23]. In short, given a correct phylogeny that includes four samples (((P1, P2), REF), OUT), D-statistics tests for gene flow from population REF into P1 or P2, being OUT an outgroup assumed to have not admixed with any of the other three. Let A and B be the ancestral and derived alleles on a given locus, respectively, ABBA sites are those where P2 and REF share the derived allele whereas P1 carries the ancestral allele. BABA sites in turn are those where P1 and REF share the derived allele whereas P2 carries the ancestral allele. The test measures the relative excess of BABA sites over ABBA sites. Equal numbers of ABBA and BABA sites are expected under the null hypothesis of no differential levels of gene flow between REF/P1 and REF/P2, which is translated as $D = 0$. Gene flow between REF and P1 is indicated by $D > 0$, while between REF and P2 by $D < 0$, respectively. The statistical significance of the D-statistics is assessed with a weighted block jackknife considering a block size of 5 cM [24]; this is, the D-statistics is computed for 5 Mbp non-overlapping sliding windows across the genome, and the resulting

values are used to compute the weighted mean, variance, and standard error of the statistic over the entire genome. By dividing the average D value by the standard error, a Z-score is obtained. Results are considered significant when |Z| > 3.

Additionally, we determined the proportion of west European ancestry into African populations by constructing a $F_4$-ratio estimate (see below). This method estimates the ancestry proportion of a mixing population under certain correct phylogeny (see Patterson et al. [23] or Pickrell et al. [25] for a longer introduction). Similar to the D-statistics tests, Z-scores were calculated following the same weighted block jackknife strategy. All analyses were also performed using ADMIXTOOLS 4.1 [23].

**1. Admixture between hunter-gatherers and their surrounding populations**

Assuming the split between the ancestors of current hunter-gatherer and farmer populations occurred before the distinct farmer groups split, significantly positive scores resulting from the test D(X=Surrounding population, Y=Non-surrounding population, Z=Hunter-gatherer, Chimpanzee) are taken as evidence of gene flow between the hunter-gatherer group and its corresponding close population under evaluation. These populations are South African Bantu in the case of the Khoisan; Laal, Dinka, Toubou, and Eastern Bantu in the case of the Mbuti Pygmies; and Western Bantu and Yoruba in the case of the Baka Pygmy. Chimpanzee alleles for our variant positions were retrieved from the alignments of the chimpanzee assembly against hg19 downloaded from http://hgdownload.cse.ucsc.edu/goldenPath/hg19/vsPanTro4/reciprocalBest. Results of the tests for Khoisan populations are shown in **S6.1 Table**.

**S6.1 Table. D-statistics results evaluating Khoisan surrounding populations**

| | JuhoansiSan | | KhomaniSan | | TuuSan | |
|---|---|---|---|---|---|---|
| | D | Z | D | Z | D | Z |
| WestAfricanBantu | 0.024 | 7.134* | 0.038 | 9.049* | 0.035 | 8.485* |
| Yoruba | 0.026 | 8.552* | 0.038 | 10.033* | 0.033 | 8.896* |
| Mandenka | 0.027 | 8.949* | 0.039 | 9.83* | 0.034 | 8.638* |
| Laal | 0.016 | 4.746* | 0.033 | 7.796* | 0.022 | 5.386* |
| Dinka | 0.010 | 3.389* | 0.023 | 5.554* | 0.019 | 4.648* |
| EastAfricanBantu | 0.013 | 3.729* | 0.022 | 5.110* | 0.017 | 3.901* |
| Toubou | 0.008 | 2.182 | 0.021 | 4.946* | 0.018 | 3.978* |
| Saharawi | 0.006 | 1.602 | 0.024 | 5.189* | 0.015 | 3.314* |
| Libyan | 0.005 | 1.309 | 0.022 | 4.944* | 0.017 | 3.744* |

D-statistics applied: D(SouthAfricanBantu, Y=Non-surrounding population; Z=Khoisan, Chimpanzee). Y are the populations in the first column. Significant results (|Z| > 3) are marked with an asterisk.

A highly significant amount of shared alleles between Khoisans and the South African Bantu individual is observed. Because these ancestral alleles might be shared with Pygmies and could distort the results, we did not use the Southern Bantu as a non-surrounding population when testing admixture between Pygmies and their neighboring populations. Results of the tests for Mbuti and Baka Pygmy populations are shown in **S6.2-3 Tables**.

**S6.2 Table** shows Dinka, Laal, and Toubou as the groups that share more derived alleles with Mbuti when compared to the rest. Moreover, the closer the non-East African groups (i.e., rows) are to Mbuti, the smaller the value of D (for all four East African groups), which could be partly explained by the Bantu expansion from West to East Africa and its contribution to ancestry of Mbuti.

**S6.2 Table. D-statistics results evaluating Mbuti Pygmy surrounding populations**

| | Laal | | Dinka | | Toubou | | EastAfricanBantu | |
|---|---|---|---|---|---|---|---|---|
| | D | Z | D | Z | D | Z | D | Z |
| WestAfricanBantu | 0.013 | 4.563* | 0.019 | 7.517* | 0.009 | 3.047* | 0.004 | 1.513 |
| Yoruba | 0.014 | 5.388* | 0.020 | 9.528* | 0.010 | 3.576* | 0.005 | 1.975 |
| Mandenka | 0.017 | 6.095* | 0.023 | 10.239* | 0.013 | 4.594* | 0.008 | 2.937 |
| Laal | | | 0.007 | 2.411 | -0.004 | -1.306 | -0.009 | -3.091* |
| Dinka | -0.007 | -2.411 | | | -0.011 | -3.775* | -0.016 | -6.139* |
| EastAfricanBantu | 0.009 | 3.091* | 0.016 | 6.139* | 0.005 | 1.529 | | |
| Toubou | 0.004 | 1.306 | 0.011 | 3.775* | | | -0.005 | -1.529 |
| Saharawi | 0.010 | 3.074* | 0.017 | 6.198* | 0.007 | 1.939 | 0.001 | 0.391 |
| Libyan | 0.008 | 2.350 | 0.015 | 5.242* | 0.004 | 1.135 | -0.001 | -0.373 |

D-statistics applied: D(X, Y; Mbuti Pygmy, Chimpanzee). X are the populations in the top row and Y are the populations in the first column. Significant results (|Z| > 3) are marked with an asterisk.

**S6.3 Table. D-statistics results evaluating Baka Pygmy surrounding populations**

| | WestAfricanBantu | | Yoruba | |
|---|---|---|---|---|
| | D | Z | D | Z |
| WestAfricanBantu | | | -0.002 | -0.636 |
| Yoruba | 0.002 | 0.636 | | |
| Mandenka | 0.008 | 2.344 | 0.006 | 2.475 |
| Laal | 0.001 | 0.139 | -0.002 | -0.465 |
| Dinka | -0.002 | -0.441 | -0.004 | -1.353 |
| EastAfricanBantu | 0.007 | 1.744 | 0.005 | 1.557 |
| Toubou | -0.005 | -1.247 | -0.007 | -2.292 |

| | | | | |
|---|---|---|---|---|
| **Saharawi** | 0.006 | 1.416 | 0.004 | 1.154 |
| **Libyan** | 0.009 | 2.071 | 0.007 | 2.105 |

D-statistics applied: D(X, Y; Baka Pygmy, Chimpanzee). X are the populations in the top row and Y are the populations in the first column. There are no significant results (|Z| > 3).

## 2. Gene flow from West Eurasians to African populations

Expected absence of direct gene flow between East Asia and any African population allows us to properly consider the statistic D(Han, French; X, Chimpanzee) for testing admixture between European (represented by the French sample) and African populations (the X population under evaluation). Negative results with an absolute Z-score higher than 3 are taken as evidence of gene flow between the French individual and the African population under evaluation. Test results are displayed in **S6.4 Table**. The fact that D values are negative for all African samples (although non-significant in most cases) might be partly explained due to an excess of ancestral alleles in the Han in comparison to European/African samples, coming from a more divergent lineage (such as Neanderthal or Denisova, see **S8 section**).

**S6.4 Table. D-statistics results evaluating European gene flow with African populations**

| | D | Z |
|---|---|---|
| **JuhoansiSan** | -0.0111 | -2.944 |
| **KhomaniSan** | -0.0118 | -2.874 |
| **TuuSan** | -0.0084 | -1.984 |
| **MbutiPygmy** | -0.0035 | -0.953 |
| **BakaPygmy** | -0.0094 | -2.073 |
| **SouthAfricanBantu** | -0.0114 | -2.474 |
| **WestAfricanBantu** | -0.0126 | -2.745 |
| **Yoruba** | -0.0111 | -2.923 |
| **Mandenka** | -0.0118 | -2.959 |
| **Laal** | -0.0123 | -2.555 |
| **Dinka** | -0.011 | -2.69 |
| **EastAfricanBantu** | -0.024 | -5.132* |
| **Toubou** | -0.0378 | -7.533* |
| **Saharawi** | -0.0807 | -15.299* |
| **Libyan** | -0.0734 | -13.812* |

D-statistics applied: D(Han, French; X, Chimpanzee), being X any African sample (represented in rows). Significant results (|Z| > 3) are marked with an asterisk.

To infer the mixing proportion of west European ancestry present in the African populations, we constructed a $F_4$-ratio statistic [23] adapted to our data but following similar rationale to the one used in Pickrell et al. [25] when calculating west European ancestry in African populations.

When testing hunter-gatherers, we used f4(Han, Yoruba; X, Chimp) / f4(Han, Yoruba; French, Chimp) whereas f4(Sardinian, Han; X, Yoruba) / f4(Sardinian, Han; French, Yoruba) was used for the rest of African groups. In both cases X is the target African population. This method requires a correct phylogeny (**S6.7 Fig.**) and an unadmixed population. Since west Eurasian genetic signal in west African populations, particularly in Yoruba, has been claimed to be low [25] or even undetectable [26], we chose this population as a proxy for an unadmixed pool. For interrogating agriculturalist populations, the Sardinian sample is the only one in our dataset that allows us to construct a proper phylogeny for these tests. In theory, Sardinians are not the ideal candidate population since they are also west Eurasian. Nevertheless, we expect the test to take advantage of the fact that Sardinians are highly similar to Neolithic samples [27], which opposes to the west Eurasian ancestry expected to be present in African populations. Results for the $F_4$-ratio are shown in **S6.5 Table**.

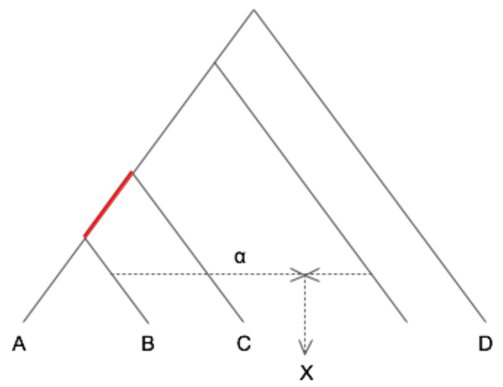


**S6.7 Fig. Population phylogeny used for F4-ratio estimation.** It is applied to two different cases: when the admixed X population is a hunter-gatherer (whose split from the global tree took place before the split of Yoruba), A represents the Han, B represents the French, C represents the Yoruba and D represents the Chimpanzee; whereas when the admixed X population is any other African non-hunter-gatherer population, A represents the Sardinian, B represents the French, C represents the Han and D represents the Yoruba. To calculate the West Eurasian ancestry proportion, let Q be the length of the red branch, then $f4(A,C;X,D)/f4(A,C;B,D) = Q \cdot \alpha / Q = \alpha$.

**S6.5 Table. F4-ratio estimates.**

| | F4 | Z |
|---|---|---|
| **JuhoansiSan** | 0.0400 | 5.144* |
| **KhomaniSan** | 0.0391 | 4.335* |
| **TuuSan** | 0.0384 | 3.969* |

| | | |
|---|---|---|
| **MbutiPygmy** | -0.0057 | -0.658 |
| **BakaPygmy** | -0.0586 | -5.358 |
| **SouthAfricanBantu** | -0.0342 | -0.992 |
| **WestAfricanBantu** | -0.0120 | -0.359 |
| **Mandenka** | 0.0036 | 0.133 |
| **Laal** | -0.0197 | -0.549 |
| **Dinka** | -0.0176 | -0.59 |
| **EastAfricanBantu** | 0.1492 | 3.977* |
| **Toubou** | 0.3136 | 7.332* |
| **Saharawi** | 0.8495 | 14.321* |
| **Libyan** | 0.7597 | 13.451* |

Positive proportions with significant values (Z>3) are marked with an asterisk.

## S7) PSMC

We applied the Pairwise Sequentially Markovian Coalescent (PSMC) model to our genomes in order to infer estimates of the effective population size through time for each population, on the basis of the genomic regions found with similar local density of heterozygous sites in each sample [28]. In order to facilitate visualization, we selected the following individuals as representative of their populations: Yoruba_HGDP00936, Mandenka_HGDP01284 and Dinka_DNK07, Moreover, Eurasian populations are illustrated by French_HGDP00521 and Han_HGDP00778. We prepared the autosomal sequences for being used on the PSMC as follows: we obtained the consensus diploid sequence using *samtools* (*`samtools mpileup -C50`*); we excluded positions with less than one third or more than twice the average coverage independently in each sample; and we masked all non-callable positions (*`bcftools view -c - | vcfutils.pl vcf2fq -d $min -D $max`*). We run PSMC with following parameters: `psmc -N25 -t15 -r5 -p "4+25*2+4+6"`. We used two different mutation rates per generation, of $2.5x10^{-8}$ and $1.2x10^{-8}$, with a generation time of 25 years to scale time and $N_e$ parameters (**Figure 5**, **S7.1** and **S7.2 Figs.**). We also used a different set of time parameters to resolve particular periods at a finer time scale *`(-p "4+50*1+4+6")`* (**S7.3 Fig.**). Because we have detected signals of European ancestry in several sub-Saharan individuals (see **S6 section**), we aimed to explore if our observations from the PSMC analysis are affected by this confounding factor. To do that, we run PSMC with same parameters but excluding in each genome those regions with an inferred European ancestry (**S7.4 Fig.**). These fragments were identified through local ancestry analysis, using SHAPEIT2 [29] to phase the data and RFMix v1.5.4 [30] to detect the tracts using AFR (GWD, YRI, ESN, LWK) and EUR (CEU, GBR, IBS, TSI, FIN) superpopulations from the 1,000 Genome Project phase III (http://www.internationalgenome.org/data-portal/population) as references.

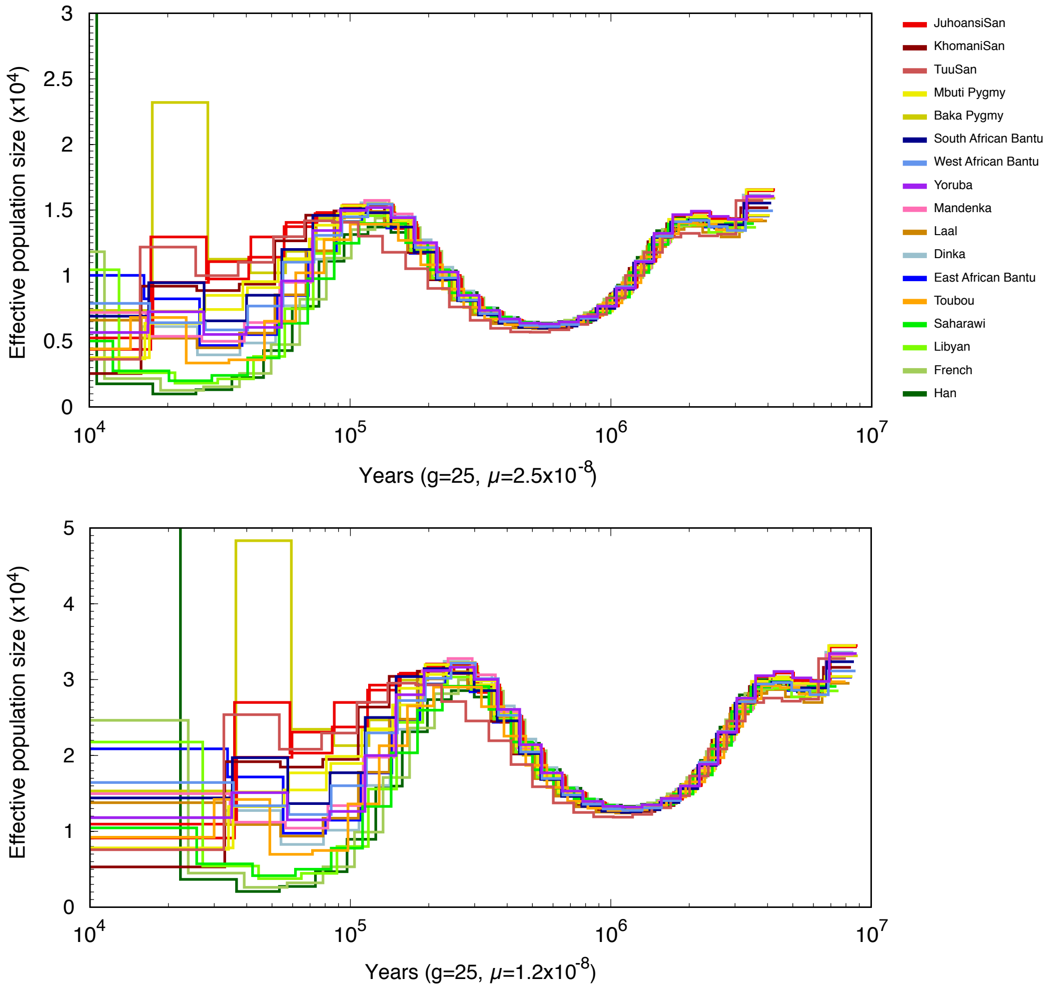


**S7.1 Fig. PSMC analysis on 15 African and 2 Eurasian populations.** Ne and time have been scaled with a generation time of 25 years and a mutation rate of 2.5x10-8 (above) or 1.2x10-8 (below).

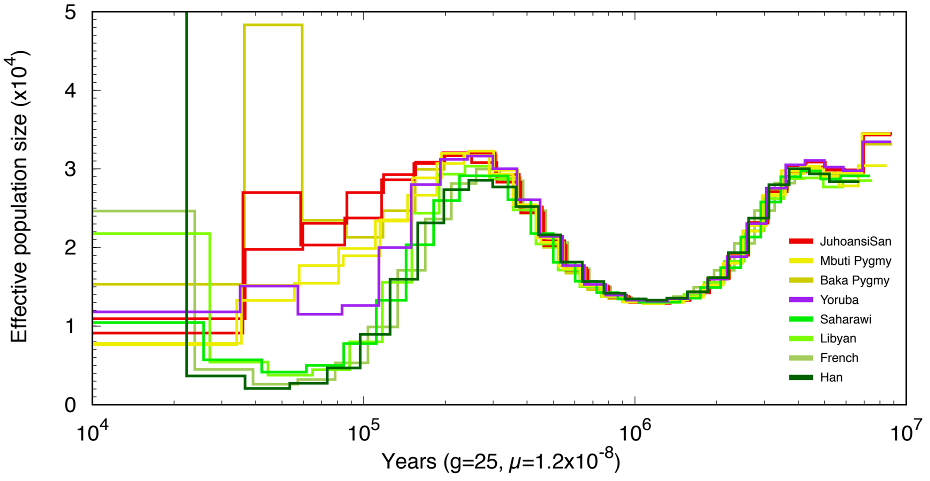


**S7.2 Fig. PSMC analysis on eight populations.** Ne and time have been scaled with a generation time of 25 years and a mutation rate of 1.2x10-8.

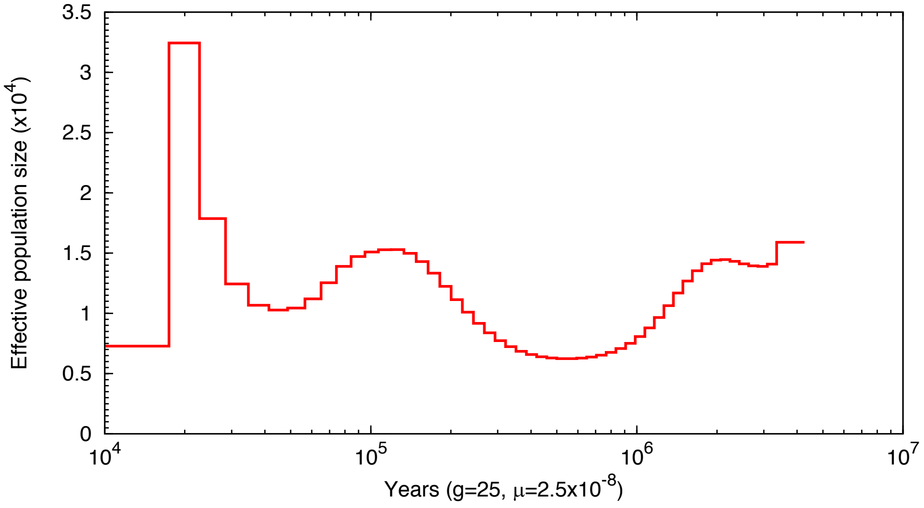


**S7.3 Fig. PSMC at fine-scale resolution for Baka pygmy.** A gradual increase of Ne from 40.000 to 20.000 years ago is shown.

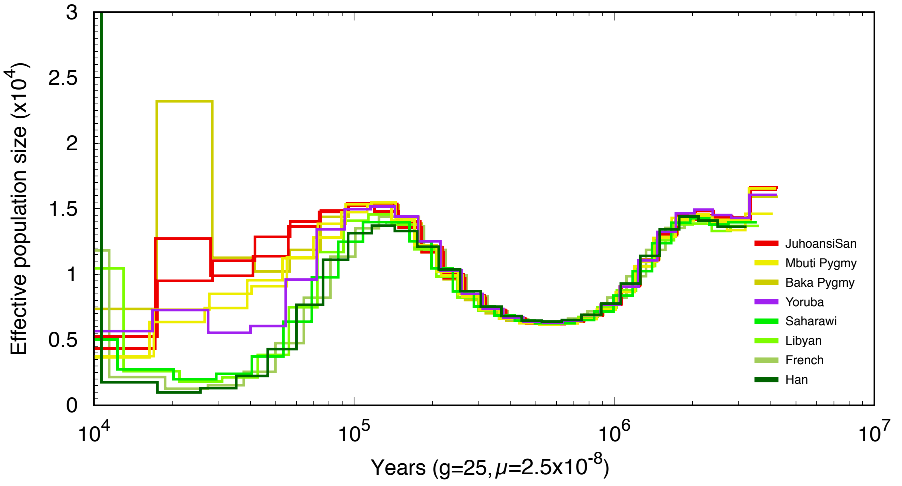


**S7.4 Fig. PSMC analysis with masked European ancestry in sub-Saharan individuals.**

## S8) Neanderthal and Denisova introgression

We tested introgression of archaic hominins, particularly of Neanderthals and Denisovans, into African modern populations using the D-statistics test (see **S6 section** for further general description). We used the chimpanzee genome as the outgroup, a high-coverage Neanderthal or a Denisovan sequence as the donor and our 25 samples as the recipients. A Papuan individual was also included as a control recipient sample for the Denisovan ancestry.

The Papuan, Neanderthal and Denisova samples were obtained from the Max Plank Institute for Evolutionary Biology repositories, particularly from http://cdna.eva.mpg.de/denisova/VCF/human/, http://cdna.eva.mpg.de/neandertal/altai/AltaiNeandertal/VCF/, and http://cdna.eva.mpg.de/denisova/VCF/hg19_1000g/, respectively. Biallelic SNPs included in the downloaded VCFs were merged with our dataset after filtering out sites that were not present in our callable genome (see **S2 section**). Chimpanzee alleles for our variant positions were retrieved from the alignments of the chimpanzee assembly against hg19, downloaded from http://hgdownload.cse.ucsc.edu/goldenPath/hg19/vsPanTro4/reciprocalBest.

Results for the D-statistics are shown in **S8.1 Fig**. As previously reported [31], Neanderthal introgression is detected in all non-sub-Saharan samples, with Oceanians and East Asians carrying a stronger signal than Europeans and North Africans. The Denisovan signal is significantly higher in Papuans than in the rest of populations as previously described. Additionally, the Denisovan signal is also higher in East Asians, Europeans, and North Africans compared to sub-Saharans, potentially due to Denisovans carrying Neanderthal introgression themselves [32].

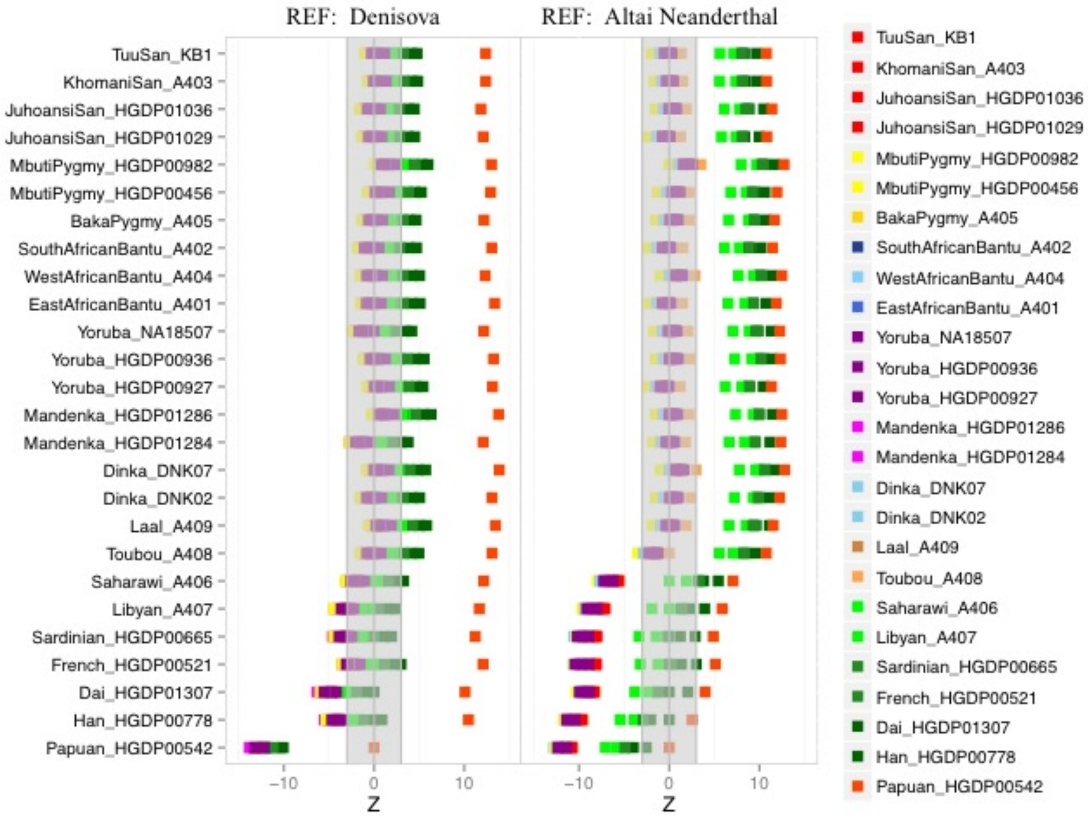


**S8.1 Fig. Denisova and Neanderthal D-statistics with the phylogeny (((P1,P2), Denisova or Neanderthal), Chimp),** where P1 is represented by colors and P2 in the Y axis. Non-significant Z-score values are shown within a grey box.

$F_4$-ratio analysis was applied to calculate the proportion of Neanderthal introgression. This statistic requires an unadmixed population in order to ascertain sites that are indeed introgressed. Previous studies [33, 34] have chosen the Yoruba as an unadmixed population, despite admitting Yoruba might contain a small amount of Neanderthal introgression. We computed the $F_4$-ratio statistics as f4(Denisova, Chimp; X = African population, Yoruba) / f4(Denisova, Chimp; Neanderthal, Yoruba) for estimating the proportion of Neanderthal ancestry in the X sample. All African samples were found to have a non-significant $F_4$ value (|Z| < 3) with the only exception of the Libyan (1.59%, Z=3.906) (**S8.1 Table**). Therefore, this analysis corroborates the D-statistics results, where no sub-Saharan samples were found to carry any Neanderthal ancestry signal.

**S8.1 Table. F4 estimates for Neanderthal introgression**

| | F4 | Z |
|---|---|---|

| | | |
|---|---|---|
| **Ju/'hoansiSan_HGDP01029** | -0.000557 | -0.122 |
| **Ju/'hoansiSan_HGDP01036** | 0.000343 | 0.073 |
| **KhomaniSan_A403** | -0.003341 | -0.674 |
| **TuuSan_KB1** | -0.00219 | -0.457 |
| **MbutiPygmy_HGDP00456** | -0.004327 | -0.982 |
| **MbutiPygmy_HGDP00982** | -0.007988 | -1.822 |
| **BakaPygmy_A405** | -0.000652 | -0.154 |
| **SouthAfricanBantu_A402** | 0.001836 | 0.456 |
| **WestAfricanBantu_A404** | 0.000378 | 0.101 |
| **Mandenka_HGDP01284** | 0.006891 | 1.708 |
| **Mandenka_HGDP01286** | -0.005911 | -1.514 |
| **Laal_A409** | -0.004211 | -1.106 |
| **Dinka_DNK02** | 0.000806 | 0.207 |
| **Dinka_DNK07** | -0.003183 | -0.779 |
| **EastAfricanBantu_A401** | 0.000846 | 0.212 |
| **Toubou_A408** | 0.001077 | 0.265 |
| **Saharawi_A406** | 0.010496 | 2.473 |
| **Libyan_A407** | 0.01594 | 3.906* |
| **French_HGDP00521** | 0.013318 | 3.144* |
| **Sardinian_HGDP00665** | 0.01841 | 4.163* |
| **Han_HGDP00778** | 0.023755 | 5.051* |
| **Dai_HGDP01307** | 0.027259 | 5.996* |

Positive proportions with significant values (Z>3) are marked with an asterisk.

## S9) Demographic model

Approximate Bayesian Computation (ABC) is a statistical framework that is used for i) inferring posterior distributions of parameters of a given model, and/or ii) comparing models when data is generable from the models of interest by means of a simulator if a closest form of the likelihood of the data given the model does not exist [35]. ABC has been shown to be extremely helpful for distinguishing among competing models and for parameter estimation in the field of population genetics, where the likelihood of the observed genetic variation given an evolutionary model is not usually computable, but it is computationally affordable to produce simulated DNA sequences from the model [36].

The basic ABC rejection algorithm proposes comparing real data with simulated data by means of a set of summary statistics (SS), and ascertaining the parameters of the simulations whose $||SS_{obs} - SS_{sim}|| = 0$ to generate the posterior distribution of the parameters/models. In practice, this requirement implies that the basic ABC rejection algorithm is prohibitively computationally expensive and several modifications have been proposed in order to reduce the number of required simulations. Among them, we highlight generalizing the classical ABC rejection algorithm by introducing an error threshold ($||SS_{obs} - SS_{sim}|| < \varepsilon$) and weight the accepted simulations depending on $\varepsilon$ [37].

There are three requirements to conduct ABC: i) prior distributions of each parameter considered in the model, ii) a simulator suitable for producing data similar to the observed one, and iii) a set of SS obtained from the data that is informative of the parameters of the model. Identifying the proper set of SS for the specified model and parameters of interest is an active subject of analysis in the ABC community [38] since an inefficient specification of the SS can negatively affect the output of ABC analysis. Several approaches have been proposed for minimizing the number of required SS as well as for identifying the most informative SS for a given parameter or model [39]. In the present study we used the approach developed by Mondal et al [40] based on the algorithm proposed by Jiang et al [41] to recover informative SS by means of applying a Deep Learning (DL) approach. The basic idea is that DL can identify extremely complex patterns in a raw representation of the data that fits the parameter/model that was used to generate the data thanks to its non-linear nature. By definition, this prediction must be the most informative SS of the parameter/model we want to estimate. Thus, the predicted DL value can be used in the classical ABC framework as SS (SS-DL).

A simplified representation of the ABC-DL algorithm is shown in **S9.1 Fig**.

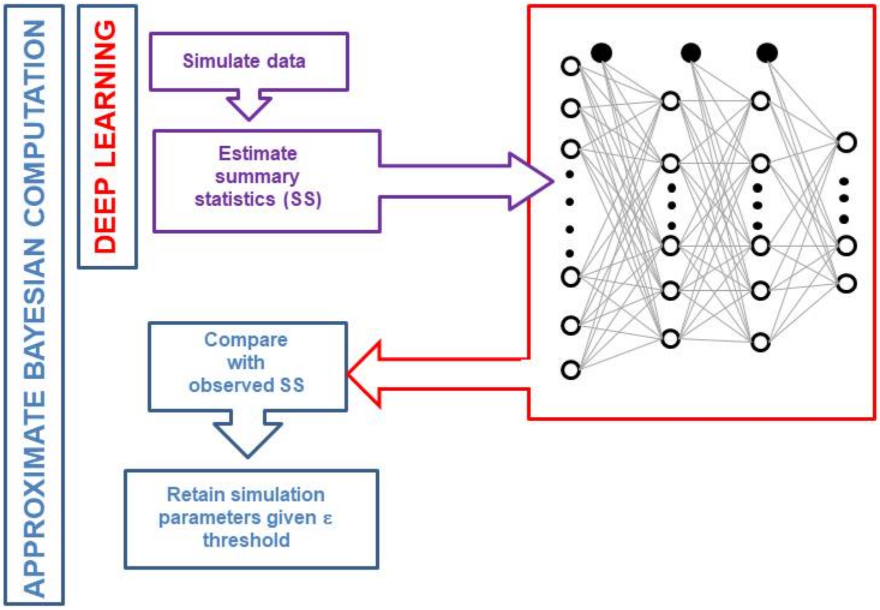


**S9.1 Fig. Approximate Bayesian Computation coupled to Deep Learning.** As a first step, we generated simulations from the prior distributions of parameters and estimated raw summary statistics. These simulations are then used for training a DL, whose output is the expected value of the parameter/model that generated the simulated data. Once the DL is trained, we generated new simulations from the prior distributions, predict the output in the trained DL and compare it with the predicted output using the observed data. The parameters/model that generated the simulation are retained given the ε threshold.

A raw summary statistic that recapitulates many aspects of old and recent demographic events is the joint multidimensional site frequency spectrum distribution among populations (*jSFS*) [42]. *jSFS* quantifies the number of SNPs that follow a particular distribution of derived alleles among the considered populations. *jSFS* has been previously used for estimating demographic parameters of interest in composite likelihood scheme approaches [42, 43]. However, given the multidimensional nature of the *jSFS* distribution, the number of observed patterns exponentially increases with the number of individuals and considered populations. Because the presence of high dimensionality in the SS can be detrimental for ABC analyses, here we considered a supervised deep learning (DL) approach for reducing the dimensionality of the raw SS, i.e. we use the output *f(jSFS)* of a trained DL as summary statistic.

A potential caveat of the DL approach for extracting informative SS is the fact that the DL is trained with simulated data from proposed (known) models and the obtained value is compared with the output from observed data that is likely produced by a much more complex model than

the considered ones. In this context, one of the proposed models is at best a nested model of the real one that generated the observed data. However, this difference between the data used for training and the final observed data that we want to classify can produce unpredictable results of the DL when applied to the real data. Mondal et al [40] overcome this problem by means of implementing a version of the noise injection algorithm. Applying the same approach, we split the observed dataset in two, if possible, one of them comprising samples that will be used to add "observed data-like" noise to the simulated *jSFS*, and a second one that will be used for the estimation of the posterior distributions using the ABC approach. This is the case for all populations except Neanderthals, Europeans, and East Asians, for which we have only one sample. At each iteration of the DL training algorithm, we define the training data for the *j* cell of the *jSFS* of simulation *i* from a given model *s* as follows:

$$SFS_{i,j}^{s} = (1-\alpha_i)SFS_{i,j}^{s} + \alpha_i SFS_{i,j}^{r}$$

where $\alpha$ is sampled from a uniform distribution in the range [0,0.2] and r corresponds to the observed data.

**1. Demographic model comparison**

We considered six demographic topologies that could explain the actual genetic diversity observed in African and Eurasian populations (**Figure 4**). The currently assumed topology (model A), which includes Neanderthal introgression into Eurasian populations, was tested versus five other models (models B-F) that incorporate additional introgressions into African populations from a maximum of two unknown archaic hominins.

The *jSFS* was computed considering 11,642 genomic regions that correspond to 393.5 Mbp of the genome with a mean fragment length of 33.8 kbp (**S9.2 Fig.**). These fragments are ascertained after i) excluding CpG islands (using http://epigraph.mpi-inf.mpg.de/download/CpG_islands_revisited/ as described in [44]) and genes (coordinates for GRCh38.p7 downloaded from Ensembl) from the callable genome, ii) concatenating the outcome fragments that are less than 5 kbp apart, and iii) imposing a minimum distance of 20 kbp between these concatenated genomic regions in order to be considered for the analysis. It must be taken into account that, even if the introgressed fragments could be larger or smaller than these 20 kbp, it is not relevant for our analyses, since we ultimately collapse the jSFS information of each fragment into a whole-genome version, so we are only considering the average amount of introgression over fragments in the genome.

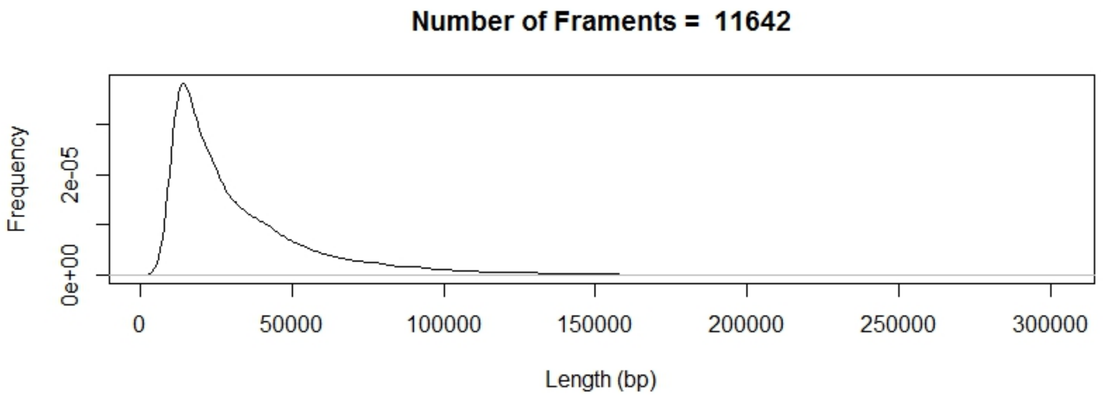


**S9.2 Fig. Length distribution of the genomic fragments used in the analysis.**

Each simulation comprised 11,642 genomic regions that follows same length distribution than the one observed in our real data (**S9.2 Fig.**). We used fastsimcoal2 software, which is fast for simulating large genomic regions under neutrality [43, 45]. Mutation rate per base and generation at each genomic region was ascertained from a normal distribution with mean 1.61e-8 and standard deviation = 0.13e-8 [46]. Since each fragment had a different proportion of callable positions, we scaled the mutation rate at each fragment by the length of callable positions. Recombination rate was set constant at 1cM/Mb.

The prior distributions used for each model are shown in **S9.1 Table**.

**S9.1 Table. Parameters and prior distributions of the six considered models in Figure** 4

| Parameters | Distribution | Model | | | | | |
|---|---|---|---|---|---|---|---|
| | | A | B | C | D | E | F |
| MigrationFrench_WestAfrica | U(0.0,5.0E-5) | X | X | X | X | X | X |
| MigrationFrench_Mbuti | U(0.0,5.0E-5) | X | X | X | X | X | X |
| MigrationFrench_Kho | U(0.0,5.0E-5) | X | X | X | X | X | X |
| MigrationWestAfrica_Mbuti | U(0.0,5.0E-5) | X | X | X | X | X | X |
| MigrationWestAfrica_Kho | U(0.0,5.0E-5) | X | X | X | X | X | X |
| MigrationMbuti_WestAfrica | U(0.0,5.0E-5) | X | X | X | X | X | X |

| | | | | | | | |
|---|---|---|---|---|---|---|---|
| MigrationMbuti_Kho | U(0.0,5.0E-5) | X | X | X | X | X | X |
| MigrationKho_Mbuti | U(0.0,5.0E-5) | X | X | X | X | X | X |
| NeKho | U(10000.0,80000.0) | X | X | X | X | X | X |
| NeMbt | U(10000.0,80000.0) | X | X | X | X | X | X |
| NeAfrica | U(10000.0,80000.0) | X | X | X | X | X | X |
| NeFrench | U(2000.0,40000.0) | X | X | X | X | X | X |
| NeHan | U(2000.0,40000.0) | X | X | X | X | X | X |
| NeAltai | U(1000.0,40000.0) | X | X | X | X | X | X |
| NeNI | U(1000.0,40000.0) | X | X | X | X | X | X |
| NeDenisova | U(1000.0,40000.0) | X | X | X | X | X | X |
| NeDI | U(1000.0,40000.0) | X | X | X | X | X | X |
| NeXe | U(1000.0,40000.0) | X | X | X | X | X | X |
| tSplitFrench_Han | U(1000.0,3000.0) | X | X | X | X | X | X |
| NeFrench_Han | U(1000.0,5000.0) | X | X | X | X | X | X |
| tSplitEurasia_WestAfrica | U(tSplitFrench_Han,6000.0) | X | X | X | X | X | X |
| NeEurasia_WestAfrica | U(1000.0,5000.0) | X | X | X | X | X | X |
| tSplitEurasia_WestAfrica_Mbuti | U(tSplitEurasia_WestAfrica,7000.0) | X | X | X | X | X | X |
| NeEurasia_WestAfrica_Mbuti | U(1000.0,20000.0) | X | X | X | X | X | X |
| tOriginHumans | U(tSplitEurasia_WestAfrica_Mbuti,9000.0) | X | X | X | X | X | X |
| NeEurasia_WestAfrica_Mbuti_KhoKho | U(1000.0,20000.0) | X | X | X | X | X | X |
| tSplitNeander_NI | U(3000.0,7000.0) | X | X | X | X | X | X |

| | | | | | | | |
|---|---|---|---|---|---|---|---|
| NeAltai_NI | U(1000.0,40000.0) | X | X | X | X | X | X |
| tSplitDenisova_DI | U(3000.0,7000.0) | X | X | X | X | X | X |
| NeDenisova_DI | U(1000.0,40000.0) | X | X | X | X | X | X |
| tSplitNeander_Denosova | U(10000.0,20000.0) | X | X | X | X | X | X |
| NeNeanderthal_Denisova | U(7000.0,40000.0) | X | X | X | X | X | X |
| tSplitArchaics_humans | U(tSplitNeander_Denosova,30000.0) | X | X | X | X | X | X |
| NeBeginingOfTimes | U(7000.0,40000.0) | X | X | X | X | X | X |
| tIntrogressionEarlyHumans_Neanderthals | U(tOriginHumans,10000.0) | X | X | X | X | X | X |
| IntrogressionEarlyHumans_Neanderthal | U(1.0E-4,0.05) | X | X | X | X | X | X |
| tIntrogressionNeanderthal_Eurasia | U(tSplitFrench_Han,tSplitEurasia_WestAfrica) | X | X | X | X | X | X |
| IntrogressionNeanderthal_Eurasia | U(1.0E-4,0.05) | X | X | X | X | X | X |
| tIntrogressionNeanderthal_Han | U(800.0,tSplitFrench_Han) | X | X | X | X | X | X |
| IntrogressionNeanderthal_Han | U(1.0E-4,0.05) | X | X | X | X | X | X |
| tIntrogressionErectus_Denisovan | U(1423.0,2413.0) | X | X | X | X | X | X |
| IntrogressionErectus_Denisovan | U(0.0,0.05) | X | X | X | X | X | X |
| NeErectus_Hominin | U(5000.0,40000.0) | X | X | X | X | X | X |
| tHominin_Erectus | U(34482.0,68965.0) | X | X | X | X | X | X |
| NeXaGhostAfrican | U(1000.0,40000.0) | | X | | | X | X |
| tSplitXaGhostAfricanHuman | U(tSplitEurasia_WestAfrica_Mbuti,tSplitArchaics_humans) | | X | | | X | X |
| tIntrogressionXaGhostAfrican_Kho | U(700.0,tOriginHumans) | | X | | | X | X |
| IntrogressionXaGhostAfrican_Kho | U(1.0E-4,0.1) | | X | | | X | X |

| | | | | | | |
|---|---|---|---|---|---|---|
| tIntrogressionXaGhostAfrican_Mbuti | U(700.0,tSplitEurasia_WestAfrica_Mbuti) | X | | | X | X |
| IntrogressionXaGhostAfrican_Mbuti | U(1.0E-4,0.1) | X | | | X | X |
| tIntrogressionXaGhostAfrican_West African | U(700.0,tSplitEurasia_WestAfrica) | X | | | X | X |
| IntrogressionXaGhostAfrican_WestA frican | U(1.0E-4,0.1) | X | | | X | X |
| NeGhostNeanderthal | U(1000.0,40000.0) | | X | | X | X |
| tSplitGhostNeanderthal | U(tSplitNeander_NI,tSplitNeander_Denosova) | | X | | X | |
| tIntrogressionGhostNeanderthal_Kho | U(700.0,tOriginHumans) | | X | | X | |
| IntrogressionGhostNeanderthal_Kho | U(1.0E-4,0.1) | | X | | X | |
| tIntrogressionGhostNeanderthal_Mbu ti | U(700.0,tSplitEurasia_WestAfrica_Mbuti) | | X | | X | |
| IntrogressionGhostNeanderthal_Mbut i | U(1.0E-4,0.1) | | X | | X | |
| tIntrogressionGhostNeanderthal_West African | U(700.0,tSplitEurasia_WestAfrica) | | X | | X | |
| IntrogressionGhostNeanderthal_West African | U(1.0E-4,0.1) | | X | | X | |
| NeGhostArchaic | U(1000.0,40000.0) | | | X | | X |
| tSplitGhostArchaic | U(tSplitNeander_Denosova,tSplitArchaics_huma ns) | | | X | | X |
| tIntrogressionGhost_Kho | U(700.0,tOriginHumans) | | | X | | X |
| IntrogressionGhost_Kho | U(1.0E-4,0.1) | | | X | | x |
| tIntrogressionGhost_Mbuti | U(700.0,tSplitEurasia_WestAfrica_Mbuti) | | | X | | X |
| IntrogressionGhost_Mbuti | U(1.0E-4,0.1) | | | X | | X |
| tIntrogressionGhost_WestAfrican | U(700.0,tSplitEurasia_WestAfrica) | | | X | | X |
| IntrogressionGhost_WestAfrican | U(1.0E-4,0.1) | | | X | | X |

We trained ten independent feed-forward DLs of four layers in Encog V3.4 ("Encog: Library of Interchangeable Machine Learning Models for Java and C#"). Input data corresponds to each of the *jSFS* cells considering Han, French, African, Mbuti Pygmy, Khoisan and Neanderthal populations. Each hidden layer is composed by 100 neurons that use Elliott activation functions [47] and a bias neuron. Output corresponds to one of the four proposed models. Learning was performed with Resilient Propagation and Dropout rate of 50%. Noise injection was introduced using the observed *jSFS* computed from Altai Neanderthal, Han_HGDP00778, French_HGDP00521, Mandenka_HGDP01284, MbutiPygmy_HGDP00456, and JuhoansiSan_HGDP01029. Each DL was trained with 15,000 simulations from each model, comprising a total of 90,000 training simulations. Each input neuron was standardized using all the training 90,000 training simulations**.** We run each DL until the error is <0.01 or until after 10,000 learning iterations.

We then generated 100,000 additional simulations per model as replication dataset for conducting the ABC analysis, comprising a total of 900,000 simulations. For each of these simulations we computed the output from the 10 previously trained DLs and combined the predictions to produce a unique output by bagging [48] a predicted model of the four under evaluation. The combined prediction was used as SS in the ABC analysis. We considered an error threshold of 1,000 out of 900,000 simulations and applied a multinomial logistic regression on the accepted simulations; all these analyses were conducted with the R package abc [49], using the script *postpr* with option *mnlogistic* for model comparison.

We first evaluated the power of ABC-DL for distinguishing between the proposed independent models using the output of the DL as summary statistic. To do that, we run the cv4postpr command from abc package [49] using 100 simulations per model and the same ABC parameters we used when analyzing the observed data. The cv4postpr runs the ABC using simulated data as observed data and counting the number of times that the model with the highest posterior probability is, in fact the model that generated the simulated data.

**S9.2 Table. Confusion matrix computed with the six models under evaluation. 100 randomly sampled simulations per model were used as the "observed" data input (rows) for the ABC-DL algorithm. Diagonal (in bold) shows the number of samples that are correctly assigned (with the highest posterior probability) to the model.**

| Model | A | B | C | D | E | F | P(Real Model\|Inferred Model) |
|---|---|---|---|---|---|---|---|

| | | | | | | | |
|---|---|---|---|---|---|---|---|
| **A** | **95** | 5 | 0 | 0 | 0 | 0 | 0.85 |
| **B** | 17 | **81** | 0 | 0 | 0 | 2 | 0.81 |
| **C** | 0 | 0 | **93** | 6 | 0 | 1 | 0.96 |
| **D** | 0 | 7 | 3 | **71** | 12 | 7 | 0.49 |
| **E** | 0 | 3 | 0 | 28 | **43** | 26 | 0.5 |
| **F** | 0 | 4 | 1 | 39 | 31 | **25** | 0.41 |

The confusion matrix suggests that after applying the ABC-DL approach we have high accuracy for identifying the model that produced the simulation in all the models except F, which is the most general one.

Finally, we conducted the ABC-DL analysis with the real data using Altai Neanderthal sample, Han_HGDP00778, French_HGDP00521, Mandenka_HGDP01286, MbutiPygmy_HGDP00982, and JuhoansiSan_HGDP01036.

**2. Parameter estimation for the B model**

Next, we estimated the demographic parameters of the most supported model by ABC-DL, which is the model B that considers ghost introgression from a basal archaic population in the three African populations under evaluation. To do that, for each parameter we generated 10 feed-forward DLs of four layers. The input consisted on the standardized values of the *jSFS* cells. Output consisted on the value of the considered parameter, after scaling it to be in the range between 0 and 1. Hidden layers consisted on 100 neurons with Elliot activation function with a slope of 0.01, and the output consisted on an Elliot activation function with a slope of 0.1.

We used 15,000 simulations of model B to train 10 DL of each parameter. We run each DL until the error reached 0.01 or after 10,000 iterations. Then, we generated 150,000 additional simulations of model B as replication dataset of ABC analysis; for each simulation, we used the 10 trained DLs to predict the value of the parameter that was used for generating the simulation, and generated a single prediction by ascertaining the DL showing the highest Spearman correlation between the predicted value and the parameter value used in the simulation. In the case of migrations, we scaled by the effective population size.

In order to test the performance of the ABC-DL approach for parameter estimation, we sampled 1,000 simulations at random for each parameter, conducted the ABC-DL approach using the abc script of abc package with the same parameters as the ones used with the real data, and

computed the factor2 statistic [50]. This statistic accounts for the number of times that the centrality statistic of the estimated posterior distribution (in our case, the mean) falls within the range of 50% to 200% of the real value. A value close to one indicates that the mean of the posterior distribution is close to the value used in the simulations. A value close to 0.5 indicates that the value of the mean of the estimated posterior distribution is randomly distributed.

**S9.3 Table** shows the factor2 value estimated over 1,000 simulated datasets for each parameter. We observed that for the migration parameters we obtain a factor2 that is similar to the one that would be estimated if a value taken at random from the prior distribution is used as the mean of the posterior distribution. This suggests that we cannot use the mean of the posterior distribution of these parameters as indicative of the true value.

**Table S9.3. Factor2 statistic computed for each parameter either using the mean of the posterior distribution of simulated data as observed data or taking a value at random from the prior as the mean of the posterior.**

| Parameter | factor2 using the posterior mean | factor 2 using samples from the prior as posterior mean |
|---|---|---|
| MigrationFrench_WestAfrica*Ne | 0.568 | 0.462 |
| MigrationFrench_Mbuti*Ne | 0.598 | 0.497 |
| MigrationFrench_Kho*Ne | 0.598 | 0.48 |
| MigrationWestAfrica_Mbuti*Ne | 0.613 | 0.492 |
| MigrationWestAfrica_Kho*Ne | 0.65 | 0.497 |
| MigrationMbuti_WestAfrica*Ne | 0.587 | 0.506 |
| MigrationMbuti_Kho*Ne | 0.619 | 0.518 |
| MigrationKho_Mbuti*Ne | 0.587 | 0.465 |
| NeKho | 0.954 | 0.496 |
| NeMbt | 0.955 | 0.511 |
| NeAfrica | 0.949 | 0.494 |
| NeFrench | 0.946 | 0.482 |
| NeHan | 0.936 | 0.485 |
| NeAltai | 0.829 | 0.488 |
| NeNI | 0.734 | 0.471 |
| NeDenisova | 0.833 | 0.52 |
| NeDI | 0.739 | 0.454 |

| | | |
|---|---|---|
| NeXe | 0.761 | 0.496 |
| tSplitFrench_Han | 0.988 | 0.473 |
| NeFrench_Han | 0.954 | 0.534 |
| tSplitEurasia_WestAfrica | 0.987 | 0.506 |
| NeEurasia_WestAfrica | 0.921 | 0.471 |
| tSplitEurasia_WestAfrica_Mbuti | 0.995 | 0.502 |
| NeEurasia_WestAfrica_Mbuti | 0.832 | 0.51 |
| tOriginHumans | 0.999 | 0.486 |
| NeEurasia_WestAfrica_Mbuti_KoiKho | 0.915 | 0.493 |
| tSplitNeander_NI | 1 | 0.499 |
| NeAltai_NI | 0.879 | 0.522 |
| tSplitDenisova_DI | 1 | 0.521 |
| NeDenisova_DI | 0.887 | 0.537 |
| tSplitNeander_Denosova | 1 | 0.492 |
| NeNeanderthal_Denisova | 0.934 | 0.49 |
| tSplitArchaics_humans | 0.997 | 0.49 |
| NeBeginingOfTimes | 0.952 | 0.512 |
| tIntrogressionEarlyHumans_Neanderthal | 1 | 0.498 |
| IntrogressionEarlyHumans_Neanderthal | 0.806 | 0.5 |
| tIntrogressionNI_Eurasia | 0.979 | 0.512 |
| IntrogressionNI_Eurasia | 0.876 | 0.478 |
| tIntrogressionDI_Han | 0.985 | 0.525 |
| IntrogressionDI_Han | 0.858 | 0.476 |
| tIntrogressionErectus_Denisovan | 1 | 0.483 |
| IntrogressionErectus_Denisovan | 0.753 | 0.486 |
| NeErectus_Hominin | 0.886 | 0.489 |
| tHominin_Erectus | 1 | 0.497 |
| NeXaGhostAfrican | 0.777 | 0.482 |
| tSplitXaGhostAfricanHuman | 0.965 | 0.525 |
| tIntrogressionXaGhostAfrican_Kho | 0.766 | 0.484 |
| IntrogressionXaGhostAfrican_Kho | 0.728 | 0.512 |
| tIntrogressionXaGhostAfrican_Mbuti | 0.799 | 0.499 |
| IntrogressionXaGhostAfrican_Mbuti | 0.754 | 0.489 |
| tIntrogressionXaGhostAfrican_WestAfrican | 0.804 | 0.501 |
| IntrogressionXaGhostAfrican_WestAfrican | 0.775 | 0.512 |

Finally, we applied the ABC-DL analysis to predict parameters of the model B with our real data using the following samples: Mandenka_HGDP01284, MbutiPygmy_HGDP00456, and JuhoansiSan_HGDP01029. For parameter estimation, a lineal local regression on the accepted simulations [51] using the option loclinear in the script abc from the package abc. Given the unusually large estimated values of archaic introgression in Eurasia compared to previously reported values considering a model topology similar to A, we repeated our ABC-DL also with the A model. Centrality (mean) and dispersion (95% credible interval) of the posterior distribution obtained for each parameter are shown in **S9.4 Table**.

**S9.4 Table. Mean and 95 Credible interval of the posterior distribution of each parameter in model A (the basic one) and B (the most supported one) after the ABC-DL analysis. Migrations were scaled by the effective population size of the donor population following Hamilton et al [52].**

| Parameter | MODEL B | | | MODEL A | | |
|---|---|---|---|---|---|---|
| | Mean | CI0.25 | CI0.975 | Mean | CI0.25 | CI0.975 |
| IntrogressionDI_Han | 0.04 | 0.013 | 0.05 | 0.035 | 0.015 | 0.045 |
| IntrogressionEarlyHumans_Neanderthal | 0.012 | 0.001 | 0.04 | 0.015 | 0.002 | 0.04 |
| IntrogressionErectus_Denisovan | 0.008 | 0 | 0.041 | 0.008 | 0 | 0.038 |
| IntrogressionNI_Eurasia | 0.038 | 0.017 | 0.048 | 0.011 | 0.003 | 0.036 |
| IntrogressionXaGhostAfrican_Kho | 0.041 | 0.002 | 0.095 | | | |
| IntrogressionXaGhostAfrican_Mbuti | 0.043 | 0.003 | 0.095 | | | |
| IntrogressionXaGhostAfrican_WestAfrican | 0.058 | 0.007 | 0.097 | | | |
| MigrationFrench_Kho*Ne | 0.891 | 0.1 | 1.774 | 0.949 | 0.128 | 1.831 |
| MigrationFrench_Mbuti*Ne | 0.247 | 0.007 | 0.981 | 0.356 | 0.029 | 1.099 |
| MigrationFrench_WestAfrica*Ne | 0.754 | 0.047 | 1.717 | 0.831 | 0.088 | 1.763 |
| MigrationKho_Mbuti*Ne | 1.131 | 0.079 | 3.183 | 1.949 | 0.376 | 3.616 |
| MigrationMbuti_Kho*Ne | 1.874 | 0.233 | 3.58 | 0.83 | 0.058 | 2.517 |
| MigrationMbuti_WestAfrica*Ne | 1.513 | 0.125 | 3.392 | 1.751 | 0.093 | 3.548 |
| MigrationWestAfrica_Kho*Ne | 0.482 | 0.022 | 1.863 | 1.379 | 0.302 | 2.963 |
| MigrationWestAfrica_Mbuti*Ne | 1.731 | 0.264 | 3.49 | 1.912 | 0.372 | 3.539 |
| NeAfrica | 28055.832 | 14465.936 | 69306.206 | 64135.879 | 29987.822 | 79110.404 |
| NeAltai | 26835.588 | 8989.954 | 39154.065 | 14756.756 | 3754.962 | 37245.374 |
| NeAltai_NI | 10668.93 | 3497.463 | 33114.714 | 8540.379 | 2136.611 | 30819.34 |
| NeBeginingOfTimes | 12292.994 | 7307.004 | 32187.707 | 14959.885 | 8178.439 | 29905.76 |
| NeDenisova | 22196.053 | 6575.347 | 38088.141 | 9870.921 | 2640.522 | 32488.954 |
| NeDenisova_DI | 29145.061 | 5693.298 | 39595.949 | 19797.24 | 7657.878 | 37108.853 |
| NeDI | 20715.053 | 2225 | 39099 | 21093.603 | 2165 | 38945 |
| NeErectus_Hominin | 35174.057 | 11640.399 | 39877.976 | 36939.686 | 17527.619 | 40620.58 |

| | | | | | | |
|---|---|---|---|---|---|---|
| NeEurasia_WestAfrica | 3567.603 | 1421.734 | 4933.174 | 3900.816 | 1629.135 | 4979.906 |
| NeEurasia_WestAfrica_Mbuti | 13221.864 | 3424.957 | 19625.662 | 12405.826 | 3447.489 | 19336.186 |
| NeEurasia_WestAfrica_Mbuti_KoiKho | 17890.38 | 4798.995 | 20086.72 | 17874.7 | 7732.132 | 19953.143 |
| NeFrench | 10583.673 | 4781.555 | 32470.786 | 14563.402 | 8796.328 | 32008.28 |
| NeFrench_Han | 3690.38 | 1371.526 | 4879.173 | 4079.277 | 1510.998 | 4965.59 |
| NeHan | 23632.016 | 7922.478 | 37707.526 | 12894.767 | 7409.215 | 31855.98 |
| NeKho | 28444.032 | 15655.136 | 64838.258 | 28873.238 | 17795.362 | 60631.099 |
| NeMbt | 68230.641 | 27908.711 | 79790.93 | 62086.977 | 26105.508 | 79366.865 |
| NeNeanderthal_Denisova | 22425.212 | 8628.525 | 38177.73 | 28659.561 | 10200.448 | 39634.102 |
| NeNI | 20805.823 | 1621 | 39246 | 20589.958 | 1940 | 38873 |
| NeXaGhostAfrican | 20667.436 | 2274 | 39082 | | | |
| NeXe | 20291.926 | 2074 | 38982 | 20656.896 | 2189 | 39049 |
| tIntrogressionDI_Han * | 36.46 | 23.634 | 66.019 | 40.196 | 23.987 | 70.504 |
| tIntrogressionEarlyHumans_Neanderthal * | 218.967 | 145.873 | 284.571 | 200.157 | 114.247 | 284.518 |
| tIntrogressionErectus_Denisovan * | 55.39 | 41.789 | 69.165 | 55.406 | 42.398 | 69.252 |
| tIntrogressionNI_Eurasia * | 62.865 | 39.447 | 136.387 | 66.322 | 41.144 | 140.093 |
| tIntrogressionXaGhostAfrican_Kho * | 125.939 | 25.412 | 232.817 | | | |
| tIntrogressionXaGhostAfrican_Mbuti * | 74.667 | 22.679 | 163.812 | | | |
| tIntrogressionXaGhostAfrican_WestAfrican * | 46.129 | 21.146 | 122.466 | | | |
| tHominin_Erectus * | 1372.99 | 1010.538 | 1941.34 | 1433.067 | 1020.446 | 1968.248 |
| tOriginHumans * | 190.747 | 160.785 | 245.119 | 214.914 | 182.094 | 249.914 |
| tSplitArchaics_humans * | 603.252 | 495.855 | 796.863 | 528.645 | 441.298 | 695.714 |
| tSplitDenisova_DI * | 128.386 | 88.287 | 193.26 | 148.577 | 90.772 | 200.255 |
| tSplitEurasia_WestAfrica * | 101.42 | 74.423 | 160.002 | 118.919 | 100.097 | 157.125 |
| tSplitEurasia_WestAfrica_Mbuti * | 118.004 | 85.782 | 192.441 | 131.463 | 100.325 | 187.836 |
| tSplitFrench_Han * | 41.306 | 29.838 | 81.482 | 43.653 | 32.238 | 78.842 |
| tSplitNeander_Denosova * | 426.334 | 332.767 | 538.374 | 348.734 | 293.307 | 533.894 |
| tSplitNeander_NI * | 127.93 | 88.761 | 192.228 | 117.435 | 87.926 | 186.58 |
| tSplitXaGhostAfricanHuman * | 528.529 | 230.163 | 700.06 | | | |

* Mutation rate used for simulations = 1.61e-8±0.13e-8 [44]. Time (kya) is obtained by scaling the number of generations by 29 years/generation [53].

**S9.3 Fig. Posterior distributions (black) and prior distributions (red) of the parameters of model B.**

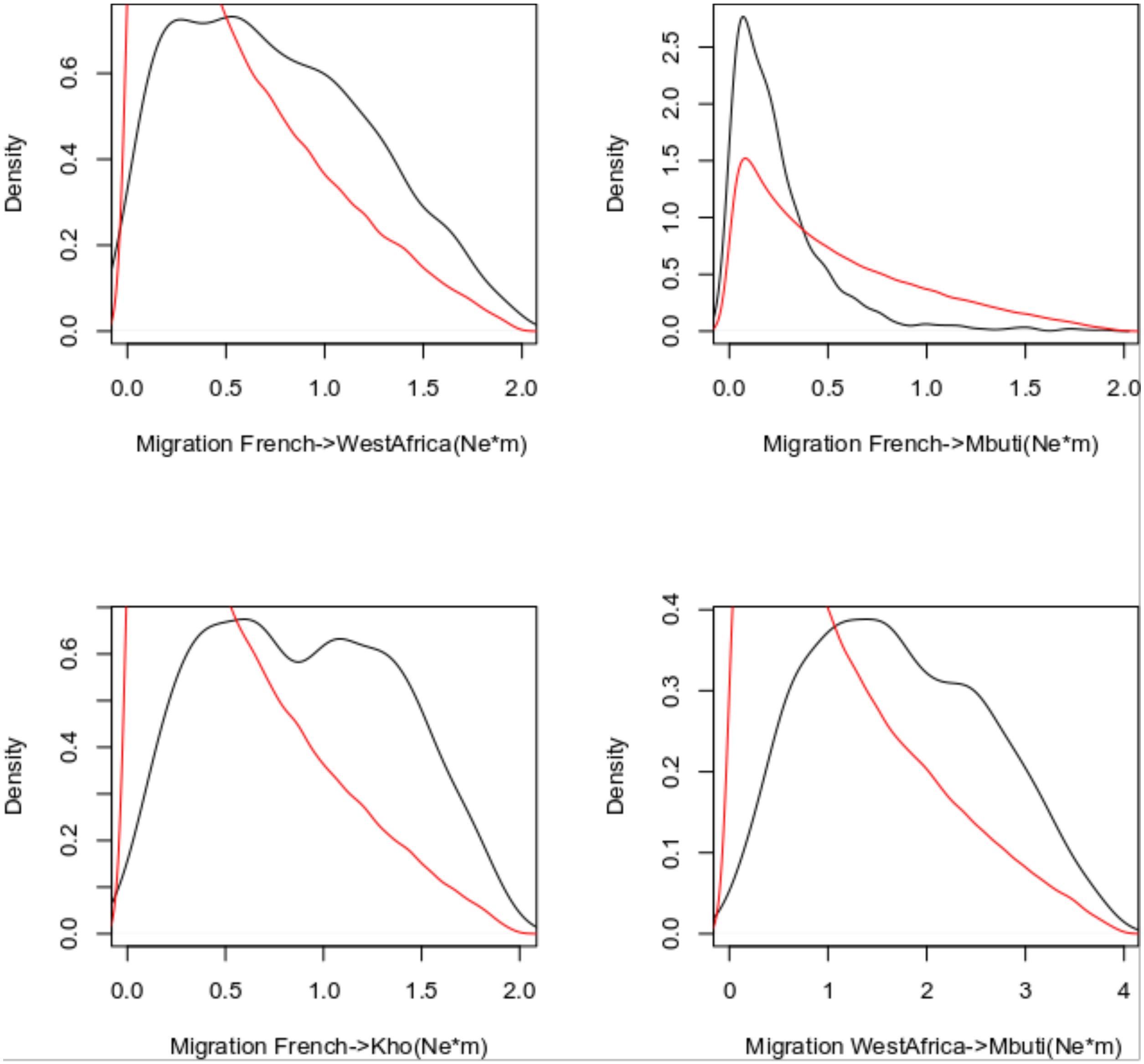
Density
0.0
0.2
0.4
0.6
0.0
0.5
1.0
1.5
2.0
Migration French->WestAfrica(Ne*m)
Density
0.0
0.5
1.0
1.5
2.0
2.5
0.0
0.5
1.0
1.5
2.0
Migration French->Mbuti(Ne*m)
Density
0.0
0.2
0.4
0.6
0.0
0.5
1.0
1.5
2.0
Migration French->Kho(Ne*m)
Density
0.0
0.1
0.2
0.3
0.4
0
1
2
3
4
Migration WestAfrica->Mbuti(Ne*m)

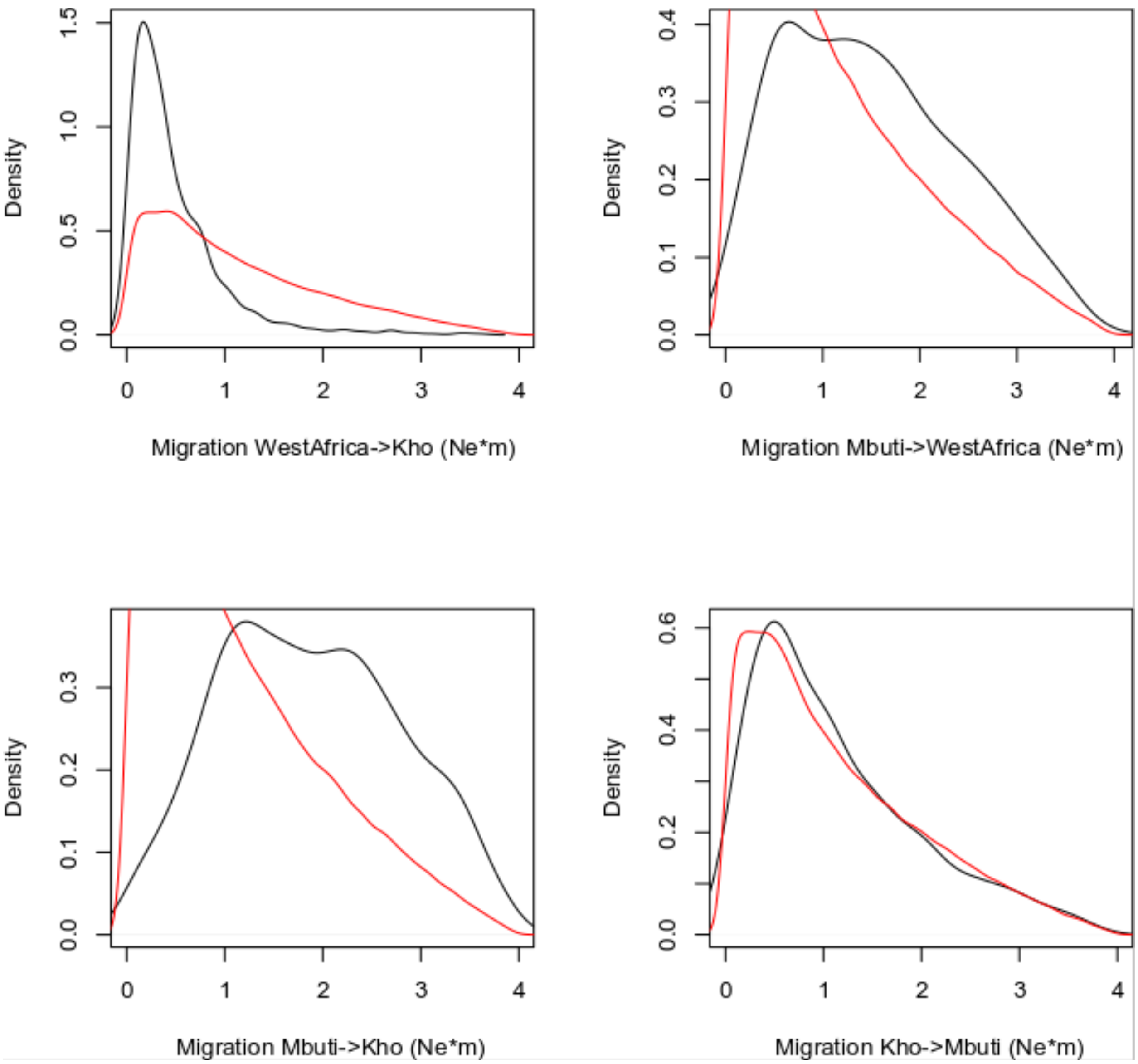
Density
Migration WestAfrica->Kho (Ne*m)
Density
Migration Mbuti->WestAfrica (Ne*m)
Density
Migration Mbuti->Kho (Ne*m)
Density
Migration Kho->Mbuti (Ne*m)

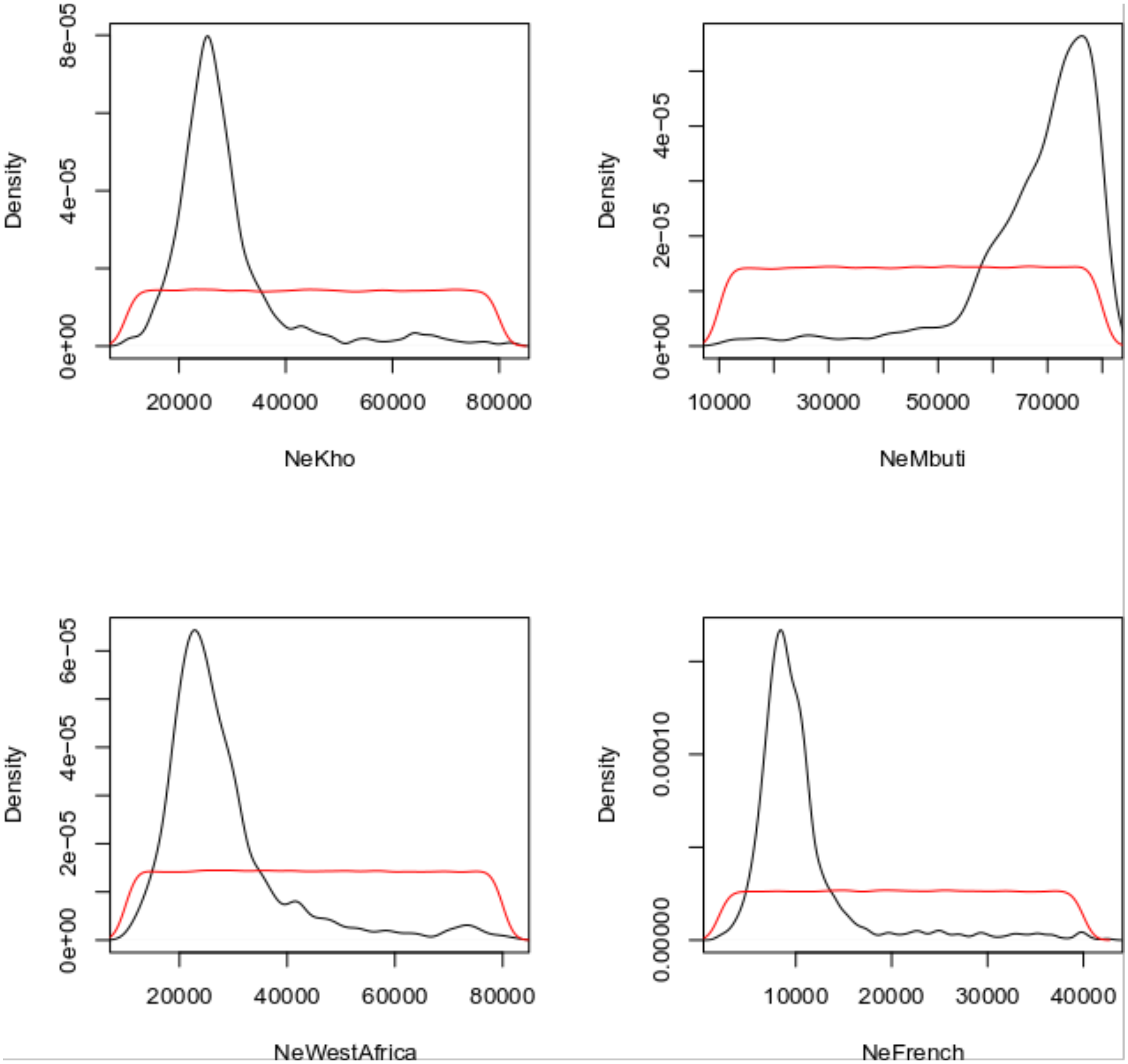
Density
8e−05
4e−05
0e+00
20000
40000
60000
80000
NeKho
Density
4e−05
2e−05
0e+00
10000
30000
50000
70000
NeMbuti
Density
6e−05
4e−05
2e−05
0e+00
20000
40000
60000
80000
NeWestAfrica
Density
0.00010
0.00000
10000
20000
30000
40000
NeFrench

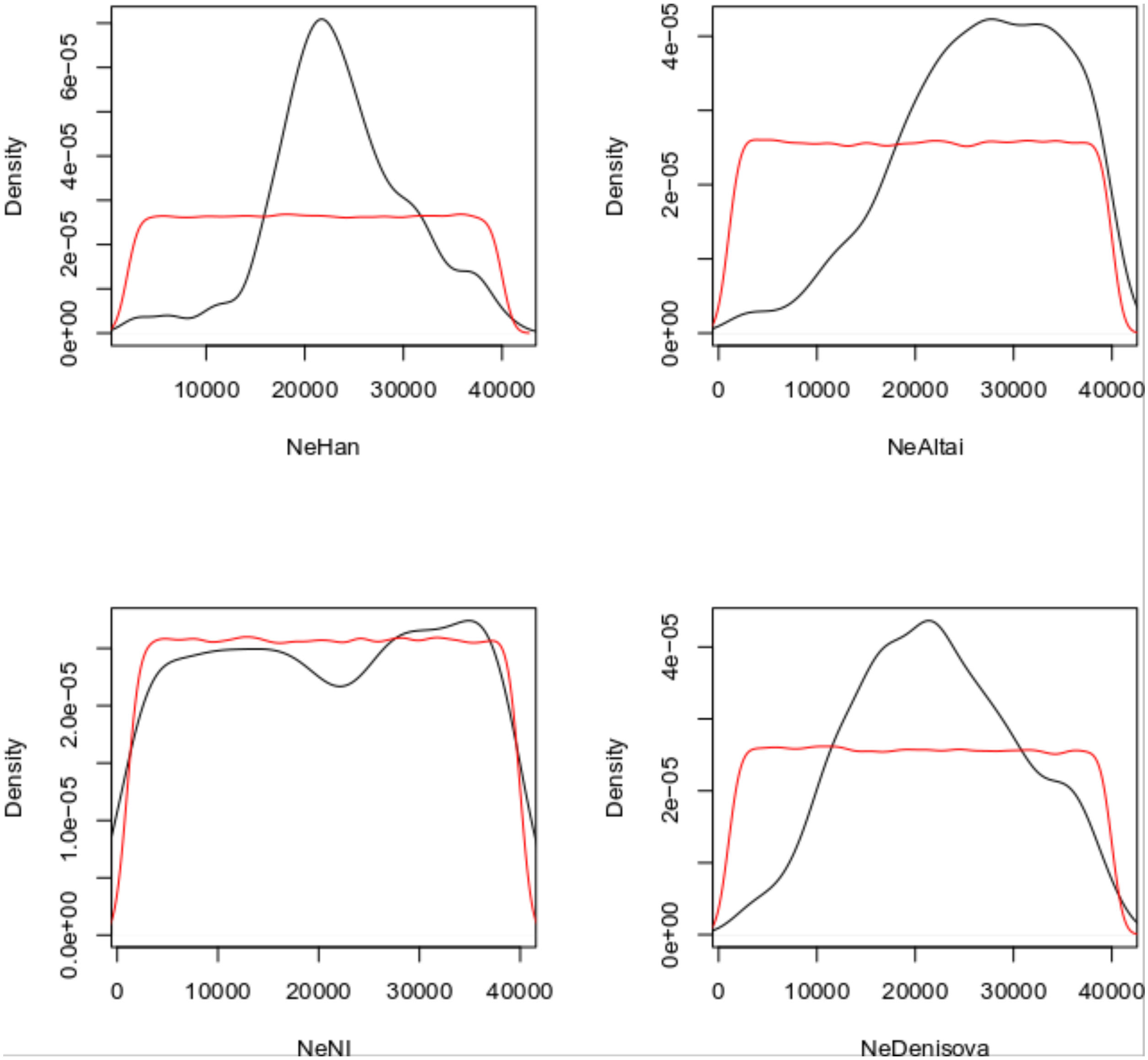
Density
0e+00
2e−05
4e−05
6e−05
10000
20000
30000
40000
NeHan
Density
0e+00
2e−05
4e−05
0
10000
20000
30000
40000
NeAltai
Density
0.0e+00
1.0e−05
2.0e−05
0
10000
20000
30000
40000
NeNl
Density
0e+00
2e−05
4e−05
0
10000
20000
30000
40000
NeDenisova

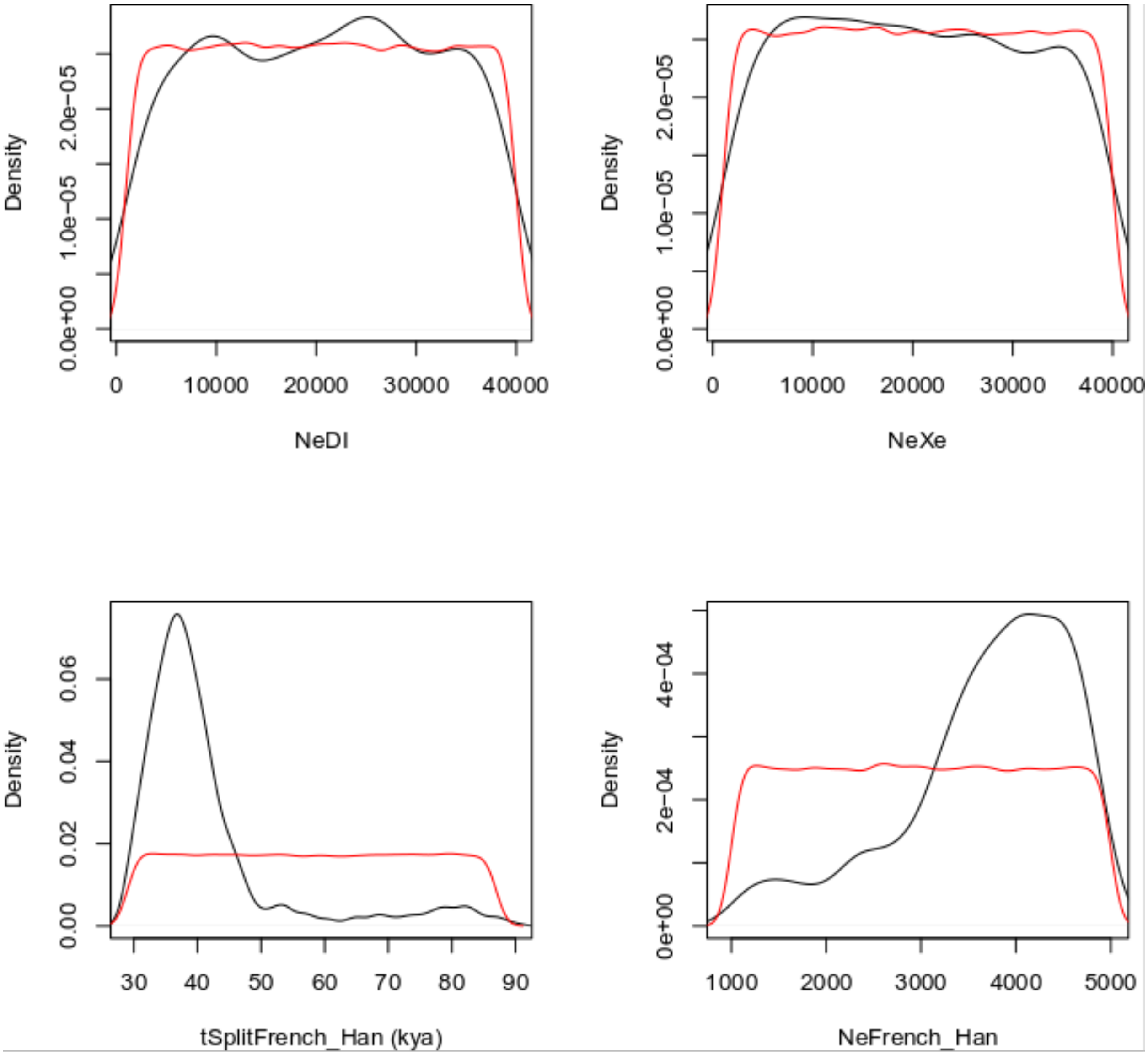
Density
0.0e+00
1.0e−05
2.0e−05
0
10000
20000
30000
40000
NeDI
Density
0.0e+00
1.0e−05
2.0e−05
0
10000
20000
30000
40000
NeXe
Density
0.00
0.02
0.04
0.06
30
40
50
60
70
80
90
tSplitFrench_Han (kya)
Density
0e+00
2e−04
4e−04
1000
2000
3000
4000
5000
NeFrench_Han

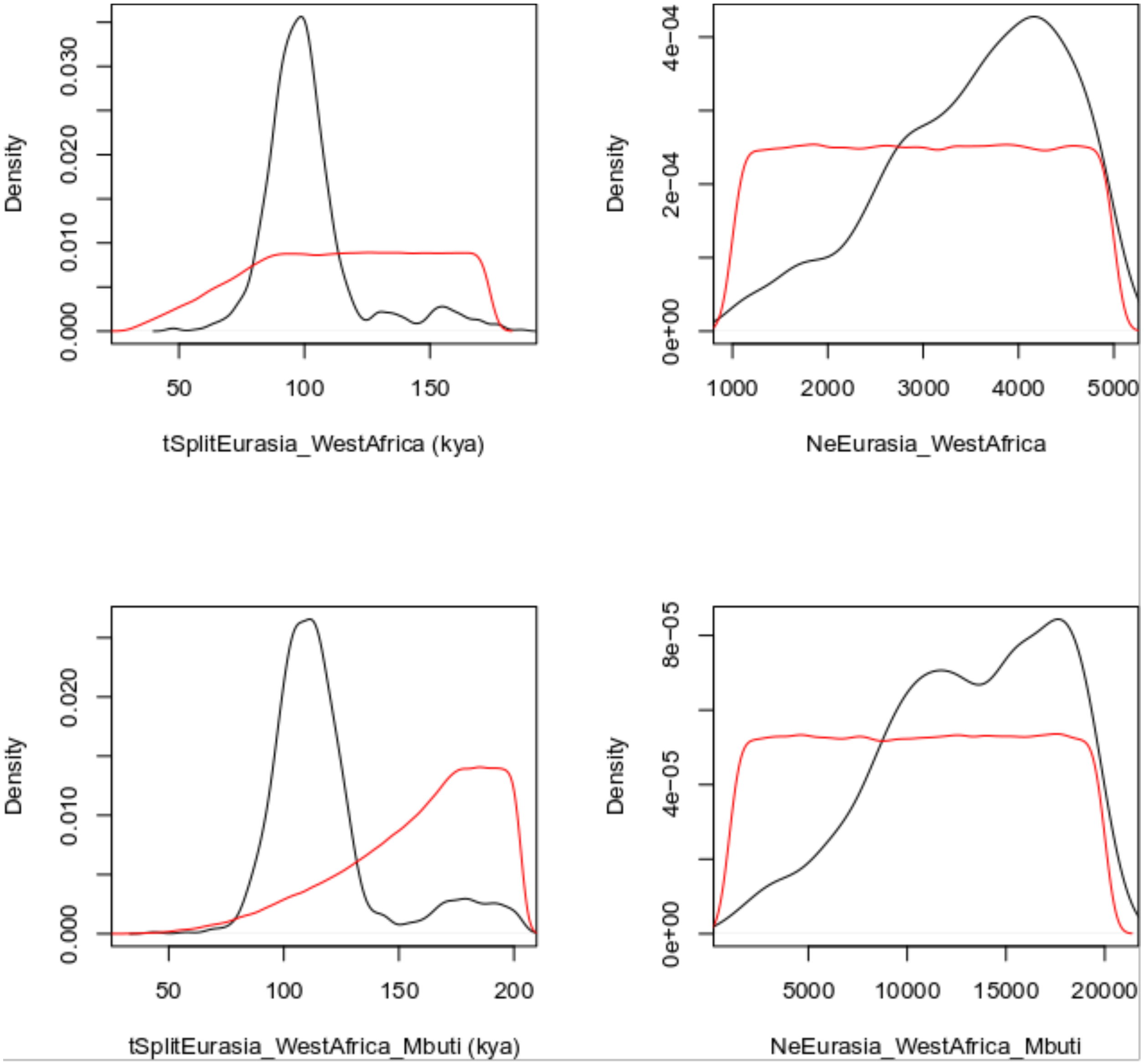
Density
tSplitEurasia_WestAfrica (kya)
Density
NeEurasia_WestAfrica
Density
tSplitEurasia_WestAfrica_Mbuti (kya)
Density
NeEurasia_WestAfrica_Mbuti

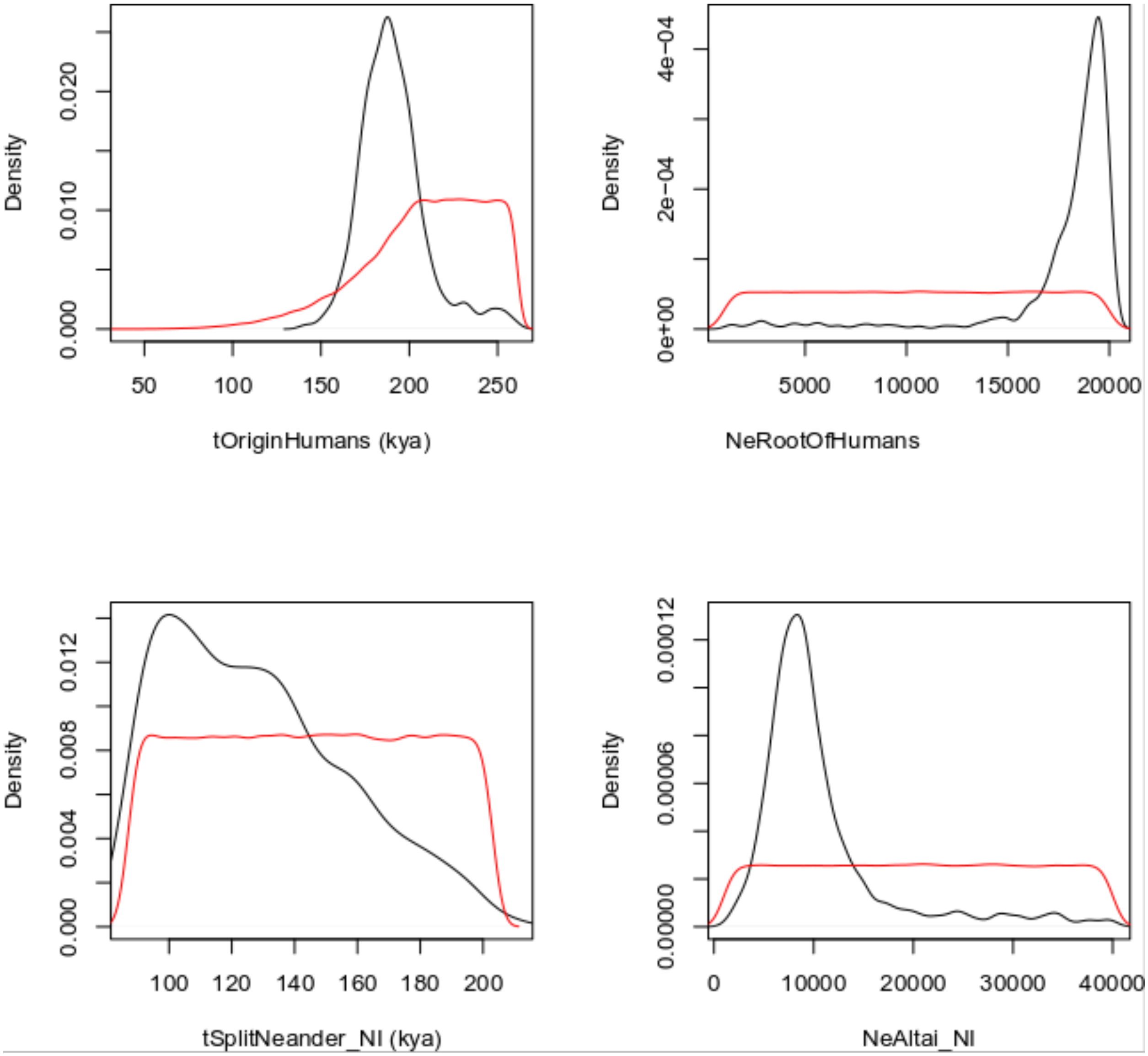
Density
0.000
0.010
0.020
50
100
150
200
250
tOriginHumans (kya)
Density
0e+00
2e−04
4e−04
5000
10000
15000
20000
NeRootOfHumans
Density
0.000
0.004
0.008
0.012
100
120
140
160
180
200
tSplitNeander_NI (kya)
Density
0.00000
0.00006
0.00012
0
10000
20000
30000
40000
NeAltai_NI

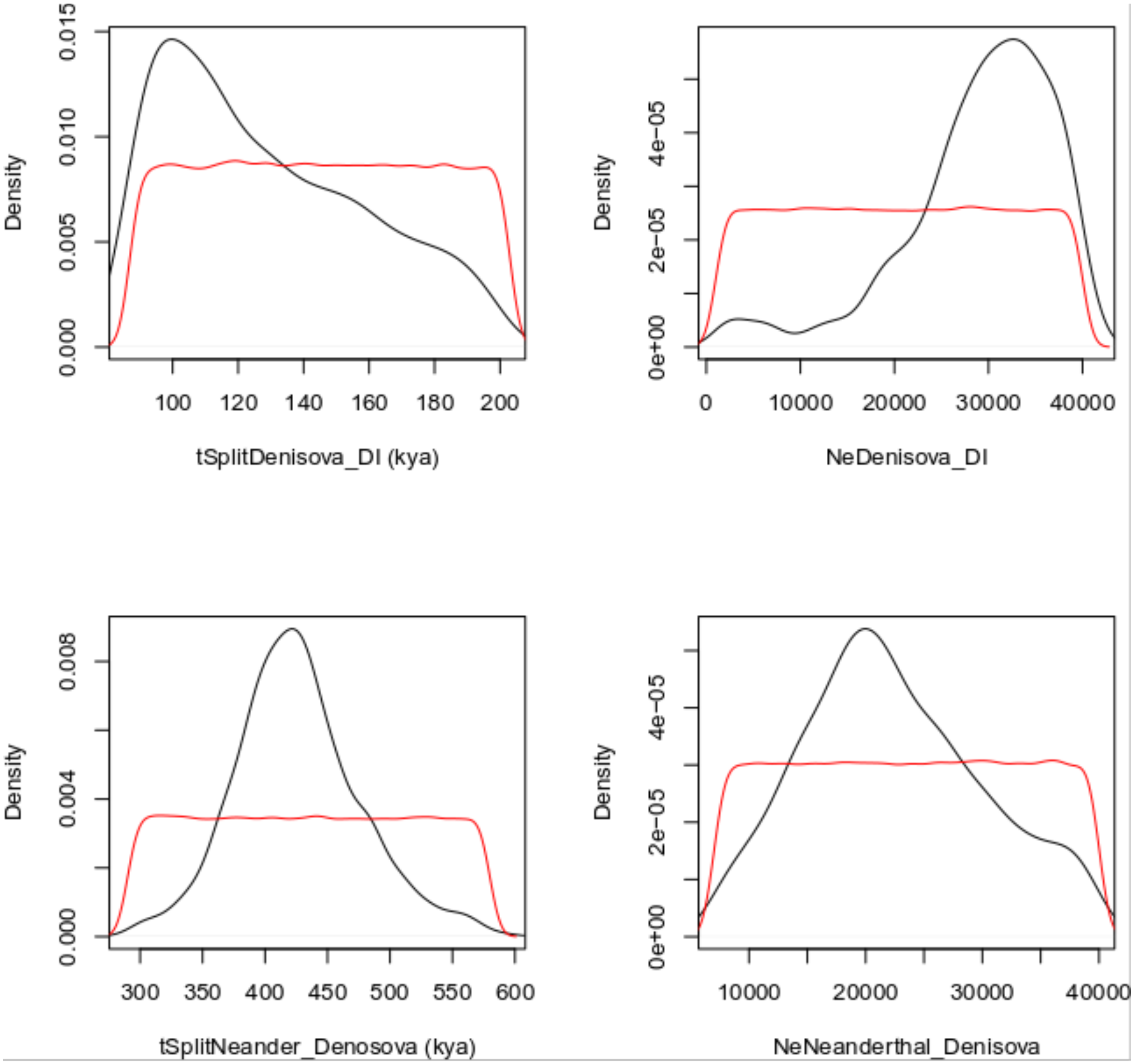
Density
0.000
0.005
0.010
0.015
100
120
140
160
180
200
tSplitDenisova_DI (kya)
Density
0e+00
2e−05
4e−05
0
10000
20000
30000
40000
NeDenisova_DI
Density
0.000
0.004
0.008
300
350
400
450
500
550
600
tSplitNeander_Denosova (kya)
Density
0e+00
2e−05
4e−05
10000
20000
30000
40000
NeNeanderthal_Denisova

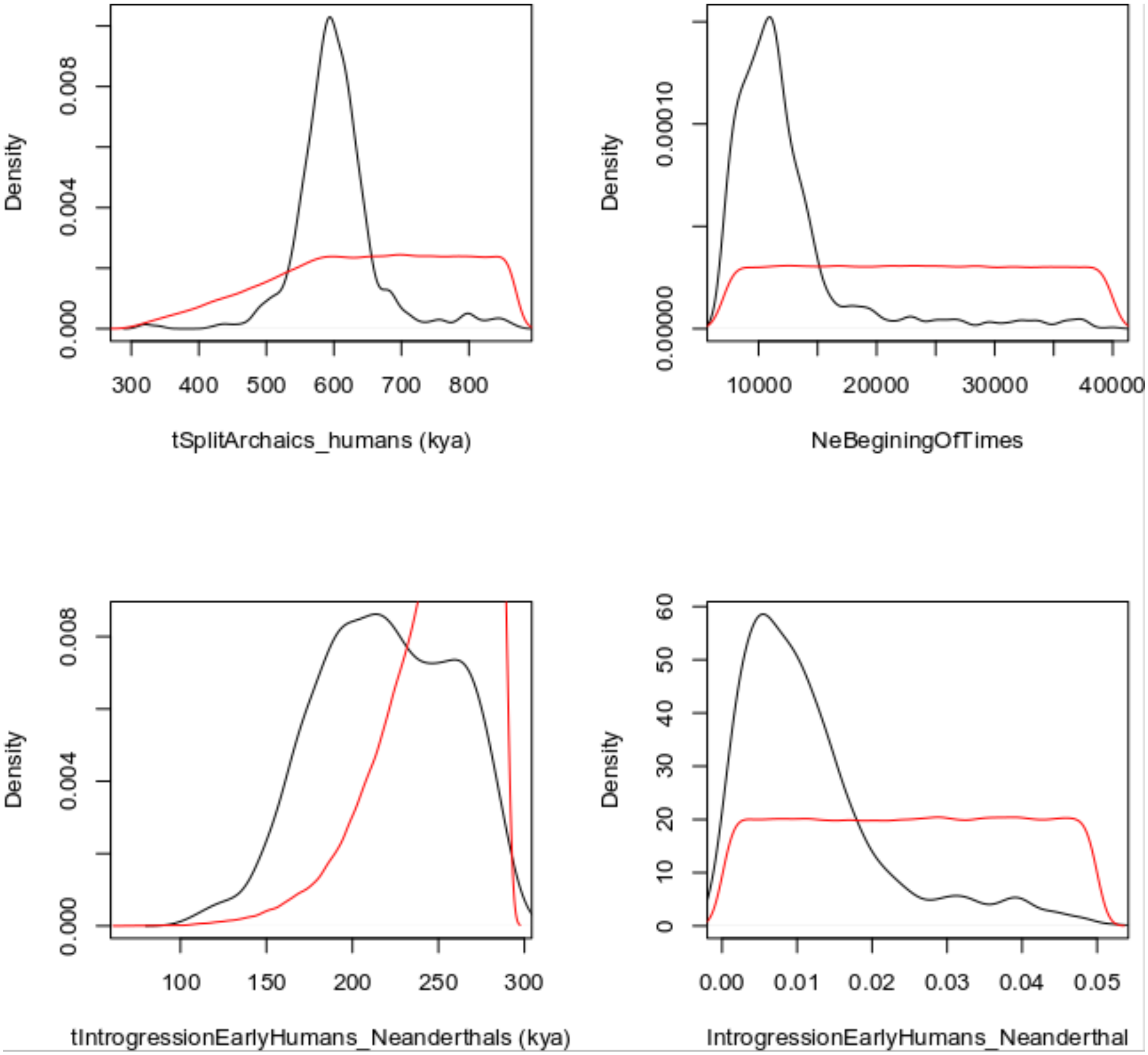

Density
0.000
0.004
0.008
300
400
500
600
700
800
tSplitArchaics_humans (kya)
Density
0.00000
0.00010
10000
20000
30000
40000
NeBeginingOfTimes
Density
0.000
0.004
0.008
100
150
200
250
300
tIntrogressionEarlyHumans_Neanderthals (kya)
Density
0
10
20
30
40
50
60
0.00
0.01
0.02
0.03
0.04
0.05
IntrogressionEarlyHumans_Neanderthal

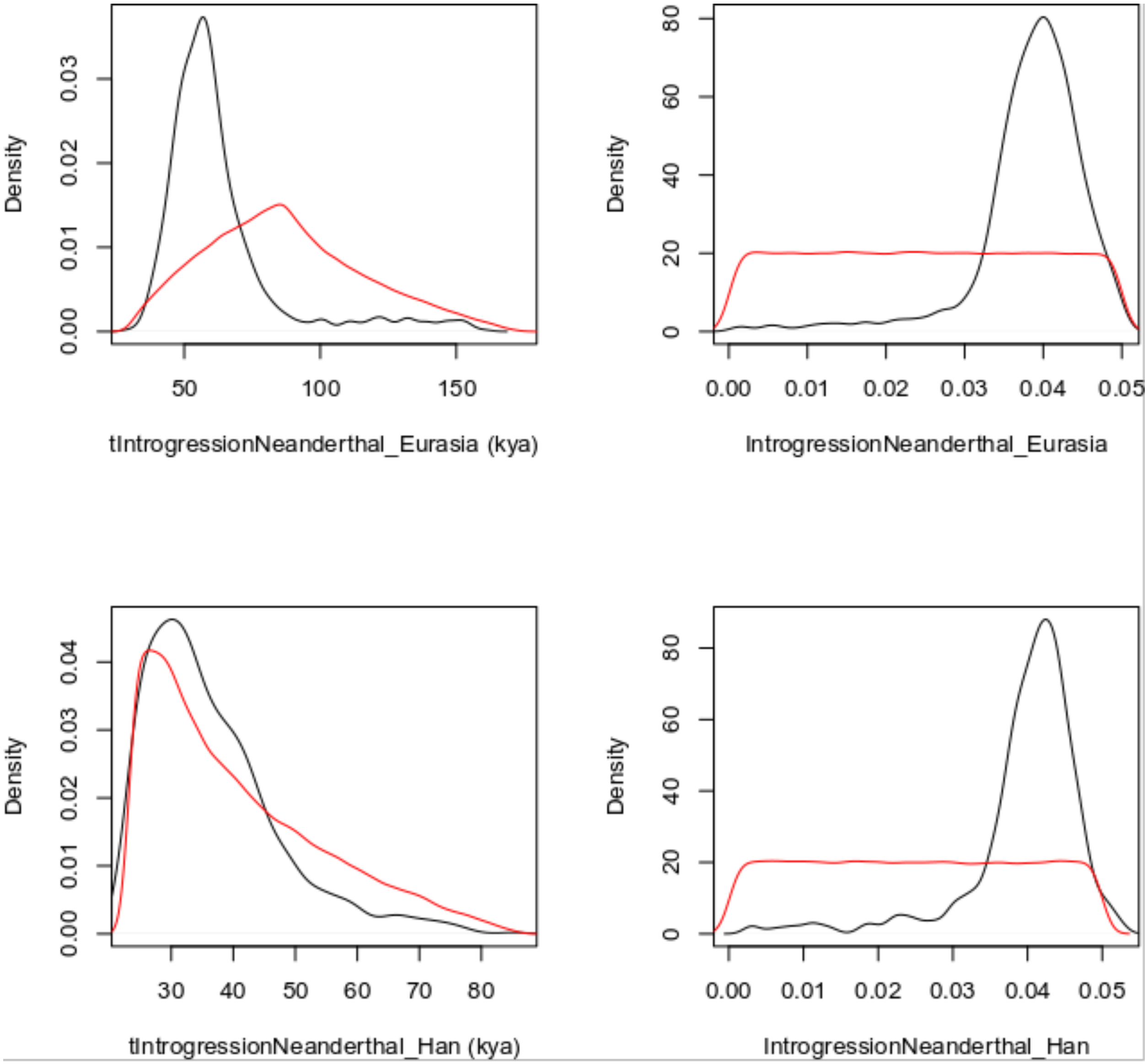
Density
0.00
0.01
0.02
0.03
50
100
150
tIntrogressionNeanderthal_Eurasia (kya)
Density
0
20
40
60
80
0.00
0.01
0.02
0.03
0.04
0.05
IntrogressionNeanderthal_Eurasia
Density
0.00
0.01
0.02
0.03
0.04
30
40
50
60
70
80
tIntrogressionNeanderthal_Han (kya)
Density
0
20
40
60
80
0.00
0.01
0.02
0.03
0.04
0.05
IntrogressionNeanderthal_Han

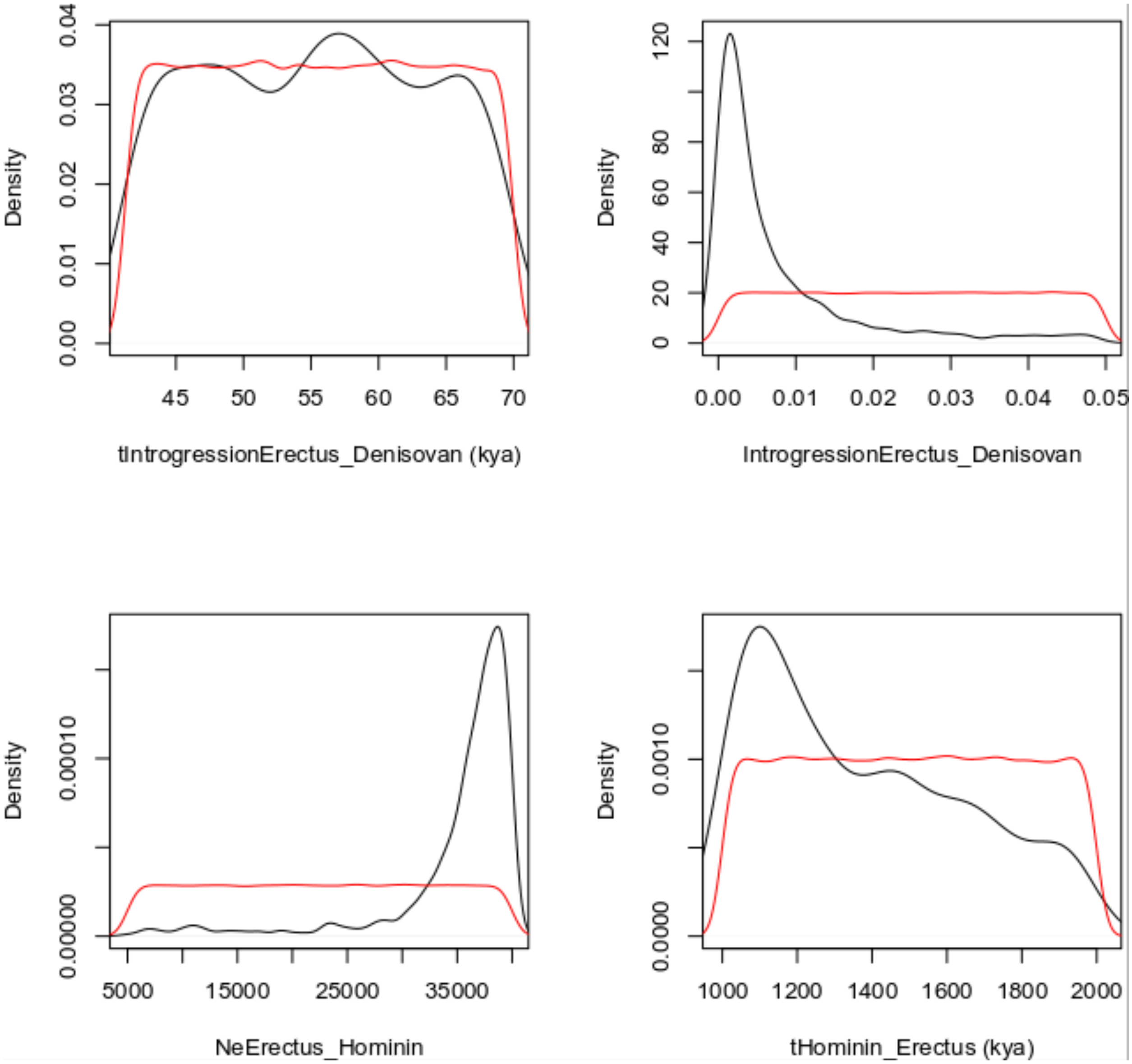
Density
0.00
0.01
0.02
0.03
0.04
45
50
55
60
65
70
tIntrogressionErectus_Denisovan (kya)
Density
0
20
40
60
80
120
0.00
0.01
0.02
0.03
0.04
0.05
IntrogressionErectus_Denisovan
Density
0.00000
0.00010
5000
15000
25000
35000
NeErectus_Hominin
Density
0.0000
0.0010
1000
1200
1400
1600
1800
2000
tHominin_Erectus (kya)

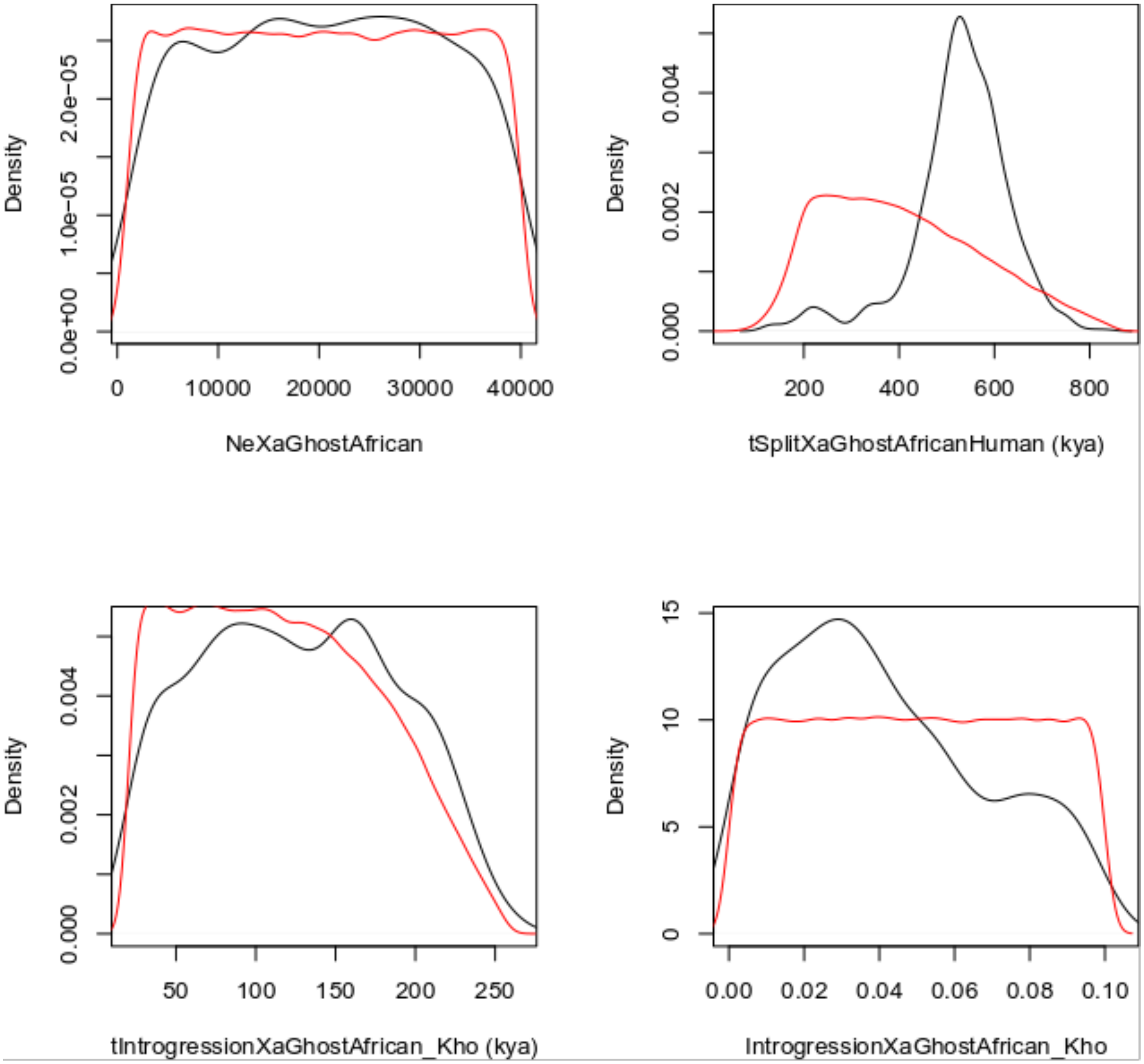
Density
0.0e+00
1.0e−05
2.0e−05
0
10000
20000
30000
40000
NeXaGhostAfrican
Density
0.000
0.002
0.004
200
400
600
800
tSplitXaGhostAfricanHuman (kya)
Density
0.000
0.002
0.004
50
100
150
200
250
tIntrogressionXaGhostAfrican_Kho (kya)
Density
0
5
10
15
0.00
0.02
0.04
0.06
0.08
0.10
IntrogressionXaGhostAfrican_Kho

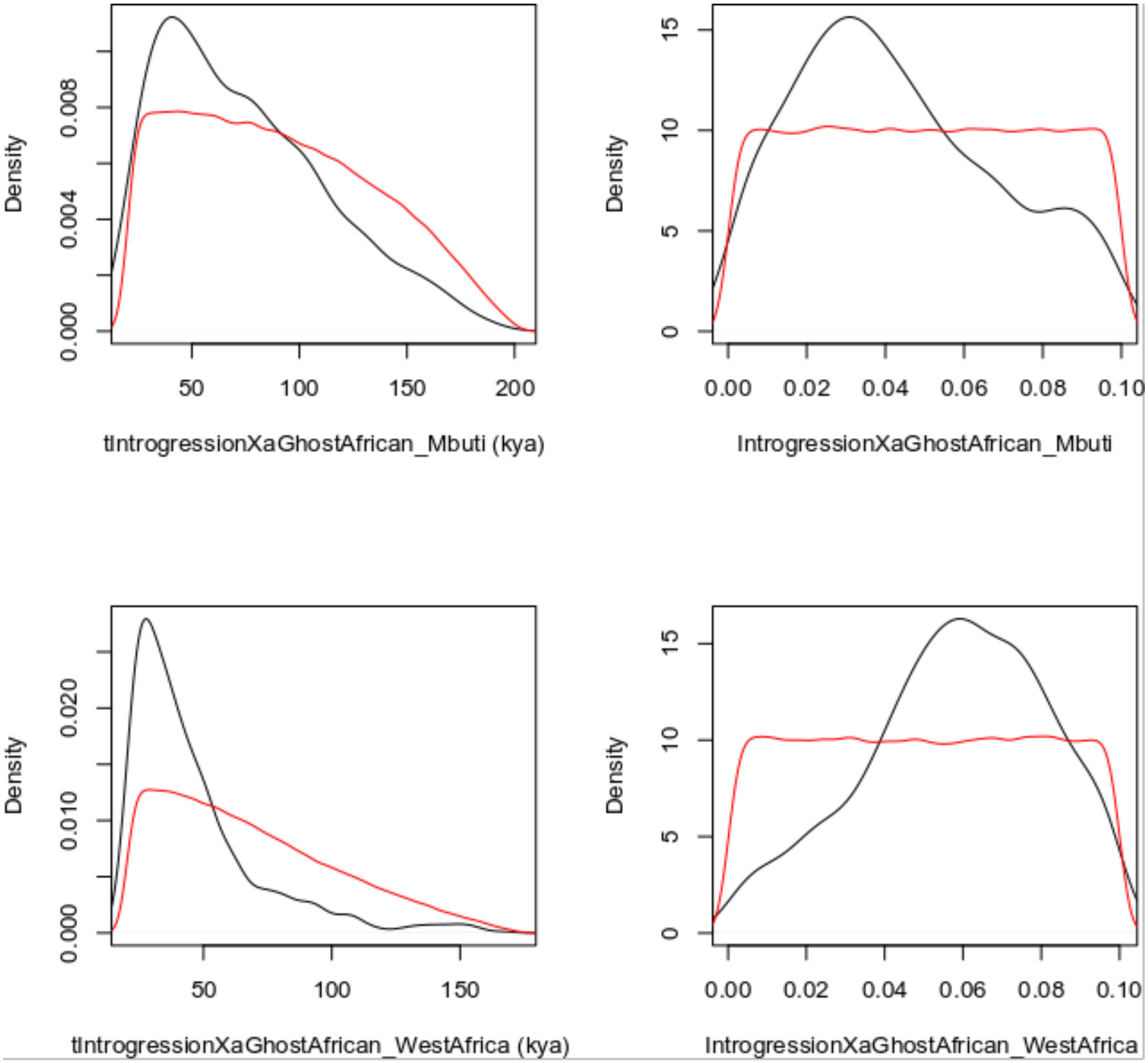
Density
0.000
0.004
0.008
50
100
150
200
tIntrogressionXaGhostAfrican_Mbuti (kya)
Density
0
5
10
15
0.00
0.02
0.04
0.06
0.08
0.10
IntrogressionXaGhostAfrican_Mbuti
Density
0.000
0.010
0.020
50
100
150
tIntrogressionXaGhostAfrican_WestAfrica (kya)
Density
0
5
10
15
0.00
0.02
0.04
0.06
0.08
0.10
IntrogressionXaGhostAfrican_WestAfrica

## References


1. Meyer M, Kircher M, Gansauge MT, Li H, Racimo F, Mallick S, Schraiber JG, Jay F, Prufer K, de Filippo C, et al: **A high-coverage genome sequence from an archaic Denisovan individual.** *Science* 2012, **338:**222-226.
2. Prufer K, Racimo F, Patterson N, Jay F, Sankararaman S, Sawyer S, Heinze A, Renaud G, Sudmant PH, de Filippo C, et al: **The complete genome sequence of a Neanderthal from the Altai Mountains.** *Nature* 2014, **505:**43-49.
3. Li H, Durbin R: **Fast and accurate short read alignment with Burrows-Wheeler transform.** *Bioinformatics* 2009, **25:**1754-1760.
4. DePristo MA, Banks E, Poplin R, Garimella KV, Maguire JR, Hartl C, Philippakis AA, del Angel G, Rivas MA, Hanna M, et al: **A framework for variation discovery and genotyping using next-generation DNA sequencing data.** *Nat Genet* 2011, **43:**491-498.
5. Behar DM, van Oven M, Rosset S, Metspalu M, Loogvali EL, Silva NM, Kivisild T, Torroni A, Villems R: **A "Copernican" reassessment of the human mitochondrial DNA tree from its root.** *Am J Hum Genet* 2012, **90:**675-684.
6. Li H, Handsaker B, Wysoker A, Fennell T, Ruan J, Homer N, Marth G, Abecasis G, Durbin R, Genome Project Data Processing S: **The Sequence Alignment/Map format and SAMtools.** *Bioinformatics* 2009, **25:**2078-2079.
7. Donmez N, Brudno M: **Hapsembler: An Assembler for Highly Polymorphic Genomes.** In *Research in Computational Molecular Biology*; *2011//; Berlin, Heidelberg*. Edited by Bafna V, Sahinalp SC. Springer Berlin Heidelberg; 2011: 38-52.
8. Altschul SF, Gish W, Miller W, Myers EW, Lipman DJ: **Basic local alignment search tool.** *J Mol Biol* 1990, **215:**403-410.
9. Katoh K, Toh H: **Recent developments in the MAFFT multiple sequence alignment program.** *Brief Bioinform* 2008, **9:**286-298.
10. Wei W, Ayub Q, Chen Y, McCarthy S, Hou Y, Carbone I, Xue Y, Tyler-Smith C: **A calibrated human Y-chromosomal phylogeny based on resequencing.** *Genome Res* 2013, **23:**388-395.
11. Skaletsky H, Kuroda-Kawaguchi T, Minx PJ, Cordum HS, Hillier L, Brown LG, Repping S, Pyntikova T, Ali J, Bieri T, et al: **The male-specific region of the human Y chromosome is a mosaic of discrete sequence classes.** *Nature* 2003, **423:**825-837.
12. van Oven M, Kayser M: **Updated comprehensive phylogenetic tree of global human mitochondrial DNA variation.** *Hum Mutat* 2009, **30:**E386-394.
13. Van Geystelen A, Decorte R, Larmuseau MH: **AMY-tree: an algorithm to use whole genome SNP calling for Y chromosomal phylogenetic applications.** *BMC Genomics* 2013, **14:**101.
14. Purcell S, Neale B, Todd-Brown K, Thomas L, Ferreira MA, Bender D, Maller J, Sklar P, de Bakker PI, Daly MJ, Sham PC: **PLINK: a tool set for whole-genome association and population-based linkage analyses.** *Am J Hum Genet* 2007, **81:**559-575.
15. Wang C, Szpiech ZA, Degnan JH, Jakobsson M, Pemberton TJ, Hardy JA, Singleton AB, Rosenberg NA: **Comparing spatial maps of human population-genetic variation using Procrustes analysis.** *Stat Appl Genet Mol Biol* 2010, **9:**Article 13.

16. Sokal RR, Oden NL, Barker JSF: **Spatial Structure in Drosophila buzzatii Populations: Simple and Directional Spatial Autocorrelation.** *The American Naturalist* 1987, **129:**122-142.
17. Rosenberg MS, Anderson CD: **PASSaGE: Pattern Analysis, Spatial Statistics and Geographic Exegesis. Version 2.** *Methods in Ecology and Evolution* 2011, **2:**229-232.
18. Rosenberg MS: **The bearing correlogram: A new method of analyzing directional spatial autocorrelation.** *Geographical Analysis* 2000, **32:**267-278.
19. McVean G: **A genealogical interpretation of principal components analysis.** *PLoS Genet* 2009, **5:**e1000686.
20. Alexander DH, Novembre J, Lange K: **Fast model-based estimation of ancestry in unrelated individuals.** *Genome Res* 2009, **19:**1655-1664.
21. Afgan E, Baker D, van den Beek M, Blankenberg D, Bouvier D, Cech M, Chilton J, Clements D, Coraor N, Eberhard C, et al: **The Galaxy platform for accessible, reproducible and collaborative biomedical analyses: 2016 update.** *Nucleic Acids Res* 2016, **44:**W3-W10.
22. Kim HL, Ratan A, Perry GH, Montenegro A, Miller W, Schuster SC: **Khoisan hunter-gatherers have been the largest population throughout most of modern-human demographic history.** *Nat Commun* 2014, **5:**5692.
23. Patterson N, Moorjani P, Luo Y, Mallick S, Rohland N, Zhan Y, Genschoreck T, Webster T, Reich D: **Ancient admixture in human history.** *Genetics* 2012, **192:**1065-1093.
24. Kunsch HR: **The Jackknife and the Bootstrap for General Stationary Observations.** *Ann Statist* 1989, **17:**1217-1241.
25. Pickrell JK, Patterson N, Loh PR, Lipson M, Berger B, Stoneking M, Pakendorf B, Reich D: **Ancient west Eurasian ancestry in southern and eastern Africa.** *Proc Natl Acad Sci U S A* 2014, **111:**2632-2637.
26. Gallego Llorente M, Jones ER, Eriksson A, Siska V, Arthur KW, Arthur JW, Curtis MC, Stock JT, Coltorti M, Pieruccini P, et al: **Ancient Ethiopian genome reveals extensive Eurasian admixture throughout the African continent.** *Science* 2015, **350:**820-822.
27. Sikora M, Carpenter ML, Moreno-Estrada A, Henn BM, Underhill PA, Sanchez-Quinto F, Zara I, Pitzalis M, Sidore C, Busonero F, et al: **Population genomic analysis of ancient and modern genomes yields new insights into the genetic ancestry of the Tyrolean Iceman and the genetic structure of Europe.** *PLoS Genet* 2014, **10:**e1004353.
28. Li H, Durbin R: **Inference of human population history from individual whole-genome sequences.** *Nature* 2011, **475:**493-496.
29. Delaneau O, Zagury JF: **Haplotype inference.** *Methods Mol Biol* 2012, **888:**177-196.
30. Maples BK, Gravel S, Kenny EE, Bustamante CD: **RFMix: a discriminative modeling approach for rapid and robust local-ancestry inference.** *Am J Hum Genet* 2013, **93:**278-288.
31. Reich D, Green RE, Kircher M, Krause J, Patterson N, Durand EY, Viola B, Briggs AW, Stenzel U, Johnson PL, et al: **Genetic history of an archaic hominin group from Denisova Cave in Siberia.** *Nature* 2010, **468:**1053-1060.
32. Rogers AR, Bohlender RJ, Huff CD: **Early history of Neanderthals and Denisovans.** *Proc Natl Acad Sci U S A* 2017, **114:**9859-9863.

33. Mallick S, Li H, Lipson M, Mathieson I, Gymrek M, Racimo F, Zhao M, Chennagiri N, Nordenfelt S, Tandon A, et al: **The Simons Genome Diversity Project: 300 genomes from 142 diverse populations.** *Nature* 2016, **538:**201-206.
34. Sankararaman S, Mallick S, Patterson N, Reich D: **The Combined Landscape of Denisovan and Neanderthal Ancestry in Present-Day Humans.** *Curr Biol* 2016, **26:**1241-1247.
35. Tavare S, Balding DJ, Griffiths RC, Donnelly P: **Inferring coalescence times from DNA sequence data.** *Genetics* 1997, **145:**505-518.
36. Bertorelle G, Benazzo A, Mona S: **ABC as a flexible framework to estimate demography over space and time: some cons, many pros.** *Mol Ecol* 2010, **19:**2609-2625.
37. Beaumont MA, Rannala B: **The Bayesian revolution in genetics.** *Nat Rev Genet* 2004, **5:**251-261.
38. Aeschbacher S, Beaumont MA, Futschik A: **A novel approach for choosing summary statistics in approximate Bayesian computation.** *Genetics* 2012, **192:**1027-1047.
39. Blum MGB, Nunes MA, Prangle D, Sisson SA: **A Comparative Review of Dimension Reduction Methods in Approximate Bayesian Computation.** *Statist Sci* 2013, **28:**189-208.
40. Mondal M, Bertranpetit J, Lao O: **Approximate Bayesian computation with deep learning supports a third archaic introgression in Asia and Oceania.** *Nat Commun* 2019, **10:**246.
41. Jiang B, Wu T-Y, Zheng C, Wong WH: **LEARNING SUMMARY STATISTIC FOR APPROXIMATE BAYESIAN COMPUTATION VIA DEEP NEURAL NETWORK.** *Statistica Sinica* 2017, **27:**1595-1618.
42. Gutenkunst RN, Hernandez RD, Williamson SH, Bustamante CD: **Inferring the joint demographic history of multiple populations from multidimensional SNP frequency data.** *PLoS Genet* 2009, **5:**e1000695.
43. Excoffier L, Dupanloup I, Huerta-Sanchez E, Sousa VC, Foll M: **Robust demographic inference from genomic and SNP data.** *PLoS Genet* 2013, **9:**e1003905.
44. Bock C, Walter J, Paulsen M, Lengauer T: **CpG island mapping by epigenome prediction.** *PLoS Comput Biol* 2007, **3:**e110.
45. Excoffier L, Foll M: **fastsimcoal: a continuous-time coalescent simulator of genomic diversity under arbitrarily complex evolutionary scenarios.** *Bioinformatics* 2011, **27:**1332-1334.
46. Lipson M, Loh PR, Sankararaman S, Patterson N, Berger B, Reich D: **Calibrating the Human Mutation Rate via Ancestral Recombination Density in Diploid Genomes.** *PLoS Genet* 2015, **11:**e1005550.
47. Elliott DL: **A better Activation Function for Artificial Neural Networks.** 1993.
48. Breiman L: **Heuristics of Instability and Stabilization in Model Selection.** *The Annals of Statistics* 1996, **24:**2350-2383.
49. Blum KCaOFaMGB: **abc: an R package for approximate Bayesian computation (ABC).** *Methods in Ecology and Evolution* 2012.
50. Excoffier L, Estoup A, Cornuet JM: **Bayesian analysis of an admixture model with mutations and arbitrarily linked markers.** *Genetics* 2005, **169:**1727-1738.
51. Beaumont MA, Zhang W, Balding DJ: **Approximate Bayesian computation in population genetics.** *Genetics* 2002, **162:**2025-2035.

52. Hamilton G, Currat M, Ray N, Heckel G, Beaumont M, Excoffier L: **Bayesian estimation of recent migration rates after a spatial expansion.** *Genetics* 2005, **170:**409-417.
53. Fenner JN: **Cross-cultural estimation of the human generation interval for use in genetics-based population divergence studies.** *Am J Phys Anthropol* 2005, **128:**415-423.
